# Processing, Microstructural Evolution and Engineering Performance of High-Entropy Alloys: A Review

**DOI:** 10.3390/ma19173807

**Published:** 2026-09-07

**Authors:** Jingwen Zhang, Jingteng Xue, Jiaying Chen, Tao Xia, Wei Zhang, Wentao Zhou, Yong Liu, Jingchuan Zhu

**Affiliations:** School of Materials Science and Engineering, Harbin Institute of Technology, Harbin 150001, China; 25s109140@stu.hit.edu.cn (J.Z.);

**Keywords:** high-entropy alloys, multi-principal-element alloys, phase stability, microstructural evolution, multiscale characterization, service performance

## Abstract

High-entropy alloys (HEAs) and multi-principal-element alloys (MPEAs) provide broad compositional flexibility for regulating phase stability, microstructure, and properties. However, nominal composition and average phase constitution alone are insufficient to describe the actual material state formed during processing and service. This review summarizes the thermodynamic and diffusion-kinetic basis of phase formation and compares five representative fabrication routes, including mechanical alloying, vacuum melting, severe plastic deformation, magnetron sputtering, and additive manufacturing. Particular attention is given to the effects of processing history on grain structure, texture, elemental segregation, defects, phase constitution, and local chemical order. Computational methods and multiscale characterization techniques are also discussed in relation to the identification and interpretation of processing-dependent material states. Current studies indicate that alloys with identical nominal compositions can exhibit different microstructures and properties because of differences in thermal history, strain path, elemental redistribution, defect populations, and post-processing conditions. The review further examines strength and ductility, corrosion resistance, oxidation resistance, irradiation tolerance, and catalytic performance, with emphasis on the evolution of microstructure and surface state under service conditions. These results indicate that reliable evaluation of HEAs and MPEAs requires consideration of processing reproducibility, structural heterogeneity, and long-term stability rather than isolated peak properties. This processing–structure–service perspective provides a basis for more reliable comparison, selection, and engineering assessment of HEAs and MPEAs under application-relevant conditions. Future research should focus on reproducible fabrication, integration of computational prediction with experimental validation, multiscale assessment of structural evolution, long-term service performance, scalable processing, and sustainable alloy design.

## 1. Introduction

High-entropy alloys and multi-principal-element alloys broaden the conventional alloy-design paradigm by distributing compositional control among several principal elements. The multi-principal-element concept introduced by Yeh et al. and the equiatomic CoCrFeMnNi alloy reported by Cantor et al. established the foundation of the field [1,2]. Their importance lies not simply in a large element count, but in the expanded freedom to regulate phase constitution and local chemical environments [3]. Although early work emphasized configurational entropy, reported single-phase HEAs occupy only a small fraction of the available composition space [4], and even the classic CrMnFeCoNi alloy can transform from FCC to HCP under pressure [5]. Equiatomic composition and high ideal entropy therefore cannot determine the final state by themselves. Phase formation instead reflects the combined effects of thermodynamic driving forces and the diffusion kinetics that control whether transformations can proceed over the available temperature and time.

Fabrication converts nominal composition into an actual microstructure. Mechanical alloying promotes solid-state mixing through repeated deformation and fracture, whereas vacuum melting involves liquid-state mixing followed by solidification [6,7]. Magnetron sputtering and additive manufacturing impose more strongly nonequilibrium thermal histories and can retain refined or metastable structures [8,9]. Parameter changes within a route alter phase evolution [10], while local thermal histories in additive manufacturing can create different states within one component [11]. Compositional changes can also redistribute elements locally and modify the average phase constitution [12]. Reliable interpretation consequently requires characterization across length scales. X-ray diffraction identifies the principal crystal structures, but an average FCC or BCC phase does not imply random elemental occupation. Scattering experiments reveal deviations from ideal randomness [13], chemical short-range order has been reported in CrCoNi [14], and atomic-resolution methods provide more direct evidence of local arrangements [15]. In situ diffraction can further track structural changes under external fields [16], while corrosion and oxidation studies show continuing evolution of elemental distributions and surface chemical states [17,18]. Average phase constitution, local order, elemental partitioning, surface chemistry, and dynamic evolution must therefore be connected rather than treated as separate descriptors.

Property evaluation must likewise retain the material state and service condition. CrCoNi can become stronger at low temperature while preserving exceptional damage tolerance through sustained dislocation activity and nanoscale twinning [19], but HEAs have no universal strength –ductility mechanism because their phases and deformation modes differ [20]. Manufacturing defects and microstructural directionality introduce additional anisotropy, particularly in additively manufactured components [21]. At high temperature, oxidation resistance depends on both the oxidation rate and the integrity of the protective scale [22]. Under irradiation, multielement local environments can modify defect migration and accumulation, but suppressing microscopic defects does not automatically ensure long-term property retention [23]. In catalysis, diverse nearest-neighbor configurations can regulate intermediate adsorption, yet practical performance is governed by the surface state that develops during operation [24]. This review therefore follows the progression from phase stability and diffusion, through fabrication and multiscale characterization, to mechanical, environmental, irradiation, and catalytic performance under relevant service conditions.

**Figure 1:**
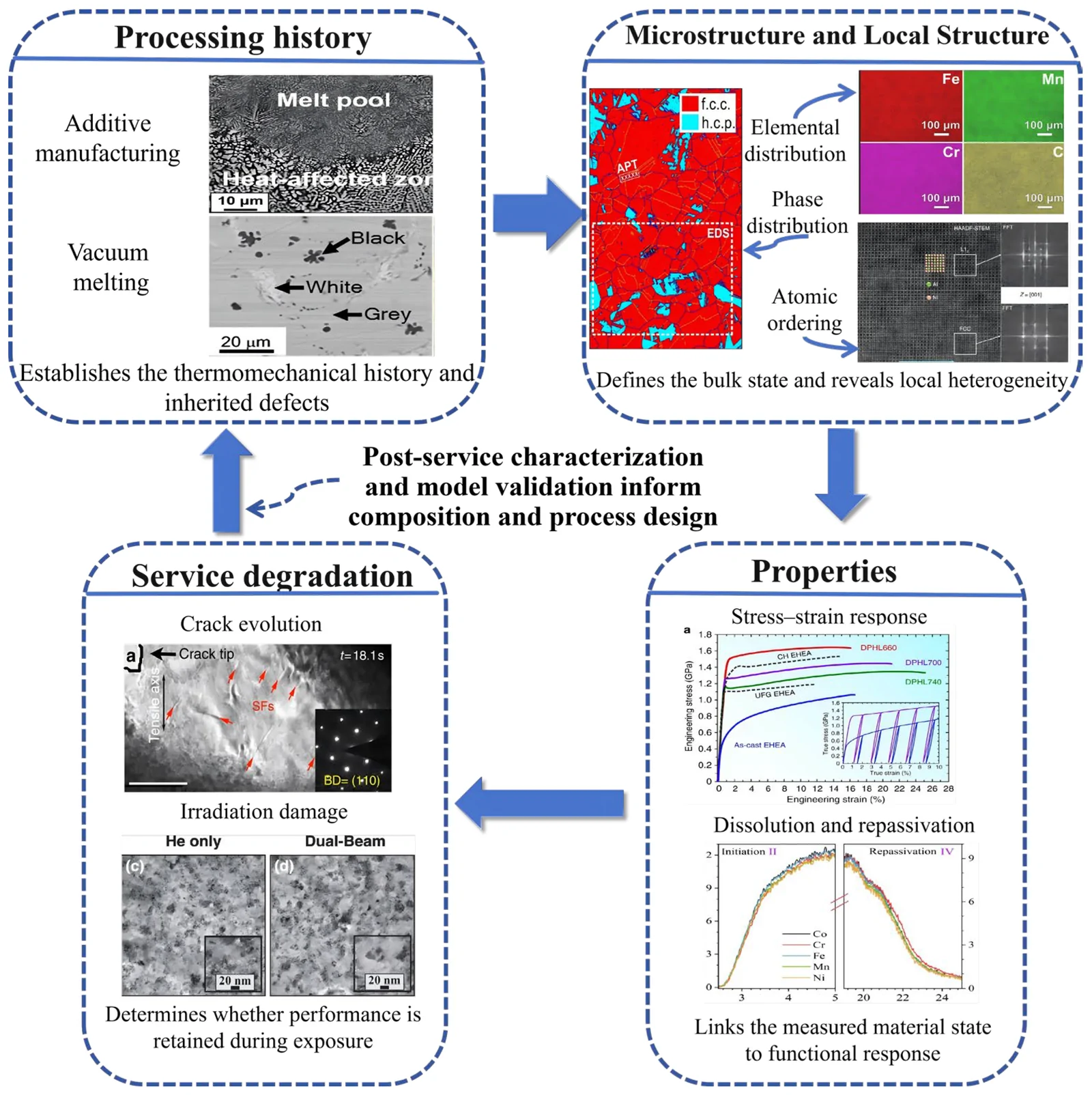
Schematic illustration of the relationships among processing history, microstructural state, local structure, engineering properties, and service-induced degradation in high-entropy alloys. Representative images are adapted from Refs. [7,34,35,36,37,38,39,40]. The red arrows indicate stacking faults (SFs) near the crack tip.

Recent reviews have summarized HEA fabrication and surface modification, processing–microstructure–mechanical property relationships, additive manufacturing, high-temperature behavior, and corrosion or oxidation in harsh environments [25,26,27,28,29]. More recent reviews have extended this coverage to MPEA coatings and their deposition routes and industrial applications [30]; processing-dependent corrosion mechanisms and passive-film stability in 3D transition-metal HEAs [31]; HEA electrocatalysis integrating synthesis, surface chemistry, computational design, and artificial intelligence-assisted screening [32]; and MPEA-based films and coatings with emphasis on nanoscale architecture, interface engineering, mechanical response, corrosion tolerance, and thermal stability [33]. Their principal scopes and boundaries are compared in Table 1. Taken together, these studies demonstrate that the literature has expanded rapidly across processing, structural, environmental, and functional topics. However, most existing reviews remain organized around a particular fabrication route, material form, property category, or application field. Consequently, several cross-cutting issues remain comparatively fragmented: systematic comparison of different fabrication routes using common microstructural descriptors; distinction between nominal composition and the actual material state produced by a specific processing history; fabrication reproducibility across positions, specimens, and batches; and integration of multiscale structural evidence with long-term mechanical, environmental, irradiation, and catalytic degradation. To address these gaps, the present review establishes a continuous framework connecting processing history, the resulting microstructural and local structural state, engineering performance, and service-induced degradation. Rather than treating nominal composition or average phase constitution as sufficient descriptors, it integrates phase stability, diffusion behavior, fabrication-dependent microstructure, multiscale characterization, and service-related evolution to explain why nominally similar HEAs and MPEAs may exhibit different properties and reliability under practical conditions. The overall processing–structure–service framework is summarized in Figure 1.

## 2. Theory of High-Entropy Alloys

### 2.1. Definitions of High-Entropy and Multi-Principal-Element Alloys

HEAs were initially defined primarily by their multiple principal elements and high configurational entropy. However, as non-equiatomic, medium-entropy, and multiphase alloy systems have expanded, a single compositional or entropy criterion can no longer encompass the full range of current research subjects [1,2]. Existing definitions are mainly composition-based or entropy-based. The former usually requires an alloy to contain at least five principal elements, each with an atomic fraction ranging from approximately 5% to 35%. The latter uses ideal configurational entropy as the criterion and generally classifies systems with ΔS_conf_ values of at least 1.5R as high-entropy alloys [41]. For an ideal substitutional solid solution in which different elements randomly occupy equivalent lattice sites, the configurational entropy can be expressed as follows:

(1) $$\Delta S_{\text{conf}}\;=\;-R\sum_{i=1}^{N}c_{i}\ln c_{i}$$

where c_i_ is the mole fraction of the ith element and N is the number of components. Here, R represents the gas constant (8.314 J mol^−1^ K^−1^), and 1.5R is the commonly adopted configurational entropy threshold for defining high-entropy alloys. For an equiatomic composition, configurational entropy reaches its maximum value for the given number of components:

(2) $$\Delta S_{\text{conf}}\;=\;R\ln N$$

Equations (1) and (2) assume completely random atomic occupation and therefore describe ideal configurational entropy rather than directly measured chemical order [42,43]. In this review, HEA retains the conventional composition-based and entropy-based definitions given above, whereas MPEA denotes the broader class of alloys containing several principal elements without requiring an equiatomic composition, an ideal configurational entropy of at least 1.5R, a single-phase structure, or random atomic occupation. HEAs are therefore treated as a subset of MPEAs, and the two terms are not used interchangeably. HEA is used for alloys that satisfy at least one of the conventional HEA criteria defined above. MPEA is used for broader category-level statements that also encompass medium-entropy and other compositionally complex alloys. When an individual study uses a different designation, its original terminology is retained only when reporting that specific result.

### 2.2. Thermodynamics

The phase stability of HEAs is governed by competition in Gibbs free energy among disordered solid solutions, ordered phases, intermetallic compounds, and multiphase states. The competition between enthalpy and entropy during mixing can be expressed as follows:

(3) $$\Delta G_{\text{mix}}\;=\;\Delta H_{\text{mix}}\;-\;T\Delta S_{\text{mix}}$$

where ΔG_mix_, ΔH_mix_, and ΔS_mix_ are the Gibbs free energy, enthalpy, and entropy of mixing, respectively, and T is the absolute temperature. As temperature increases, the −TΔS_mix_ term can lower the free energy of a disordered phase. However, the phase is stable at equilibrium only when its free energy is lower than that of every competing phase and phase combination. A negative ΔG_mix_ value relative to the unmixed pure elements indicates only that mixing is thermodynamically favorable and does not mean that a single random solid solution has the lowest free energy [44].

For two candidate states, α and β, of the same composition, the driving force for transformation can be written as follows:

(4) $$\Delta G_{\alpha \to \beta \;}=\;G_{\beta }\;-\;G_{\alpha }$$

When ΔG_α→β_ is negative, state β has the lower free energy at the corresponding temperature and pressure. When the free-energy difference is small, interfacial energy, defect structures, and limited diffusion time may allow a metastable state to persist. Phase-stability analysis should therefore compare the free energies of explicitly defined candidate states rather than draw conclusions from a single mixing-entropy value [44]. The entropy of mixing can be further decomposed into configurational, vibrational, magnetic, and electronic contributions:

(5) $$\Delta S_{\text{mix}}\;=\;\Delta S_{\text{conf}}\;+\;\Delta S_{\text{vib}}\;+\;\Delta S_{\text{mag}}\;+\;\Delta S_{\text{elec}}$$

Ideal configurational entropy is a composition-derived thermodynamic quantity rather than an experimental measure of chemical order. A high value of ideal configurational entropy therefore does not demonstrate random atomic occupation or exclude ordered and multiphase states. Enthalpic interactions can favor specific atomic pairs and competing ordered structures even when the ideal configurational entropy is high [45,46]. Vibrational and electronic contributions, together with the temperature-dependent Gibbs energies of the relevant competing phases, further modify phase stability [47,48].

(6) $$\Delta H_{\text{mix}}\;\approx \;4\sum_{i<j}c_{i}c_{j}\Delta H_{ij}$$

where ΔH_ij_ is the enthalpy of mixing of an equiatomic liquid binary i–j alloy. A strongly negative enthalpy of mixing indicates a strong chemical affinity between unlike atoms and favors their association, but it can also enhance preferential coordination of specific atomic pairs, thereby driving the formation of ordered B2 or L1_2_ phases or other intermetallic compounds. A positive enthalpy of mixing may increase the tendency toward segregation and phase separation. Because this approximate model does not fully account for many-body interactions, bonding characteristics in different phases, or temperature effects, it is more suitable for a preliminary assessment of chemical compatibility among elements than for accurate prediction of the final phase structure [49,50].

To describe the combined contributions of entropy, chemical interactions, and atomic-size differences, the Ω parameter and atomic-size mismatch parameter δ are commonly used. These parameters have been widely applied as empirical criteria for preliminary phase-selection analysis in HEAs based on thermodynamic contributions and atomic-size differences [51,52].

(7) $$\Omega =\frac{{\bar{T}}_{m}\Delta S_{\text{conf}}}{\left|\Delta H_{\text{mix}}\right|}$$

(8) $$\delta =100{\left[\sum_{i}c_{i}{\left(1-\frac{r_{i}}{\bar{r}}\right)}^{2}\right]}^{\frac{1}{2}}$$

where the weighted mean melting point and mean atomic radius are, respectively:

(9) $${\bar{T}}_{m}\;=\;\sum_{i}c_{i}T_{m,i}$$

(10) $$\bar{r}=\sum_{i}c_{i}r_{i}$$

where T_m,i_ and r_i_ are the melting point and atomic radius of the ith element, respectively. Based on existing experimental data, Yang and Zhang proposed that solid-solution formation is favored when Ω is at least 1.1 and δ is no greater than 6.6% [51]. These empirical thresholds can be used for preliminary compositional screening, but they cannot readily distinguish a disordered A2 phase from an ordered B2 phase, identify chemical short-range order, or describe metastable structures retained during nonequilibrium processing. The final phase state of an alloy must therefore be determined by combining phase-equilibrium calculations, heat-treatment experiments, and multiscale structural characterization. The entropy term is generally more important at high temperatures. As temperature decreases, enthalpy-driven ordering, precipitation, or phase decomposition may gradually become dominant. Rapid solidification and thin-film deposition can limit extensive elemental redistribution by shortening the high-temperature diffusion time, whereas mechanical alloying shortens atomic migration distances through mechanical mixing and the introduction of a high density of defects. Although these nonequilibrium processes operate through different mechanisms, each can retain metastable phases or supersaturated solid solutions at room temperature [52].

**Figure 2:**
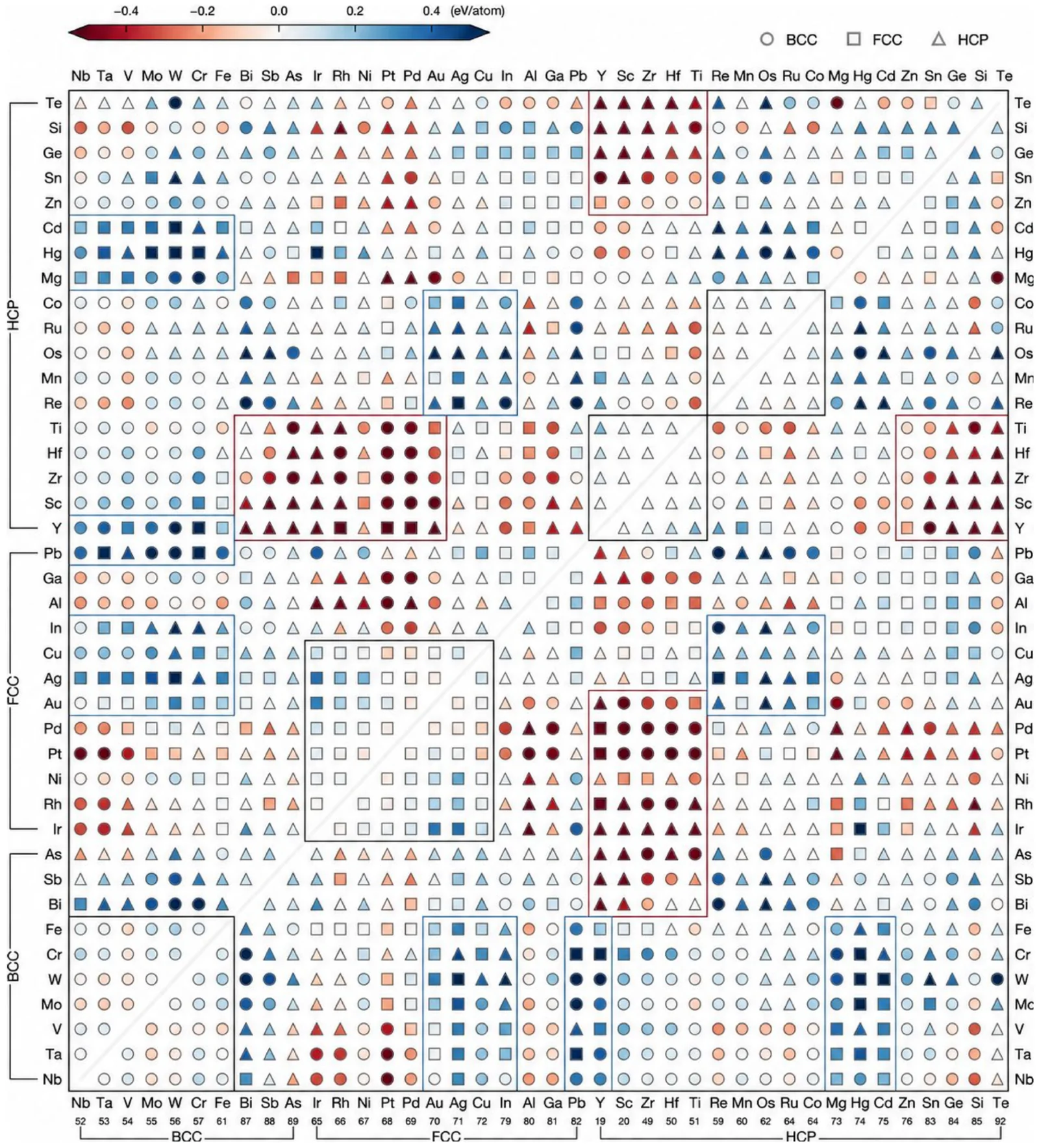
DFT-calculated formation enthalpies and ground-state structures of binary solid solutions formed by pairwise combinations of 40 alloying elements. Circles, squares, and triangles denote BCC, FCC, and HCP structures, respectively [4]. Black boxes group element pairs whose binary solid solutions have the same ground-state structure; red and blue boxes indicate strong favoring (ΔHf < −0.2 eV/atom) and strong disfavoring (ΔHf > 0.2 eV/atom) of mixing, respectively.

These mechanisms should be separated when interpreting a processed alloy. Configurational entropy changes equilibrium free-energy competition, whereas chemical ordering is a measurable local or long-range structural state governed by preferential atomic interactions [15,42]. Diffusion kinetics determines whether an equilibrium tendency can be approached within the available time. Rapid solidification, physical-vapor deposition, mechanical alloying, and severe plastic deformation instead impose path-dependent states by limiting redistribution or generating nonequilibrium defects [53,54]. Phase selection must therefore be described using both thermodynamic driving forces and the complete time –temperature –strain history rather than a configurational-entropy value alone. The relationship between pairwise chemical interactions and structural preference is illustrated in Figure 2.

Figure 2 summarizes the DFT-calculated formation enthalpies and ground-state structures of binary solid solutions formed by pairwise combinations of 40 alloying elements [4]. Circles, squares, and triangles represent BCC, FCC, and HCP structures, respectively. The color scale gives the formation enthalpy relative to the ground-state elemental phases in eV per atom. Red corresponds to negative formation enthalpy and a favorable tendency for pairwise mixing, blue corresponds to positive formation enthalpy and an unfavorable tendency, and colors near white indicate a weak enthalpic preference. Black outlines group element pairs with the same predicted ground-state structure, while red and blue outlines identify strongly favorable and strongly unfavorable mixing regions, respectively. The map therefore visualizes pairwise chemical interactions and structural preferences, but it cannot alone determine the phase stability of a multicomponent alloy because competing intermetallic phases, temperature, and kinetic accessibility must also be considered. Even when a phase transformation is thermodynamically favorable, it can occur during processing only if atoms have sufficient mobility to redistribute over the required distance within the available time. The final phase state therefore reflects both the thermodynamic driving force and the atomic mobility established by the processing temperature and duration.

### 2.3. Diffusion Kinetics

A thermodynamic driving force does not mean that a phase transformation can be completed within a practical time. Processes such as ordering, precipitation, and phase separation all depend on atomic migration. The diffusion rate therefore directly affects microstructural evolution and the final phase state. The temperature dependence of the bulk diffusion coefficient is commonly described by the Arrhenius relationship:

(11) $$D\;={\;D}_{0}\text{exp}\left(-\frac{Q}{\mathrm{RT}}\right)$$

where D_0_ is the diffusion pre-exponential factor, Q is the apparent activation energy for diffusion, R is the gas constant, and T is the absolute temperature. D_0_ and Q depend strongly on the diffusing species and the structural state of the material. Diffusion data from different studies should therefore be compared under similar phase structures, diffusion paths, and temperature ranges. When the diffusion coefficient is approximately constant, the characteristic distance for one-dimensional diffusion can be estimated as follows:

(12) $$l\;\approx \;{\left(2\mathrm{Dt}\right)}^{\frac{1}{2}}$$

This relationship shows that the final microstructure depends on both the diffusion coefficient and diffusion time. Prolonged high-temperature treatment can promote elemental redistribution, whereas the short diffusion times associated with rapid solidification, mechanical alloying, and thin-film deposition favor the retention of nonequilibrium solid solutions or metastable phases.

Early tracer-diffusion experiments measured low component diffusion coefficients in CoCrFeMnNi and led to the proposal of a sluggish diffusion effect [55]. Subsequent studies did not find that such retardation applied universally to all components. For example, different elements in FCC AlCoCrFeNi systems exhibit different diffusion behaviors [56], and no systematic diffusion slowdown caused by increased configurational entropy was observed in Al_0.25_CoCrFeNi [57]. Direct tracer experiments further showed pronounced elemental and compositional dependence [58,59], while the apparent degree of retardation also changed with the temperature scale used for comparison [60]. These results show that diffusion retardation varies with element, composition, phase, and comparison basis rather than increasing uniformly with configurational complexity.

These differences are also associated with the complex local atomic environments of MPEAs. Atomistic calculations show that different local configurations produce different atomic migration barriers, making the macroscopic diffusion coefficient a statistical outcome of multiple local jump processes [53]. Thus, even when the average crystal structure is the same, the migration environments experienced by different elements are not identical. Short-circuit diffusion paths such as grain boundaries also involve migration mechanisms different from those of intragranular bulk diffusion. Radiotracer experiments show that Ni grain-boundary diffusion in CoCrFeNi and CoCrFeMnNi did not decrease continuously over the entire temperature range as the number of components increased [61]. Subsequent investigations indicate that some low-temperature grain-boundary diffusion behavior in CoCrFeMnNi may also be related to local structural changes and phase decomposition [62]. Diffusion must therefore be analyzed in relation to the specific element, phase state, and migration path rather than be inferred only from the number of elements in an alloy. Systematic assessments of existing diffusion data likewise show that sluggish diffusion is strongly system- and condition-dependent [63] and that no universal slowdown applies to every HEA and every component [64].

The influence of diffusion kinetics on microstructure formation is particularly direct during nonequilibrium solidification. In situ solidification studies of CoCrFeNi and its ternary CrFeNi and CoCrNi subsystems indicate that limited solute diffusion affects elemental redistribution near the solid–liquid interface and thereby participates in phase selection during solidification [54]. A single-phase or refined microstructure produced by rapid solidification should therefore not be attributed simply to a high cooling rate. Solute redistribution during interface advancement and subsequent solid-state evolution must also be considered.

### 2.4. Core Effects

The high-entropy, lattice-distortion, sluggish-diffusion, and cocktail effects formed the classic conceptual framework of early HEA research [44]. They describe phenomena at different physical levels and do not constitute a universal causal chain. Current evidence instead treats them as hypotheses that must be tested against phase constitution, local chemical environment, defect structure, diffusion path, and measurement condition [65]. Recent atomically resolved and three-dimensional studies have revealed widespread but alloy-dependent chemical short-range order in CrCoNi-related and other MPEAs [15,42]. These developments shift the emphasis from assuming ideal randomness to determining the actual local structure.

Configurational entropy contributes to free-energy competition but cannot independently stabilize a single disordered phase. Element-specific enthalpic interactions may instead favor ordering, decomposition, or segregation as temperature changes. Atomic-size mismatch is likewise only a compositional proxy for lattice distortion. Total-scattering measurements and first-principles calculations show that local strain depends on the particular atomic neighborhood and cannot be inferred from composition or XRD peak broadening alone [66,67]. Diffusion retardation depends on the species, phase, pathway, and temperature rather than representing an intrinsic property shared by every HEA [55,64]. The cocktail effect is even less specific because it labels a nonlinear overall response without identifying a unique physical variable. Such a response must be traced to measured phase transformations, interfaces, defects, and deformation mechanisms, as illustrated by composition-dependent structural changes in FeCoNi(CuAl)_X_ alloys [68,69].

**Figure 3:**
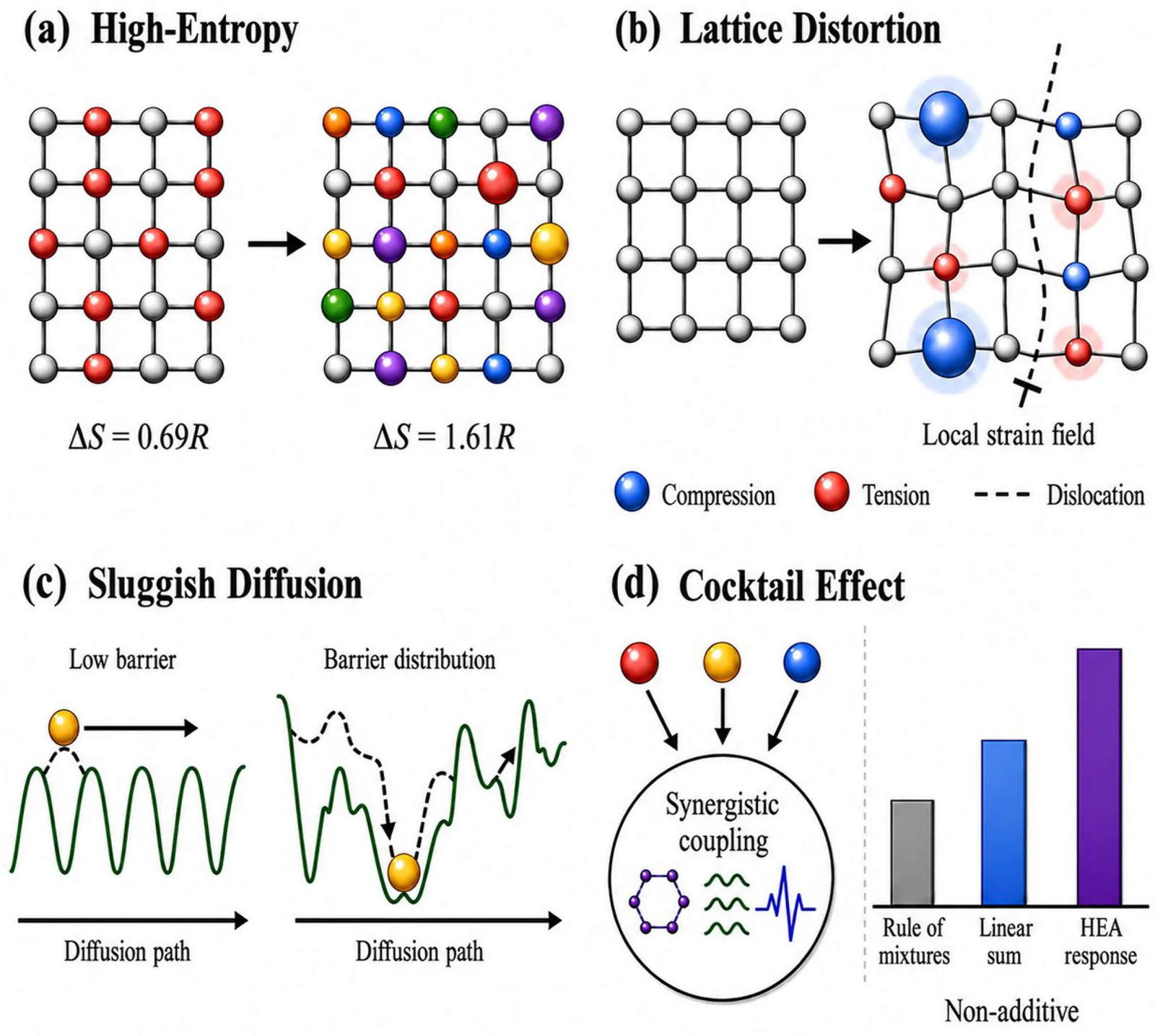
Schematic illustration of the four classic core effects in HEAs. (**a**) High-entropy effect. (**b**) Lattice distortion. (**c**) Sluggish diffusion. (**d**) Cocktail effect. The different colors represent different constituent elements. The dashed curves in panel (**c**) schematically indicate atomic hopping across local diffusion barriers.

The modern value of the four effects is therefore diagnostic rather than predictive. They provide questions about entropy, local strain, mobility, and multielement interactions, while the answers require direct thermodynamic, structural, kinetic, and property evidence for the material state under study. Figure 3 retains the classical framework for orientation, and Table 2 summarizes the physical meaning and principal limitation of each effect.

### 2.5. Computational and Experimental Integration

Computational methods provide complementary descriptions of HEAs and MPEAs because no single method spans atomic interactions, microstructural evolution, and macroscopic properties. CALPHAD uses assessed Gibbs-energy descriptions to evaluate equilibrium or metastable phase stability, phase fractions, and thermodynamic driving forces across composition and temperature ranges. Conventional equilibrium CALPHAD alone can identify candidate phases and transformation conditions, but it does not directly reproduce the segregation, defect structures, or local order generated by a specific processing history. Combining CALPHAD outputs with machine learning has improved phase selection across large compositional spaces while retaining thermodynamic constraints [71]. Density functional theory resolves relative phase energies, bonding, elastic response, and local chemical preferences for selected atomic configurations [47]. First-principles descriptors have also been incorporated into machine learning models of elasticity and evaluated against experimental measurements [72]. These atomic-scale calculations can improve thermodynamic descriptions and provide physically meaningful descriptors, although their limited system size prevents direct representation of heterogeneous microstructures.

Molecular dynamics introduces atomic motion and can examine diffusion, defect migration, and short-time structural rearrangement. Its predictions depend on the interatomic potential, simulated time scale, and diffusion pathway [53]. Experimental observations are therefore required to determine whether a predicted mechanism is active in the processed alloy. In AlCoCrFeNi powders, molecular dynamics was combined with milling experiments to connect particle deformation and defect accumulation with subsequent alloying [73]. At a larger scale, phase-field modelling uses thermodynamic, kinetic, and interfacial inputs to predict the spatial evolution of segregation, precipitation, and grain morphology. Its application to coherent BCC/B2 microstructures illustrates how calculations can describe the evolution of ordered and disordered regions that is not captured by equilibrium phase fractions alone [74]. The reliability of these predictions depends on whether the mobility data, interfacial parameters, initial structure, and thermal history represent the actual processing conditions.

Machine learning provides a statistical bridge among composition, processing, microstructure, and properties. It has extended the prediction of solid-solution formation beyond simple empirical criteria [75]. Experimental data and machine learning have also been combined to identify Ti–Zr–Nb–Ta alloys with desirable hardness [76]. More broadly, machine learning-guided selection followed by synthesis and characterization has demonstrated that computational screening can substantially reduce the experimental search space [77]. These results do not mean that machine learning can replace physical interpretation. A model may reproduce correlations within its training data while failing when the alloy family, processing route, or measured property lies outside that domain.

These approaches are therefore most effective when integrated into an iterative computational–experimental workflow rather than applied independently. CALPHAD can first identify thermodynamically accessible phases and transformation driving forces, while DFT and molecular dynamics provide atomic-scale information on local chemical preferences, energetics, diffusion, and defect behavior. These thermodynamic and kinetic inputs can subsequently support phase-field simulations of processing-dependent segregation, precipitation, and microstructural evolution, whereas machine learning models can integrate composition, processing parameters, calculated descriptors, and experimental data to rapidly screen candidate alloys and process windows. Experiments then determine whether the predicted phases, local structures, defects, and properties are actually produced under the recorded processing history through diffraction, microscopy, chemical analysis, and mechanical or functional testing. Disagreement between prediction and experiment can be used to revise thermodynamic databases, interatomic potentials, kinetic parameters, descriptors, or model assumptions. Such a closed computational–experimental loop is essential for progressing from predictions based mainly on nominal composition toward prediction of the actual microstructure and properties generated by a specific processing route. CALPHAD databases may be incomplete, atomistic calculations sample limited configurations and time scales, phase-field predictions depend on uncertain kinetic and interfacial parameters, and machine learning models may inherit bias from small or inconsistent datasets. Predictions should therefore be reported with their assumptions and uncertainty and validated against the actual processing history, multiscale microstructure, and measured properties.

## 3. Microstructure Formation by Fabrication Route

### 3.1. Mechanical Alloying

**Figure 4:**
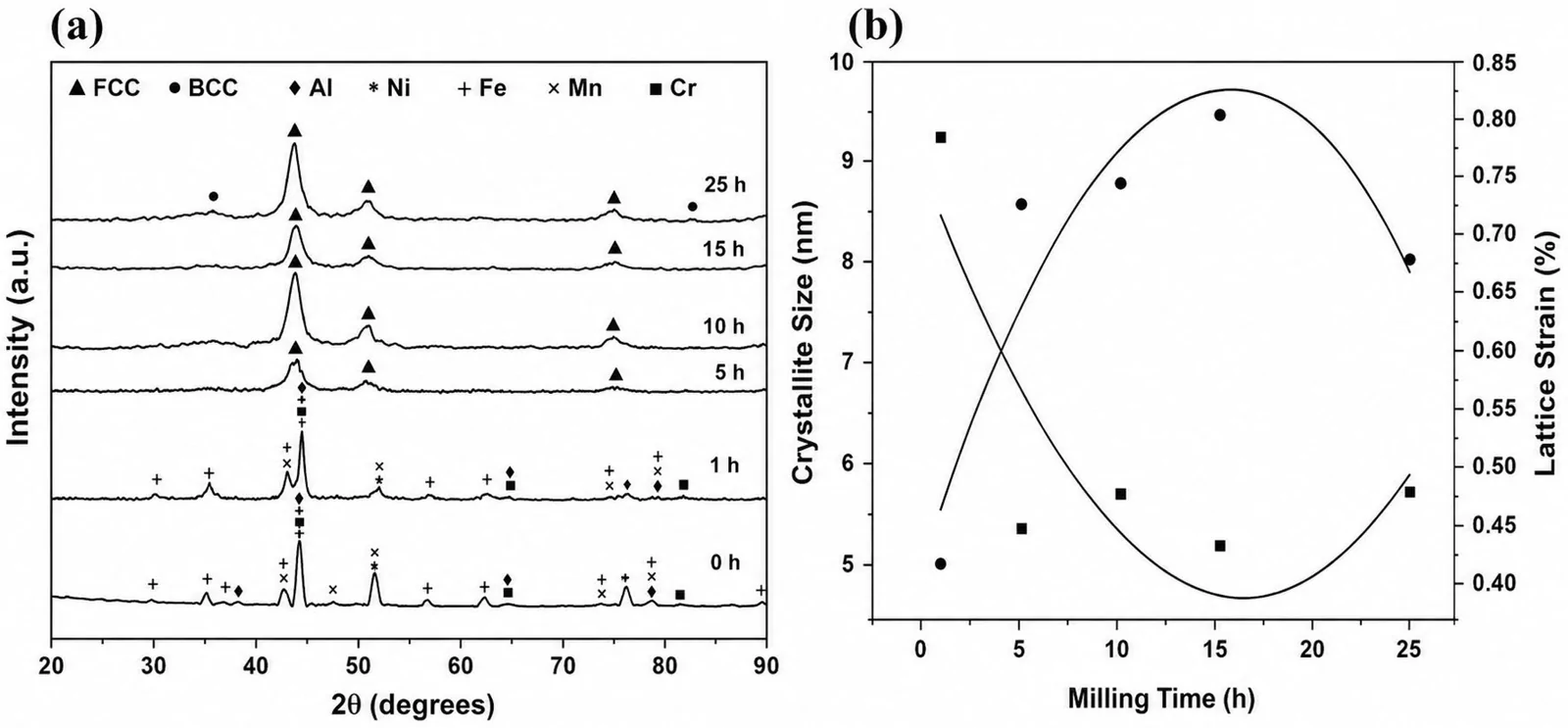
Structural evolution of Fe_34.9_Ni_30.2_Mn_18.6_Cr_9.3_Al_7_ HEA powder with milling time. (**a**) XRD patterns. (**b**) Crystallite size and lattice strain [79].

Mechanical alloying uses high-energy ball milling to subject solid powders to repeated plastic deformation, cold welding, and fracture, thereby shortening diffusion distances and promoting elemental mixing. This method is suitable for multi-principal-element systems with large differences in melting point, a strong tendency to segregate during melting, or a requirement for nanocrystalline and metastable structures. The milled product generally remains a powder. If a bulk material is required, densification must subsequently be performed by spark plasma sintering (SPS), hot pressing, or hot isostatic pressing [78]. Under continuous impact and shear, the powders are first flattened and laminated and then fractured by work hardening. The newly created surfaces subsequently participate in further cold welding. Repetition of this cycle continuously increases the contact area between dissimilar powders, shortens the migration distance required for elemental mixing, and introduces numerous defects such as dislocations and grain boundaries. Combined planetary milling experiments on AlCoCrFeNi and molecular dynamics analysis indicate that particle deformation and defect storage increased the degree of mechanical activation of the powder and further affected alloying during subsequent SPS [73]. The degree of mechanical alloying therefore depends more directly on the actual mechanical energy input and the resulting particle deformation and microstructural evolution than on milling time alone. As shown in Figure 4, diffraction peaks from several initial elements remained visible at the beginning of milling. With increasing milling time, these peaks gradually weakened and an alloyed structure dominated by the FCC phase developed, followed by a small amount of BCC phase at a later stage. Meanwhile, the crystallite size generally decreased from approximately 9 nm to a range of 5 to 6 nm, whereas the lattice strain first increased and then decreased, indicating that defect accumulation, grain refinement, and phase formation did not proceed synchronously. Figure 4 therefore illustrates the different evolution rates of phase structure and microstructure during mechanical alloying and shows that milling time alone cannot accurately determine whether a powder has reached a stable alloyed state.

The introduction of extraneous elements must also be considered during mechanical alloying. Staged chemical analysis of CoCrFeNi powders and sintered bulk materials showed that wear of the milling balls and vial, the process control agent, and subsequent SPS could all cause compositional changes, with C and O contents changing markedly during processing [80]. These extraneous elements not only affect powder composition but may also participate in subsequent reactions in systems containing reactive elements such as Ti, forming carbides or oxides and thereby altering the final phase constitution. Evaluation of powder condition during mechanical alloying must therefore consider possible processing-induced compositional shifts in addition to phase formation and particle refinement.

The powder obtained after milling cannot be taken as a direct representation of the final bulk material. Densification processes such as SPS reintroduce thermal activation, allowing the high-defect structures and metastable phases formed during milling to evolve further. Clear differences were observed between the phase constitution of milled powders and that of sintered high-entropy steels produced by mechanical alloying followed by SPS, indicating that the microstructure formed in the powder continued to change during densification [81]. Further analysis showed that milled Al_0.3_CoCrFeMnNi powder was primarily FCC, whereas SPS produced FCC, B2, M_23_C_6_, and other phases. The added Y_2_O_3_ also transformed during sintering and generated a new nanoscale dispersion [82]. Mechanical alloying therefore primarily determines the initial powder state entering densification, while the final bulk microstructure also depends on phase transformations and elemental redistribution during the subsequent thermal process. The fine grains and high defect density produced by milling do not necessarily disappear completely during subsequent heating. Experimental results showed that nanocrystalline CoCrFeNi prepared by mechanical alloying retained strong resistance to grain growth at a relatively high homologous temperature [83], whereas related work indicated that the final phase structure and microstructure of AlCuNiFeCr were continuously influenced by both mechanical alloying and SPS [84]. A more appropriate analysis therefore treats the milled powder and the densified bulk as two sequentially related but nonequivalent material states: the former determines the degree of elemental mixing and the initial defect structure, while the latter readjusts these features under thermally activated conditions and ultimately forms the microstructure used for property testing.

During mechanical alloying, continuous mechanical energy input promotes powder deformation and solid-state mixing and gradually produces milled powder with a specific phase constitution and defect state. At the same time, the process control agent and milling media may change the composition, while subsequent densification further modifies the microstructure formed during milling. Comparisons among studies must therefore consider the principal milling conditions and distinguish the powder state from the densified material state [85]. Unlike this mechanically driven solid-state mixing route, vacuum melting first produces a liquid alloy, and the final microstructure depends more strongly on retention of melt composition and the subsequent solidification process.

### 3.2. Vacuum Melting

Vacuum melting is a liquid-phase fabrication route in which metallic feedstocks are melted and solidified under vacuum or a high-purity inert atmosphere. Its principal forms are vacuum arc melting and vacuum induction melting. Vacuum arc melting generally uses a water-cooled copper hearth and repeated inversion and remelting to prepare small ingots. Vacuum induction melting uses induction heating and electromagnetic stirring to improve mixing in larger melt volumes [86].

The elements in multi-principal-element systems often differ substantially in melting point, density, and oxygen affinity. Insufficient holding time can leave refractory components or master alloys incompletely dissolved, whereas excessively high temperatures or prolonged holding can aggravate burn-off of volatile elements such as Mn. Appropriate master alloys can reduce volatilization losses to some extent [87]. Reactive elements may also react with residual gases and form inclusions related to the melting atmosphere [88]. Vacuum or a protective atmosphere can therefore reduce the risk of extrinsic reactions, but the actual composition after melting must still be confirmed from mass changes and bulk chemical analysis.

Repeated inversion and remelting can improve macroscopic liquid-state mixing but cannot eliminate solute partitioning during solidification. When the solid–liquid partition coefficient deviates from unity, the dendrites that form first and the residual liquid acquire different compositions. Cooling rate further controls the segregation scale: rapid cooling generally refines dendrites and retains nonequilibrium compositional fluctuations, whereas slower cooling provides more time for elemental redistribution but may also promote second-phase formation and microstructural coarsening [89]. The degree of mixing in the melt and spatial uniformity after solidification should therefore be evaluated separately [90].

**Figure 5:**
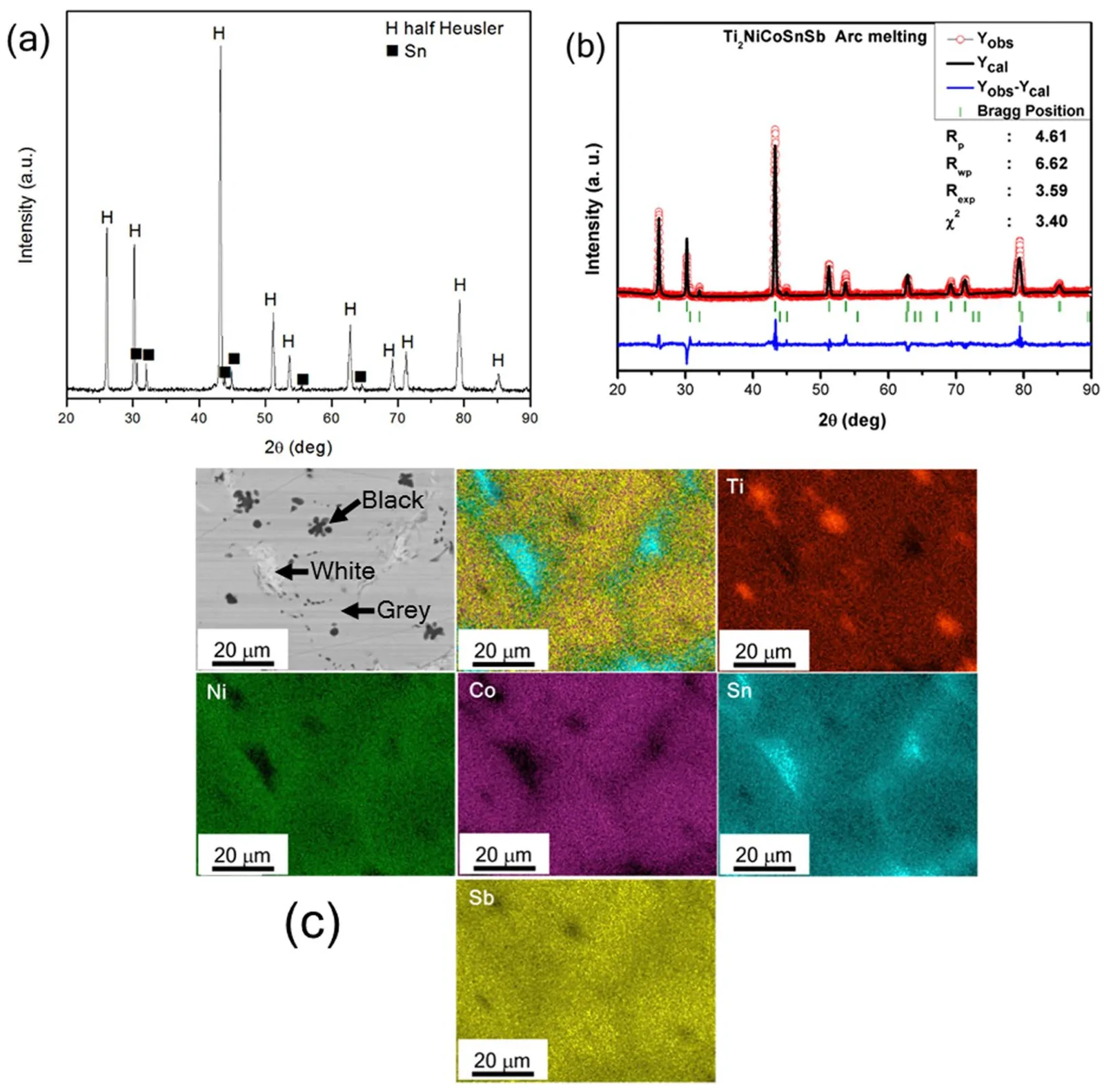
As-cast phase structure and elemental distribution of vacuum arc-melted Ti_2_NiCoSnSb multi-principal-element alloy: (**a**) XRD pattern showing the half-Heusler (HH) matrix phase and a small amount of residual Sn, (**b**) Rietveld refinement confirming the HH phase structure, and (**c**) BSE image and EDS elemental maps showing the gray HH matrix region, white Sn-rich regions, and black Ti-rich regions [7].

Pronounced phase and elemental separation was observed in vacuum arc-melted Hf_25_Zr_30_Ti_20_Nb_15_V_10_, showing that the presence of high-melting-point components does not ensure chemical homogeneity after solidification [91]. Dendritic and interdendritic segregation persisted in a CoCrFeNi ingot after five remelting cycles, further demonstrating that repeated remelting improves macroscopic mixing but cannot remove solute partitioning generated during the final solidification step [92]. The Ti_2_NiCoSnSb half-Heusler multi-principal-element alloy studied by Karati et al. further illustrates that phase constitution and spatial compositional uniformity after solidification are not equivalent [7]. As shown in Figure 5, vacuum arc melting produced a structure dominated by the half-Heusler phase, but XRD still detected approximately 8% residual Sn, while BSE/EDS revealed local Sn-rich and Ti-rich regions. XRD and Rietveld refinement represent the bulk-average phase constitution, whereas microstructural and elemental-distribution analyses reveal local compositional differences. Formation of the target principal phase therefore does not mean that an ingot is chemically homogeneous, and local elemental distributions must also be considered when evaluating vacuum-melted alloys. For larger ingots, sampling at multiple positions is also required to determine whether local compositional differences are representative.

The as-cast phase structure can continue to evolve during subsequent heat treatment. A comparative study examined AlCrTiVNb_x_ alloys with x values of 0.3, 0.4, and 0.5. The as-cast alloys were predominantly single-phase BCC. After annealing at 1000 °C for 2 h, the Nb_0.3_ alloy developed an additional HCP phase, while both HCP and FCC phases appeared in the Nb_0.4_ and Nb_0.5_ alloys. The hardness of the Nb_0.5_ alloy increased from 543 to 725 HV [93]. These results indicate that an apparently single-phase as-cast structure may represent a metastable state retained during solidification. Subsequent annealing can promote elemental redistribution and phase transformation, thereby altering the final microstructure and properties. The heat-treatment schedule also influences the morphology and connectivity of secondary phases. Processing produced a fine-scale coherent BCC/B2 microstructure in AlMo_0.5_NbTa_0.5_TiZr after hot isostatic pressing and high-temperature annealing, and the alloy retained a compressive yield strength of approximately 745 MPa at 1000 °C [94]. A more pronounced effect of phase connectivity was observed in Al_0.5_NbTa_0.8_Ti_1.5_V_0.2_Zr. Related work showed that a continuous B2 phase produced a room-temperature yield strength of 2032 MPa but limited the compressive fracture strain to 4.7%. Subsequent heat treatment transformed the continuous B2 phase into discrete precipitates within a BCC matrix. This reduced the yield strength to 1345 MPa while increasing the compressive strain at first cracking to 38% [95]. These results demonstrate that heat treatment can regulate mechanical performance not only through changes in phase fraction but also by modifying the morphology and connectivity of secondary phases.

To improve the reproducibility of vacuum-melting results, studies should fully report feedstock purity, charging sequence, vacuum level or protective atmosphere, mass changes before and after melting, measured bulk composition, number of remelting cycles, ingot dimensions, and cooling conditions. Burn-off should be corrected in systems containing volatile elements, whereas unmelted particles and local enrichment must be excluded in systems containing high-melting-point components. Phase-constitution and elemental-distribution analyses should cover multiple positions and batches. A single-point EDS measurement or one remelting operation is insufficient to establish compositional uniformity or process stability. Overall, vacuum melting can directly produce bulk HEAs, but the as-cast microstructure is determined jointly by liquid-state mixing and solute partitioning during solidification. Repeated remelting mainly improves macroscopic compositional uniformity and cannot fully eliminate segregation generated during the final solidification step. Subsequent heat treatment promotes elemental redistribution but may also cause phase transformations, precipitation, and microstructural coarsening. Vacuum-melting processes should therefore be evaluated by comparing the measured composition, as-cast segregation, and changes in phase structure before and after heat treatment.

### 3.3. Severe Plastic Deformation

Severe plastic deformation routes, particularly high-pressure torsion and equal-channel angular pressing, impose large shear strains on a pre-existing material state without melting and resolidification [70]. Grain refinement is accompanied by dislocation accumulation, mechanical twinning, grain-boundary formation, and texture development [96]. These structural changes are mutually coupled rather than independent effects. Chemical homogenization is not intrinsic to severe plastic deformation, so inherited segregation or secondary phases may remain after substantial grain refinement. The resulting microstructure is therefore determined by the initial state and deformation path rather than by grain size alone.

For equiatomic CoCrFeMnNi, high-pressure torsion produced an approximately 50 nm FCC grain size, a strength of 1950 MPa, and a hardness of approximately 520 HV. Subsequent annealing caused nanoscale decomposition and a pronounced increase in hardness [97]. This behavior shows that the defect-rich nanocrystalline state can be mechanically strong but thermally unstable. Grain boundaries restrict dislocation motion during deformation, but they can also accelerate elemental redistribution during thermal exposure. Equal-channel angular pressing produced a somewhat coarser ultrafine-grained state with a strength near 1 GPa and an elongation near 35% [98]. Together, these results show that the balance between strength, ductility, and phase stability depends on how strain and thermal history are introduced rather than on refinement alone.

Spatial uniformity is a further limitation. During high-pressure torsion, the increasing strain from the disk center toward the periphery produces corresponding gradients in defect density and hardness [96]. Deformation temperature can also change the dominant deformation mechanism, phase constitution, texture, and final strength [99]. A nominal pressure, rotation number, or pass number therefore cannot represent the complete material state when strain and temperature vary across the processed volume. Comparisons among studies should account for the initial microstructure, deformation path, processing temperature, and sampling position.

Reproducibility can be improved by controlling the starting microstructure and specimen geometry, fully reporting the deformation conditions, and measuring representative radial and through-thickness positions. Grain size, texture, phase fractions, residual stress, and hardness should also be compared among independent specimens. Claims of deformation-induced local chemical order require particular caution because hardness changes and diffraction-peak broadening cannot distinguish atomic ordering from defects, internal strain, or nanoscale phase separation. Reliable identification requires atomically resolved structural evidence combined with chemically sensitive or three-dimensional compositional analysis [15,42]. Severe plastic deformation should therefore be evaluated as a route that generates a path-dependent material state rather than solely as a grain-refinement method.

### 3.4. Magnetron Sputtering

Magnetron sputtering uses magnetically confined plasma to efficiently sputter atoms or molecules from an HEA target onto a substrate under high vacuum. Parameters such as target composition and sputtering power can be adjusted to control the composition and microstructure of the film and thereby obtain relatively uniform HEA films with the desired properties. HEA films can be deposited from a single alloy target or compositionally tuned by co-sputtering multiple elemental or alloy targets, making the method suitable for producing nanocrystalline, amorphous, and compositionally graded structures [8,100]. Unlike bulk melting, magnetron sputtering does not involve bulk liquefaction and solidification. The microstructure is governed mainly by compositional transfer and deposition kinetics.

The nominal target composition does not necessarily match the deposited film composition. Differences in sputtering yield and gas-phase transport among elements can cause compositional deviations during deposition. AlCoCrCuFeNi films deposited from pressed powder targets had an overall composition close to that of the target, although some elements still deviated by several percentage points [101]. Film composition should therefore be measured after deposition rather than represented solely by the target ratio. Magnetron sputtering is characterized by strong nonequilibrium growth. Deposited atoms may be covered by subsequent layers before they can diffuse sufficiently and form equilibrium secondary phases, thereby retaining metastable solid solutions or fine-scale microstructures. The CrCoCuFeNi films consisted primarily of a nanocrystalline FCC solid solution [102], demonstrating that film phase constitution depends not only on chemical composition but also on the conditions for atomic migration and rearrangement. The film phase state therefore cannot be inferred directly from the equilibrium phase state of a bulk alloy with the same composition. Nonequilibrium deposition does not, however, guarantee formation of a single solid solution. Deposition experiments showed that changing the substrate temperature and bias produced ccp, bcc, σ, and χ structures in CrMnFeCoNi films, indicating that film-phase formation depends on atomic migration and rearrangement at the deposition temperature [103]. Together with the nanocrystalline FCC films described above, this result shows that magnetron sputtering expands the range of accessible microstructural states, while the specific phase structure remains controlled by the actual deposition conditions.

**Figure 6:**
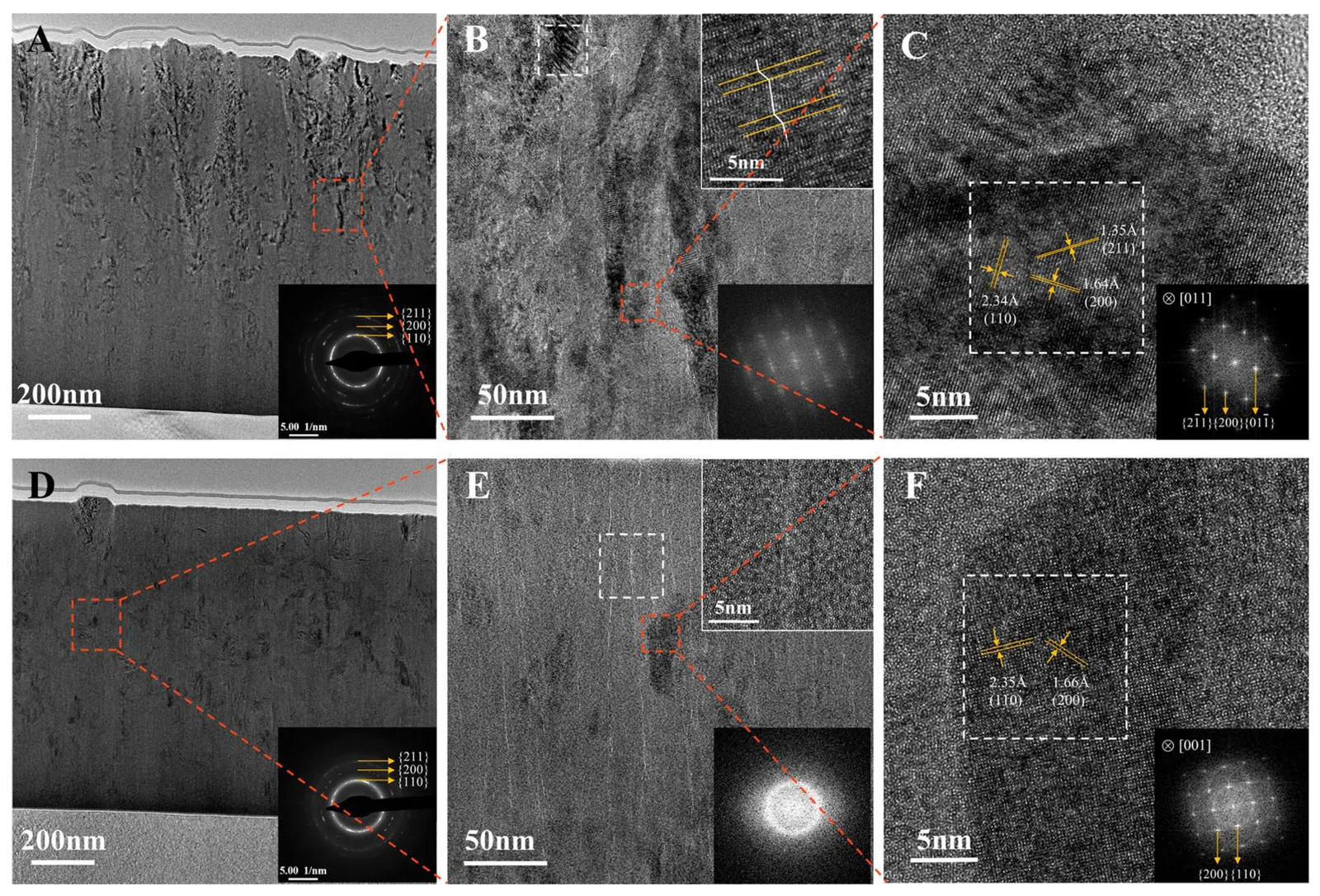
Cross-sectional nanostructures of ZrNbTiMo refractory HEA films deposited at different sputtering powers: (**A**–**C**) 150 W and (**D**–**F**) 210 W, (**A**,**D**) cross-sectional TEM and SAED, (**B**,**E**) locally enlarged TEM images, and (**C**,**F**) HRTEM images and FFT patterns [106].

Working pressure, target power, substrate temperature, and bias are the principal parameters controlling film growth. By changing the flux and energy of incident particles and their surface mobility, these parameters affect grain size and film density [104,105]. Because the parameters interact, the effect of a single parameter on microstructure is generally not a simple linear relationship. The influence of sputtering power on the crystallization state of ZrNbTiMo films further illustrates this coupling. Liu et al. found that films deposited at different powers contained both nanocrystalline and amorphous phases, but their relative fractions changed markedly with power [106]. As shown in Figure 6, the sample deposited at 150 W contained numerous ordered regions larger than 30 nm, whereas the amorphous fraction increased markedly at 210 W, leaving only a few locally ordered regions of approximately 10 to 20 nm. The crystallinity decreased from 20.07% at 150 W to 2.40% at 210 W. The authors proposed that increasing the power from a low level to 150 W increased the energy of incident atoms, favoring surface migration and nanocrystal growth. Further increasing the power to 210 W raised the deposition rate and shortened the time available for deposited atoms to rearrange before being covered by subsequent atoms, thereby suppressing crystallization. The effect of sputtering power on film crystallinity is consequently nonmonotonic and depends on competition between incident-particle energy and the effective time available for atomic rearrangement. Multi-target co-sputtering can also generate variations in film thickness and composition at different substrate positions. Studies of CoCrFeNi films showed that local elemental flux varied with substrate position, producing spatial fluctuations in film composition and thickness [107]. Composition, microstructure, and property measurements should therefore correspond to the same region whenever possible. For high-temperature applications, changes in the phase structure of the as-deposited film during heat treatment or service must also be evaluated [108].

Magnetron sputtering can produce a variety of nonequilibrium microstructures, including nanocrystalline and amorphous structures, by controlling the composition supply and deposition conditions, providing substantial processing flexibility for tailoring HEA films. The final film state, however, is not determined by the nominal target composition or any single parameter. It is jointly controlled by compositional transfer and deposition kinetics. The former determines the actual elemental composition of the film, while the latter affects migration and rearrangement of deposited atoms and thereby regulates grain size, phase structure, and the degree of amorphization. Because processing parameters are coupled, similar compositions can still develop markedly different microstructural states under different deposition conditions. Comparisons among studies should therefore include complete reporting of the principal deposition conditions and characterization of the actual film composition and phase structure. For films intended for high-temperature or long-term service, the thermal stability of the as-deposited nonequilibrium microstructure should also be evaluated to establish the relationships among fabrication conditions, microstructural state, and in-service performance.

### 3.5. Additive Manufacturing

Additive manufacturing (AM) builds materials layer by layer from a digital model and enables near-net-shape production of complex three-dimensional components. The main fusion-based processes used for HEAs are laser powder bed fusion (LPBF), electron beam powder bed fusion (EB-PBF), and directed energy deposition (DED). Laser directed energy deposition (L-DED) and laser engineered net shaping (LENS) are both forms of DED. Powder-bed processes offer relatively high forming accuracy, whereas DED is better suited to high-rate deposition and localized repair. Different manufacturing routes generate different melt-pool scales and thermal histories, so alloys of the same composition may develop different grain structures and solidification substructures. Both L-DED and LPBF produce heterogeneous microstructures containing columnar grains and dislocation networks, but their specific microstructural features and mechanical responses differ markedly [109]. Processing conditions in DED, LPBF, and EB-PBF directly affect the phase structure, microstructural scale, and forming defects of HEAs [110]. Rapid solidification during AM also restricts extensive elemental redistribution and changes phase selection during solidification, producing clear differences between additively manufactured and conventionally cast microstructures [111]. AM of HEAs therefore does more than shape a component: local melting, rapid solidification, and layer-by-layer thermal cycling continuously participate in microstructure formation and ultimately determine the actual material state.

A stable processing window is a prerequisite for high-quality components. Insufficient energy input readily produces lack-of-fusion pores and residual particles, whereas excessive input can cause pores, melt-pool instability, and elemental volatilization. When elemental powder blends are used for in situ alloying, differences in melting point and laser absorptivity among the powders also affect the degree of melting [112,113]. Volumetric energy density is therefore suitable for initial process screening but cannot replace a specific analysis of feedstock condition and the combination of power and scanning speed [114]. In situ alloying of TiZrNbTa shows that an appropriate processing window can improve both build quality and elemental mixing. By optimizing LPBF, Mooraj et al. limited the area fractions of both pores and unmelted particles to below 1% and obtained a yield strength slightly higher than that of the cast sample [34]. Optimization of AM parameters is thus not simply a matter of increasing energy input but of balancing complete melting against melt-pool stability.

**Figure 7:**
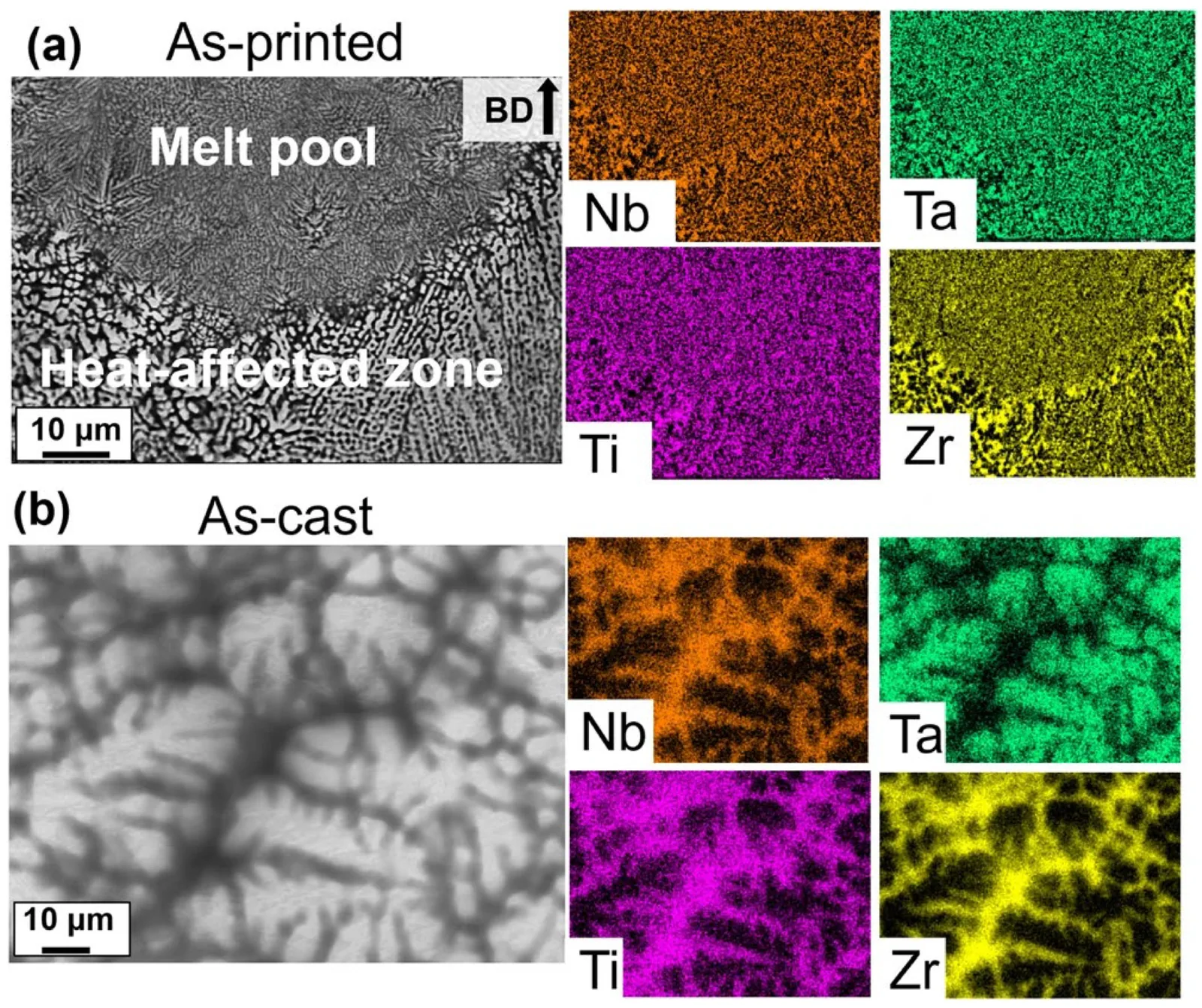
Comparison of elemental distributions in additively manufactured and cast TiZrNbTa refractory HEAs: (**a**) SEM image and elemental maps of an LPBF in situ alloyed sample, showing the melt-pool interior and heat-affected zone; (**b**) SEM image and elemental maps of a vacuum-cast sample at the same scale [34].

Once stable processing has been achieved, rapid solidification further determines the scale and spatial distribution of the additively manufactured microstructure. It generally refines grains and solidification substructures and reduces the scale of elemental segregation, but it cannot completely eliminate solute partitioning during solidification. A transition was observed from columnar grains near melt-pool boundaries to equiaxed grains in the interior of CoCrFeNiMn, showing that local heat flow causes pronounced position-dependent microstructural differences [115]. LENS-fabricated CrMnFeCoNi contained columnar grains, solidification substructures, and a high density of dislocations, further demonstrating the multiscale nature of additively manufactured microstructures [116]. The influence of rapid solidification on elemental distribution is particularly clear in TiZrNbTa. As shown in Figure 7, elemental distribution within the melt pools of the additively manufactured sample was generally uniform, whereas fine-scale compositional fluctuations remained in the heat-affected zone. The vacuum-cast sample, by contrast, exhibited coarser dendritic segregation [34]. Rapid solidification therefore primarily refines the segregation scale rather than producing complete chemical homogeneity at every position. Conventional EDS can identify elemental-distribution differences at larger scales, but finer local segregation requires further confirmation by STEM-EDS or APT. Repeated thermal cycling during layer-by-layer deposition can also continue to alter the solidified microstructure [117]. In EB-PBF AlCoCrFeNi, the lower portion of a component experienced longer high-temperature exposure and reached an FCC phase fraction of approximately 30%, whereas almost no FCC phase was detected at the top. B2 coarsening and elemental redistribution occurred concurrently [118]. Different cumulative thermal histories within the same component can therefore produce pronounced differences in phase constitution, and the microstructural state at one position cannot represent the entire component. Directional heat extraction during fusion-based AM also controls grain selection and crystallographic texture. Epitaxial growth from the preceding layer commonly produces columnar grains aligned with the local heat-flow direction. Scan rotation, melt-pool overlap, remelting, and the balance between thermal gradient and interface velocity can change the preferred orientation or promote a transition toward equiaxed grains. Different scan strategies consequently alter crack paths, grain morphology, and mechanical anisotropy in CoCrFeMnNi [119]. Texture should therefore be measured at several build heights and loading orientations rather than inferred from a single section.

The local thermal history also alters the phase-transformation pathway and cracking susceptibility. Mooraj et al. compared the LPBF and L-DED microstructures of AlCrFe_2_Ni_2_ and found severe solid-state cracking in the single BCC/B2 microstructure formed by LPBF, whereas the slower cooling in L-DED promoted FCC-phase formation. When the L-DED scanning speed was reduced from 6.2 to 3.3 mm/s, tensile elongation increased from 2.2% to 10.3%, while the yield strength decreased from 1120 to 960 MPa [120]. Crack control therefore depends not only on total energy input. Local strain compatibility can also be improved by regulating thermal history and phase fraction. Post-processing can reduce internal defects but also reconstruct substructures generated by rapid solidification. Ron et al. fabricated WTaMoNbV using a laser power of 244 W and a scanning speed of 300 mm/s, followed by hot isostatic pressing. The ultimate compressive strength increased from 1094 to 1220 MPa after treatment, while internal cracking was markedly reduced [121]. The effects of post-processing should therefore be evaluated from both defect closure and microstructural changes rather than density alone. Steep thermal gradients and constrained contraction generate residual stresses that vary with build height, geometry, scan strategy, and the evolving substrate temperature. Neutron diffraction of laser-deposited CoCrFeMnNi showed that the residual-stress distribution followed the thermal gradient and changed along the deposition direction [122]. Residual tensile stress can combine with solidification segregation and limited strain accommodation to initiate hot tearing. In an Al-containing CoCrFeNi alloy, grain-boundary segregation engineering reduced this susceptibility by modifying the terminal solidification path [123]. Preheating, scan-path optimization, composition control, stress-relief treatment, and hot isostatic pressing can reduce residual stress or close defects, but each treatment may also change phase fractions, local order, and texture. Their effectiveness must therefore be verified by simultaneous defect, stress, and microstructure measurements.

Overall, the final material state of additively manufactured HEAs is jointly determined by the forming process and local thermal history. Therefore, evaluation should extend beyond locally optimized properties to include microstructural consistency across different component positions and structural stability after post-processing or thermal exposure. The dependence of material states on processing history highlights the importance of reproducible fabrication strategies and comprehensive validation of microstructural uniformity.

### 3.6. Fabrication Reproducibility and Mitigation Strategies

Fabrication reproducibility in HEAs and MPEAs should be defined as the ability to reproduce a target material state within specified tolerances across different positions, specimens, and independent batches. It includes three linked levels. Compositional reproducibility requires the actual bulk and local compositions to remain within an acceptable range. Microstructural reproducibility concerns grain size, texture, phase fraction, elemental distribution, defect population, and local chemical order. Property reproducibility requires both the mean value and data scatter to remain stable under the same testing conditions. A nominally identical composition or a fixed set of processing parameters does not demonstrate reproducibility unless the resulting material state is also measured and compared.

Elemental segregation and composition fluctuation originate through different mechanisms in different fabrication routes. During casting, solute partitioning in the final solidification step produces dendritic and interdendritic differences, while incomplete dissolution and loss of volatile elements can shift the bulk composition. Powder-metallurgy routes avoid solidification segregation but remain sensitive to powder-batch variation, surface oxidation, milling-media wear, and incomplete particle mixing. In sputtered films, differences in sputtering yield and spatial deposition flux can cause the film composition to deviate from both the target composition and the average value measured at a single position. Additive manufacturing combines melt-pool partitioning with element loss, incomplete melting, and position-dependent remelting. Consequently, compositional reproducibility should be evaluated using measured bulk composition and multi-position chemical analysis rather than nominal feedstock or target composition alone.

Porosity, residual stress, and thermal history constitute a second group of coupled reproducibility factors. Residual pores in powder-metallurgy products depend on particle packing, surface condition, consolidation temperature, pressure, and particle bonding. Additively manufactured components may contain lack-of-fusion pores, gas pores, keyhole pores, unmelted particles, and cracks, with their relative importance changing across the processing window. Residual stress can develop during severe plastic deformation, thin-film deposition, and additive manufacturing because deformation or thermal contraction is spatially constrained. Thermal history further determines whether segregation is retained, metastable phases decompose, ordered structures develop, or grains and precipitates coarsen. Even when nominal parameters remain unchanged, component geometry, sampling position, heat accumulation, equipment condition, and post-processing can produce different local time–temperature histories. These factors interact because porosity and residual stress may promote cracking, while segregation and phase heterogeneity may reduce local strain compatibility.

Improvement should begin with control of the starting materials and continue through processing and final verification. Feedstocks and charges should be characterized for actual composition, particle-size distribution, relevant impurities, and surface condition. Storage atmosphere, charging sequence, vacuum level, protective gas, component geometry, and complete processing parameters should be reported. The coupled effects of current, wire-feed speed, and travel speed on deposition geometry and microstructure in wire-arc processing demonstrate why a single energy parameter cannot establish reproducibility [124]. Where possible, in-process monitoring should record melt-pool behavior, temperature evolution, deposition stability, or consolidation conditions. Process-control strategies should also consider their effects on the final microstructure. Homogenization may reduce segregation but can promote precipitation or grain coarsening. Hot isostatic pressing can close internal pores while altering phase fractions and residual stress. Stress-relief treatment may reduce cracking susceptibility but can also change ordering and nanoscale decomposition. Post-processing should therefore be treated as part of the complete processing history rather than as an independent defect-removal step.

Reproducibility should ultimately be evaluated using predefined structural and statistical criteria. At a minimum, independent batches or builds should be compared in terms of actual composition, segregation amplitude, porosity or crack density, grain size, texture, phase constitution, residual stress, and the relevant property distribution. Measurements should cover representative positions because a single section may not capture radial, through-thickness, build-height, or film-thickness gradients. Property data should be reported using the mean value, scatter, specimen number, sampling position, and test condition rather than only the best-performing specimen. Table 3 summarizes the principal route-dependent sources of microstructural variation, while Table 4 provides quantitative examples showing how changes in deformation, heat treatment, build position, and post-processing produce different material states and properties at the same nominal composition. Together, these comparisons show that apparently conflicting property values for the same nominal composition can arise from differences in local thermal history, sampling position, defect population, and post-processing. Such differences should not be interpreted as intrinsic composition effects until the resulting material states and test conditions are shown to be comparable.

## 4. Microstructure Characterization and Evolution

### 4.1. Phase Constitution and Local Order

Structural characterization of HEAs must first distinguish the average phase constitution from the local atomic arrangement. Laboratory XRD is mainly used to identify fundamental crystal structures such as FCC, BCC, and HCP, while high-energy synchrotron XRD can further quantify phase fractions and lattice parameters. At progressively smaller scales, electron diffraction and atomic-resolution imaging can identify local crystallographic features, whereas atom probe tomography (APT) provides three-dimensional elemental distributions. Because these methods probe different structural scales, identical average lattice structures do not imply identical local atomic occupation or chemical environments [42]. This distinction is particularly important for identifying ordered structures. Observation of the fundamental FCC or BCC reflections alone does not establish that a material is a completely random solid solution. L1_2_ and FCC share the same fundamental lattice, as do B2 and BCC, and ordering must often be identified through weak superlattice reflections. When ordered regions are small or highly coherent with the matrix, these signals can also be affected by peak broadening and background intensity. Moreover, additional reflections in electron diffraction do not necessarily originate from chemical order. Experiments and simulations show that stacking faults and multiple scattering can also generate additional diffraction signals [128]. Weak diffraction features must therefore be validated using different zone axes and other element-sensitive methods. Even when the average crystal structure has been established, the local structure may exhibit pronounced fluctuations. Wang et al. found heterogeneous atomic displacements and local strain within the average structure of the single-phase B2 high-entropy intermetallic (CoNi)_50_(TiZrHf)_50_, as identified by XRD and EBSD [129]. Thus, single phase primarily describes the average crystallographic state and does not directly represent atomic arrangements at smaller scales.

**Figure 8:**
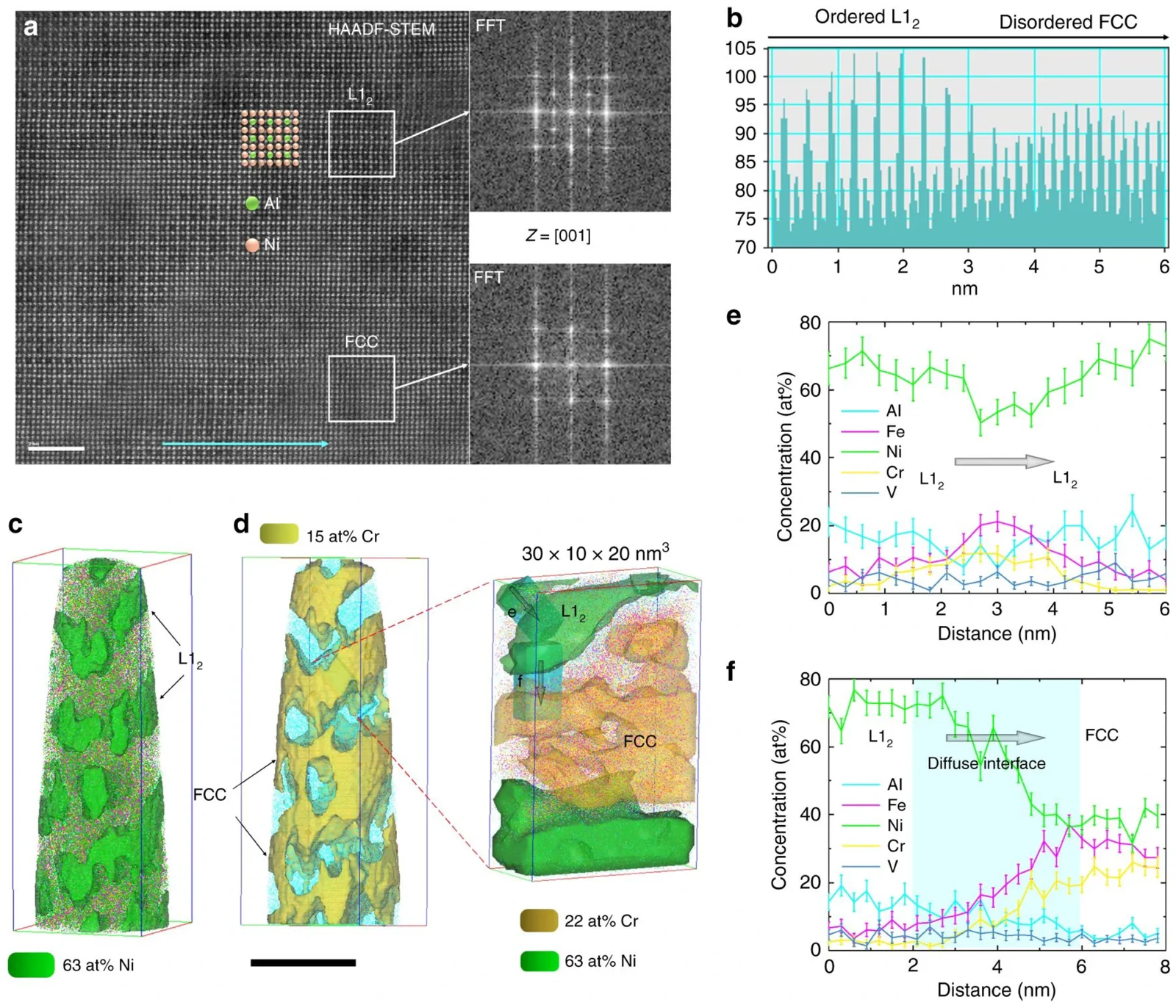
Local structure and three-dimensional elemental distribution in Al_0.5_Cr_0.9_FeNi_2.5_V_0.2_ HEA. (**a**–**c**) HAADF-STEM images and FFT patterns of ordered and disordered regions. (**d**–**f**) APT reconstruction and composition profiles showing elemental partitioning [35].

Identification of chemical short-range order requires more than detecting additional diffraction features because an identical average crystal structure can accommodate markedly different local atomic arrangements. Evidence from CrCoNi shows that samples with similar average FCC structures can nevertheless exhibit different short-range order states after different thermal histories, indicating that average diffraction alone cannot uniquely determine local chemical organization [130]. This limitation becomes even more important when ordered and disordered regions have similar lattice parameters, as in Al_0.5_Cr_0.9_FeNi_2.5_V_0.2_. In this case, the average FCC and L1_2_ phase constitution provides only the crystallographic framework, whereas atomic-resolution HAADF-STEM identifies local ordered occupation and APT reveals the associated three-dimensional elemental partitioning [35]. As shown in Figure 8, the ordered regions exhibit L1_2_-type superlattice features and periodic atomic-column contrast, while the corresponding APT reconstruction shows a gradual transition between Ni-rich ordered regions and Cr-rich disordered regions rather than a sharply separated interface. These observations suggest that local order in HEAs should not be treated as an isolated diffraction signature, but as a structural state that must be established by correlating crystallographic evidence with local chemical information. More importantly, the degree and spatial distribution of ordering may depend strongly on processing history, meaning that the same nominal composition and average phase constitution can represent different local material states. A reliable description of local order therefore requires agreement among bulk diffraction, atomic-scale imaging, and three-dimensional compositional analysis rather than reliance on any single technique.

As the characterization scale decreases further, judgments of homogeneity and order must also account for limitations in the detection scale and analytical method. Multiscale characterization showed that the absence of obvious elemental segregation at larger scales in Fe_49.5_Mn_30_Co_10_Cr_10_C_0.5_ did not exclude the formation of nanoscale Cr-rich carbides during subsequent thermomechanical treatment [36]. Compositional homogeneity at the micrometer scale therefore cannot be extrapolated directly to the nanometer or atomic scale. For scattering data, the structural model itself may also introduce nonuniqueness. Synchrotron scattering, reverse Monte Carlo analysis, and first-principles calculations of local coordination in CoCrFeNi showed that different local-order models could produce similar average scattering features [131]. A single fitting result is therefore insufficient to uniquely determine the local atomic arrangement and must be further constrained by element-sensitive or atomic-scale evidence.

The degree of local order also changes with the material state, so macroscopic properties should not be inferred directly from local structural observations. Bacurau et al. compared ordering in CoCrNi and CrNi_2_ and found that prolonged aging altered the degree of short-range order in CoCrNi but caused relatively limited changes in its overall tensile properties. By contrast, formation of long-range order in CrNi_2_ produced a more pronounced change in strength [132]. Whether local order substantially affects material response therefore also depends on its degree and spatial extent. Heat-treatment studies further linked heat-treatment-induced local chemical order to strengthening, showing that processing history can alter atomic occupation [133]. With advances in characterization, analysis of such local structures is progressing from two-dimensional observation to three-dimensional quantification. Atomic electron tomography revealed heterogeneous distributions of local chemical order and strain [134], whereas Li et al. used machine learning-enhanced APT nearest-neighbor analysis to improve quantitative identification of three-dimensional short-range order [135]. These results remain sensitive to reconstruction methods and statistical criteria, however, so high spatial resolution cannot replace cross-validation among different forms of evidence.

Overall, phase identification in HEAs should not be regarded as a simple extension from bulk diffraction to higher spatial resolution, but as a progressive refinement of structural description across different length scales. Average diffraction establishes the dominant crystallographic framework, whereas local ordering, compositional fluctuations, and coherent nanoscale domains may remain hidden within an apparently single-phase structure. Their identification therefore requires mutually constrained crystallographic and chemical evidence rather than reliance on any individual technique. More importantly, the interpretation of local order is inherently scale- and model-dependent, because similar average diffraction features may arise from different atomic configurations and locally observed ordered regions may not be representative of the bulk material. Reliable structural analysis must consequently distinguish average phase constitution from local atomic organization and evaluate whether the spatial extent, chemical partitioning, and statistical significance of local order are sufficient to influence the material response. Multiscale characterization is therefore most valuable when it converts isolated structural observations into a physically consistent description linking phase constitution, local chemical organization, and processing-dependent structural evolution.

### 4.2. Microstructure and Elemental Distribution

After the average phase constitution and local structure have been established, the next challenge is to determine where specific microstructural features occur and how they are related to local composition and defect state. This spatial correlation is particularly important in HEAs because similar average crystal structures can contain markedly different grain morphologies, secondary-phase distributions, interfacial chemistries, and deformation substructures. A single high-resolution image may reveal local details but cannot establish whether those features are representative of the bulk microstructure. Reliable interpretation therefore requires large-area statistical characterization to be connected with progressively higher-resolution analysis of selected regions. EBSD can define grain orientations, phase distributions, and boundary characteristics over relatively large areas, while EDS or EPMA identifies corresponding compositional variations and ECCI, TEM, or APT resolves finer defect structures and local chemical fluctuations. The value of this approach lies not in increasing spatial resolution alone, but in determining whether a particular defect or compositional feature is systematically associated with a specific grain, phase, or interface. Correlative EBSD and ECCI analysis of additively manufactured FeCoNiCr containing 1.8 at.% N illustrates this principle by linking grain-scale structural information with dislocation substructures and geometrically necessary dislocation distributions [136]. Such correlations allow local deformation behavior to be interpreted within its actual microstructural context rather than as an isolated microscopic observation. Microstructural characterization in HEAs should therefore progress from statistical identification of representative regions to targeted local analysis, so that phase constitution, elemental distribution, and defect state can be connected within the same spatial framework.

**Figure 9:**
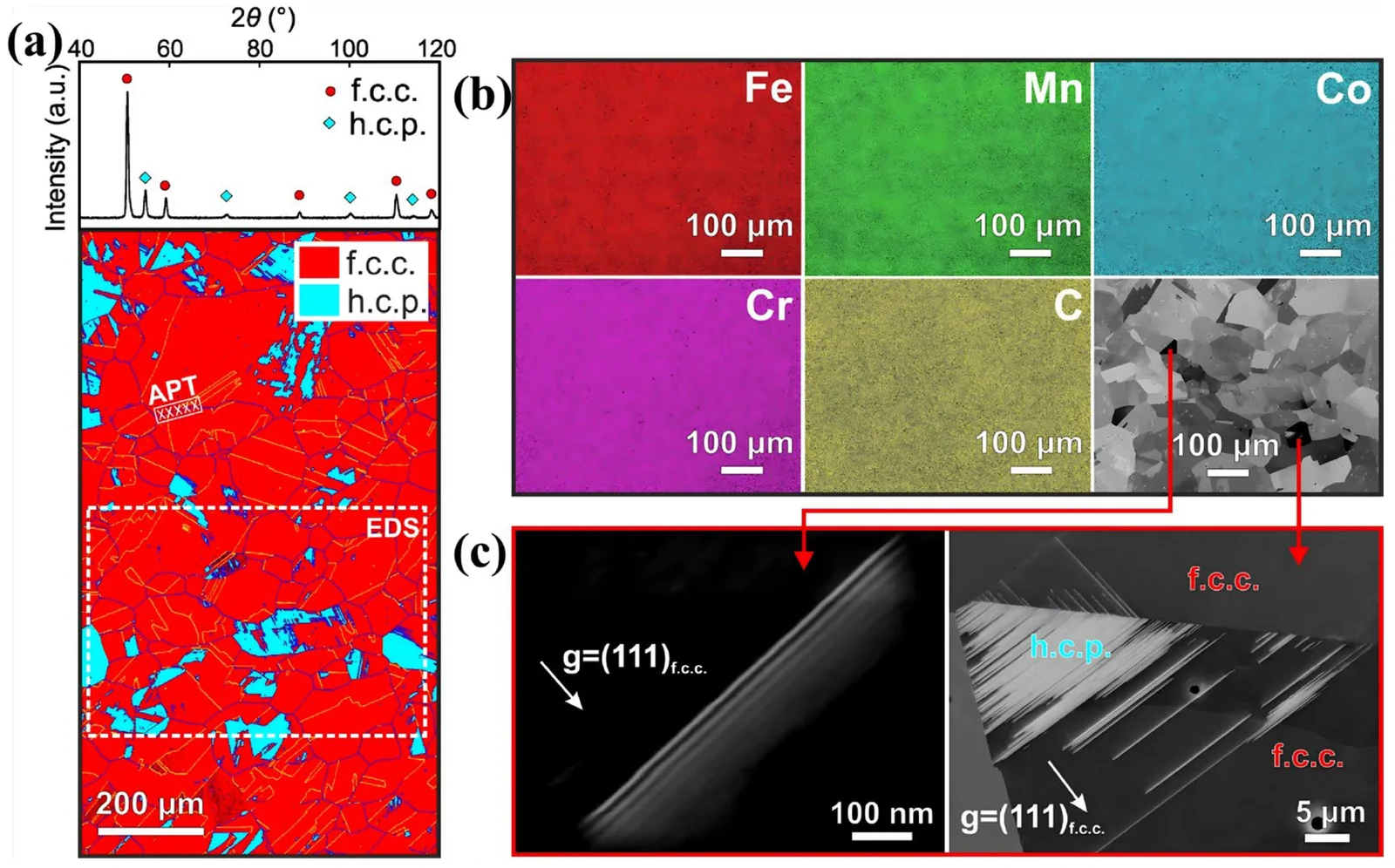
Multiscale characterization of Fe_49.5_Mn_30_Co_10_Cr_10_C_0.5_ HEA. (**a**) XRD and EBSD of FCC and HCP phases. (**b**) BSE image and EDS maps. (**c**) ECCI showing HCP lamellae and stacking faults [36].

Multiscale methods characterized the microstructure of the interstitial HEA Fe_49.5_Mn_30_Co_10_Cr_10_C_0.5_ across multiple scales. In the homogenized state, the alloy consisted mainly of an FCC matrix and lamellar HCP phase. EBSD, EDS, and ECCI were further used to analyze the spatial distributions of the two phases and their relationships with local defects [36]. As shown in Figure 9, XRD first identified the two principal FCC and HCP phases, while EBSD located the HCP lamellae within FCC grains. EDS did not show that the HCP regions corresponded to pronounced segregation of principal elements, whereas ECCI revealed clear stacking-fault structures near the HCP lamellae. Cross-scale correlation indicates that HCP formation in this state was more closely associated with local defect structures and could not be attributed simply to elemental segregation at the micrometer scale. This correspondence also shows that compositional homogeneity at a larger scale does not exclude elemental redistribution at a smaller scale. After subsequent thermomechanical treatment, the grains were markedly refined, the HCP phase was substantially reduced, and nanoscale Cr-rich particles appeared in the matrix. APT further identified these particles as M_23_C_6_-type carbides [36]. Microstructural evolution can therefore be accompanied by local redistribution of C and Cr that may not be fully resolved by conventional EDS. Elemental homogeneity in HEAs must consequently be assessed at the relevant observation scale and in relation to the spatial relationships among phase structure, elemental distribution, and local defects.

Microstructural heterogeneity is not merely a characterization issue but can directly determine where local phase transformations and deformation processes occur. In partially recrystallized Fe_40_Co_20_Cr_20_Mn_10_Ni_10_, the FCC phase preferentially transformed into HCP in unrecrystallized regions with a high dislocation density rather than in recrystallized grains, despite their similar overall chemical composition [137]. This behavior shows that local defect state can modify transformation propensity independently of nominal composition and average phase constitution. Resolving such relationships requires high spatial resolution, but increasing resolution simultaneously reduces the sampled volume and therefore raises the problem of statistical representativeness. In eutectic Al_18_Co_30_Cr_11_Fe_11_Ni_30_, local TEM observations identified semicoherent FCC/BCC interfaces and their crystallographic relationship, while large-area EBSD was required to determine whether these local features were representative of the broader lamellar microstructure [138]. The same limitation becomes more pronounced at still smaller scales, where APT and atomic-scale analyses reveal nanoscale segregation and subnanometer chemical fluctuations even in alloys that appear single phase by XRD [139,140]. These observations indicate that local structural features should not be interpreted independently of their spatial frequency and microstructural context. A meaningful characterization strategy must therefore determine not only what exists at the nanoscale, but also where it occurs, how frequently it appears, and whether it is systematically associated with particular grains, interfaces, defect states, or compositional fluctuations. In this sense, the distinction between average structure and local state is not simply a matter of spatial resolution but is essential for identifying which microstructural heterogeneities actually control phase evolution and material response.

Overall, the significance of microstructural characterization lies in establishing how phases, elemental distributions, interfaces, and defects are spatially coupled within the same material state. These features should not be interpreted independently, because local compositional variations can influence phase stability, while interfaces and defect structures can in turn modify elemental redistribution and subsequent transformation behavior. The objective is therefore to identify which combinations of structural and chemical features consistently accompany a particular microstructural response. Such spatially resolved relationships provide the basis for connecting processing-induced heterogeneity with subsequent property evolution.

### 4.3. Surface Chemical States

After the internal phase structure and elemental distribution have been established, characterization must extend to the surface region that participates directly in environmental reactions. Although the outermost material layer is generally only a few nanometers thick, its chemical composition is not a simple continuation of that of the matrix. During corrosion or oxidation, elements differ in their tendency to participate in interfacial reactions: some components may dissolve preferentially, while others become enriched at the surface and form stable reaction products. The resulting surface film often has a composition and chemical state markedly different from those of the matrix, and the species may be redistributed through the film depth. Understanding HEA surfaces therefore requires more than identifying the oxides that form. It also requires determining the states in which elements are retained after environmental exposure and their spatial distributions within the film.

Al_1.5_TiVCr provides a clear example of such selective surface reactions. Qiu et al. combined online ICP with XPS to analyze anodic polarization and found marked differences in the interfacial behavior of the elements: Al underwent strong preferential dissolution, whereas Ti was largely retained at the surface before breakdown [141]. Accordingly, the composition of the surface oxide measured by XPS deviated markedly from the nominal elemental proportions of the matrix. Even when the initial alloy has a defined bulk composition, the interfacial state after corrosion or passivation is controlled by the surface chemistry produced by environmental exposure rather than by the nominal bulk composition itself. Surface chemical characterization must therefore first determine the selective elemental changes and corresponding chemical states caused by interfacial reactions and then analyze their spatial distributions within the film.

**Figure 10:**
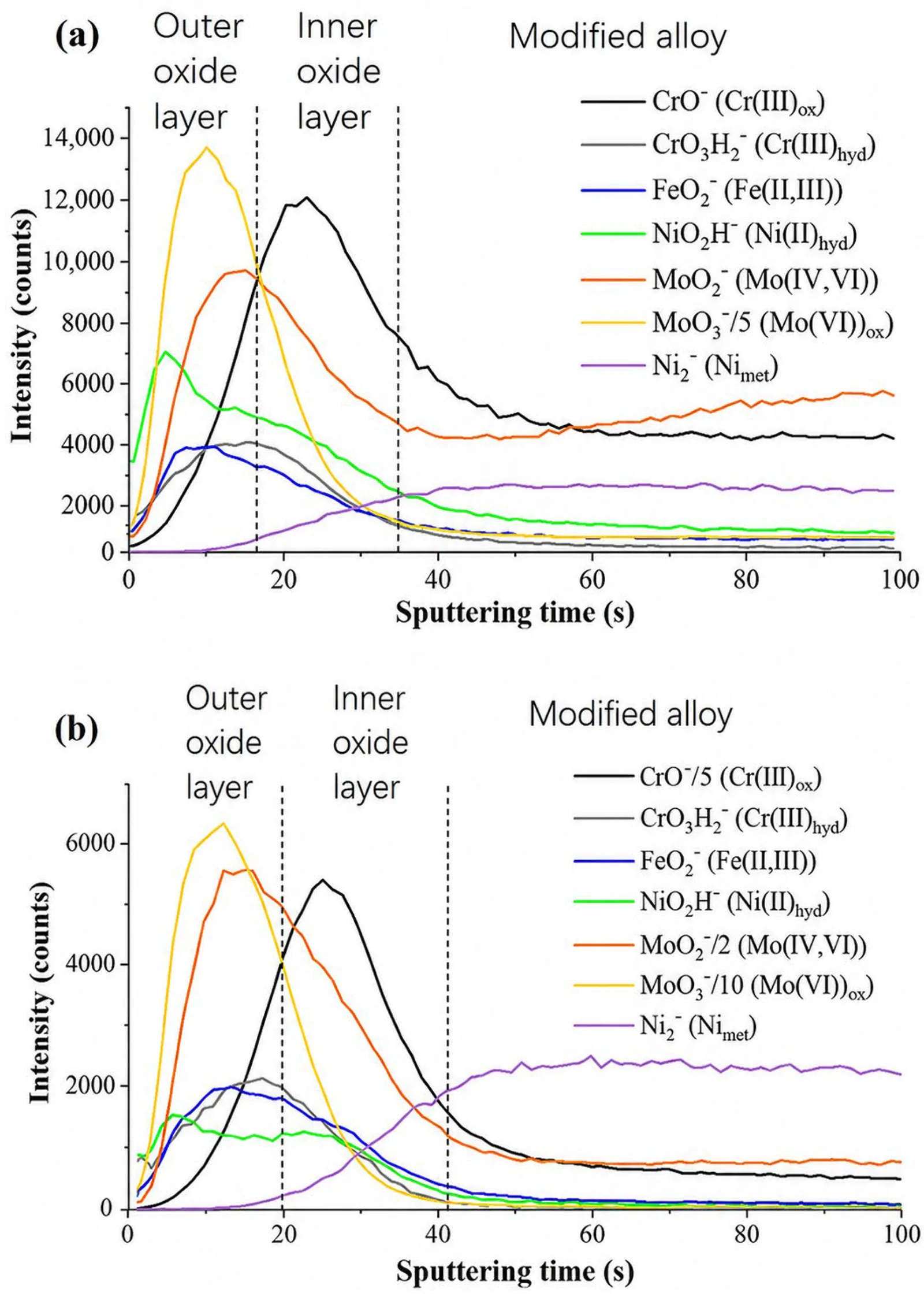
ToF-SIMS depth profiles of surface films on CrFeCoNiMo MPEA. (**a**) Native film and (**b**) anodically passivated film showing chemical stratification across the film depth [142].

After the surface elemental composition has been determined, the spatial distributions of different chemical states within the film must be established. XPS identifies surface elements and their valence states, while depth profiling by ToF-SIMS tracks changes in these species through the film thickness. In the ToF-SIMS depth profiles, the individual curves represent the depth-dependent secondary-ion signals of different oxide species, and their relative positions reflect the chemical stratification of the surface film. Comparison of the ToF-SIMS profiles in Figure 10 shows that anodic passivation broadens the oxide-related region relative to the native film, indicating film growth [142]. The MoO_3_^−^ signal is concentrated nearer the outer surface, whereas MoO_2_^−^ occurs deeper and closer to the CrO^−^-rich inner region. This positioning is mechanistically important because Mo(IV) species near the barrier layer can reinforce locally Cr-depleted sites that are susceptible to chloride attack. Figure 10 therefore shows that enhanced passivity arises from the depth-dependent organization of Mo and Cr species rather than from bulk composition or total oxide content alone. Similar selective partitioning occurs in multiphase HEAs. Different phase regions in CoCrFeMoNi can form surface films with different compositions, accompanied by local enrichment of Cr and Mo [143], while variations in Nb content alter elemental redistribution and oxidation states at the surface of Al_0.5_Ti_3_Zr_0.5_Nb_x_Mo_0.2_ [144]. XPS and ToF-SIMS likewise revealed clear inner- and outer-layer differences in CoCrFeMnNi, showing that selective oxidation and compositional gradients develop during passivation even when the matrix is relatively homogeneous at larger scales [145]. The effects of matrix composition and phase structure on surface stability must ultimately be interpreted through the composition, chemical states, and depth distribution of the resulting film.

Surface films continue to evolve with exposure conditions. After immersion of CrCoNi and CrMnFeCoNi in 1 mol·L^−1^ H_2_SO_4_ for 28 d, the former gradually developed a relatively stable Cr-rich surface layer, whereas the latter formed loose mixed corrosion products [146]. An in situ XPS study of CoCrFeMnNi further showed that several selective oxides were already present during initial oxidation at 100 °C and that the chemical state of the film continued to change as temperature increased [147]. ^18^O isotope tracing also showed that outward cation migration was faster than inward oxygen migration during reoxidation [148]. A film obtained by static analysis therefore represents only the state at a specific time. Its final structure results from continuous elemental migration and selective oxidation.

Surface films on HEAs should be regarded as dynamically selected interfacial states rather than passive extensions of the bulk composition. Selective dissolution, oxidation, and elemental migration continuously redistribute alloying elements across the film and generate depth-dependent chemical states, so nominal composition alone cannot determine whether a protective surface will develop. More importantly, film stability depends on whether this reconstructed chemical architecture can be maintained as exposure conditions evolve. A favorable surface composition observed at a single time may therefore represent only a transient state rather than a stable protection mechanism. Surface characterization should consequently resolve not only which species are present, but also how their valence states, depth distributions, and migration pathways evolve with exposure. This perspective shifts the interpretation of corrosion and oxidation resistance from static film composition toward the formation, persistence, and eventual degradation of the working interfacial state.

### 4.4. In Situ Characterization

Static characterization can identify the phases and defects retained after an experiment but cannot readily establish the order in which they formed. In HEAs, phase transformations and defect evolution commonly proceed continuously with load or temperature, and similar final states may arise through different pathways. The principal purpose of in situ characterization is therefore not merely to obtain dynamic images but to correlate structural changes directly with the external-field history and thereby identify the conditions and sequence of transformation.

**Figure 11:**
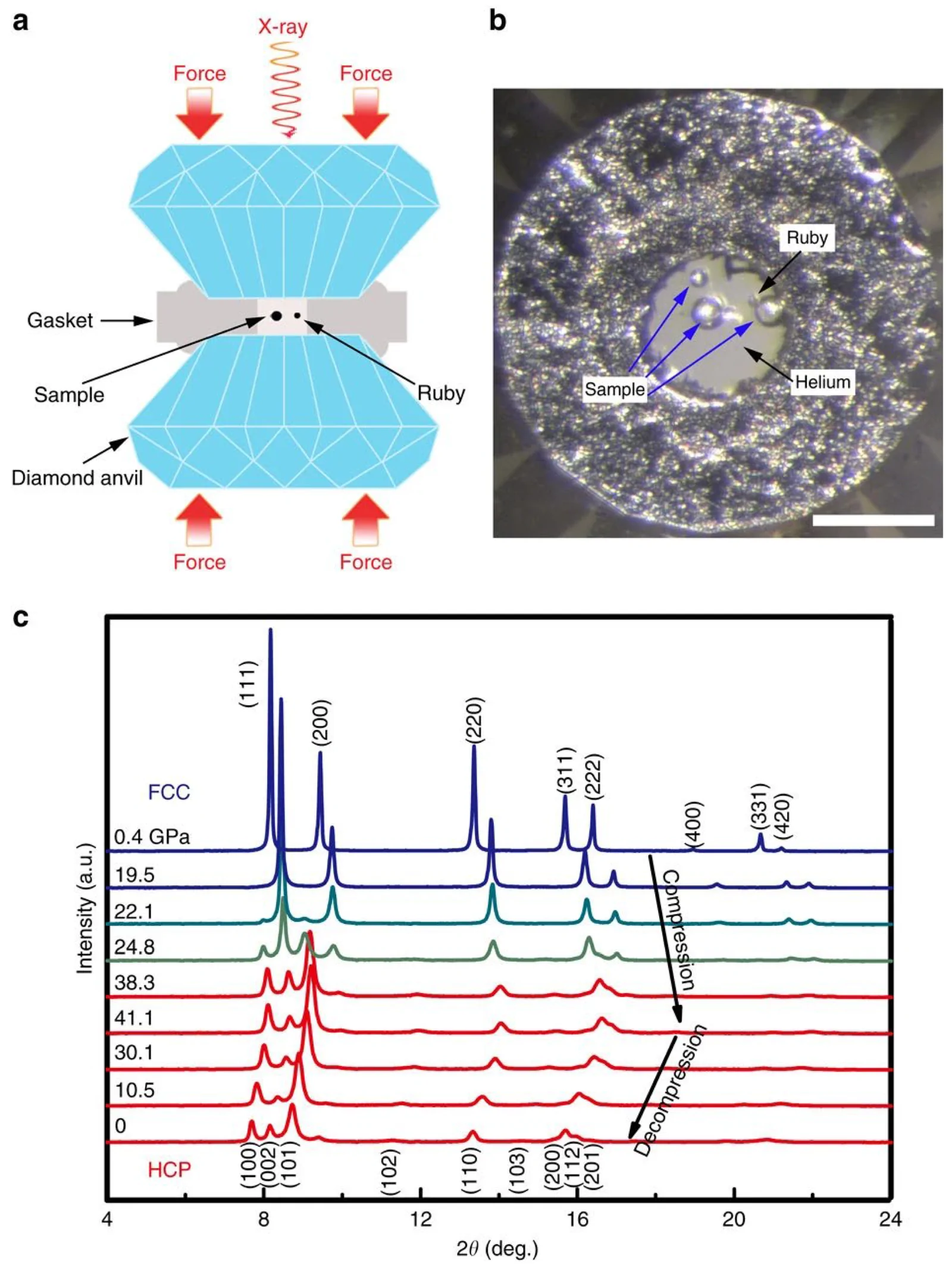
High-pressure in situ synchrotron XRD of CrMnFeCoNi HEA. (**a**) Diamond anvil cell setup. (**b**) Sample chamber. (**c**) XRD evolution during compression and decompression, showing the transformation from FCC to HCP and partial retention after unloading [5].

In situ synchrotron XRD can continuously track phase-structure evolution under an external field over a relatively large sampling volume. Tracy et al. performed a high-pressure in situ study of CrMnFeCoNi and found that the initial FCC structure began to transform to HCP at approximately 14 GPa and continued to evolve during subsequent compression [5]. As shown in Figure 11, the characteristic HCP reflections did not disappear completely after decompression, indicating that part of the pressure-induced structure persisted to low pressure. An FCC phase stable at ambient pressure can therefore enter a new structural state under high pressure, while the final microstructure after unloading does not fully reveal the preceding transformation pathway. Heating, conversely, promotes transformation from HCP to FCC [149], demonstrating that phase stability in this system depends not only on the instantaneous pressure but also on the pressure–temperature path.

**Figure 12:**
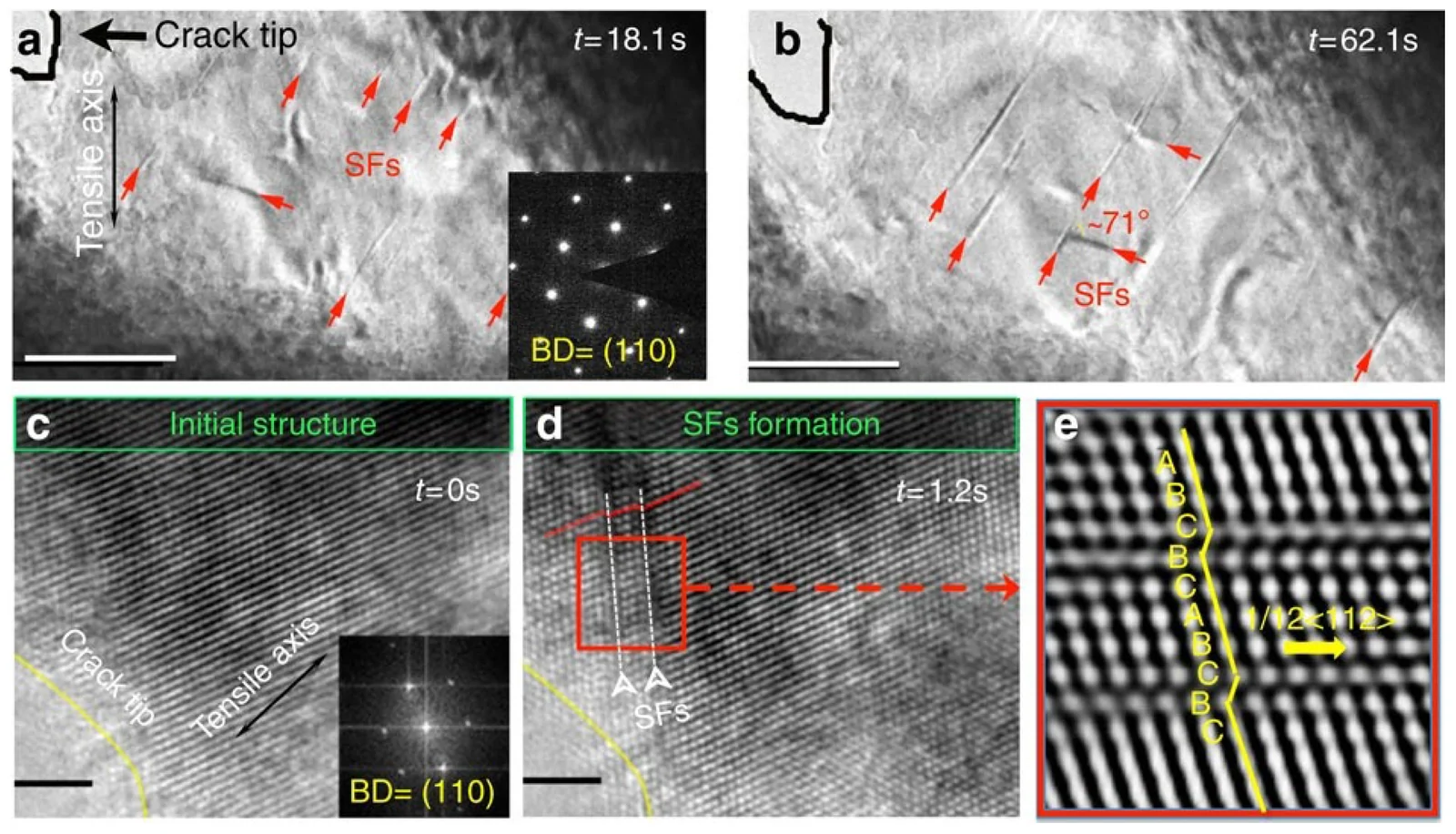
In situ TEM observations of crack-tip plasticity in CrMnFeCoNi. (**a**) Initial formation of stacking faults during loading. (**b**) Interaction of stacking faults. (**c**) Initial crack-tip structure. (**d**) Stacking-fault formation near the crack tip. (**e**) Atomic-resolution image showing local HCP-type stacking [37]. The black arrow labeled ‘Crack tip’ marks the crack tip, red arrows mark stacking faults, and the red dashed arrow links the boxed region in (**d**) to its atomic-resolution enlargement in (**e**).

In situ characterization reveals that deformation and thermal evolution in HEAs are governed not by isolated phase changes, but by the sequential redistribution of load, defects, and local structural instability. In multiphase alloys, the macroscopic stress response originates from unequal yielding and load transfer between constituent phases. For example, in Al_0.6_CoCrFeNi and eutectic AlCoCrFeNi_2.1_, the softer FCC phase yields earlier, whereas the harder BCC-based or B2 phase subsequently carries a larger fraction of the applied load [150,151]. This phase-level partitioning provides the mechanical background for defect activity at smaller scales. In situ TEM further shows that crack-tip deformation in CrMnFeCoNi begins with partial-dislocation emission and stacking-fault formation, followed by interactions among faults and local changes in stacking sequence, while related studies show that extended stacking-fault networks can provide preferred sites for HCP nucleation and interact with twin boundaries during continued deformation [37,152,153]. These observations indicate that phase transformation, defect accumulation, and load redistribution should be interpreted as coupled stages of a single deformation pathway rather than as independent phenomena. A similar path dependence appears during heating. In CrFeCoNiCu and Al_x_CoCrFeNi, progressive temperature increases alter phase fractions and promote intermetallic or σ-phase formation, demonstrating that the final heat-treated structure records only the endpoint of a sequence of intermediate states [154,155]. The main value of in situ methods is therefore not simply that they capture transient structures, but that they identify the order in which phase instability, defect activity, and elemental or structural redistribution develop. This temporal information is essential for distinguishing mechanisms that may lead to similar final microstructures but arise through fundamentally different evolutionary pathways. Representative in situ crack-tip observations are shown in Figure 12.

Interpretation of in situ results must also consider the initial material state. In LPBF Fe_49.5_Mn_30_Co_10_Cr_10_C_0.5_, textures produced along different build directions altered activation of the transformation from FCC to HCP and nanoscale twinning [156]. In situ tensile testing of TiHfZrNb specimens smaller than 100 nm also revealed local elastic strain approaching 10%, followed by lattice disordering [157]. In situ experiments record the dynamic response of a specific specimen state. Their results therefore cannot be generalized directly to all bulk materials without accounting for orientation and size.

In situ characterization is most valuable when it resolves the sequence by which an external field drives phase transformation, load redistribution, and defect evolution, thereby distinguishing mechanisms that may lead to similar post-mortem structures. Because the observed pathway also depends on the initial microstructure, orientation, specimen size, and loading history, local dynamic observations cannot be generalized directly to bulk behavior. Their mechanistic relevance should therefore be evaluated by combining in situ evidence with post-mortem characterization and bulk property measurements. Table 5 summarizes the characteristic scales, measurable structural information, associated property insights, and principal limitations of the multiscale techniques discussed above.

## 5. Properties and Engineering Applications

### 5.1. Strength–Ductility Synergy

Aerospace load-bearing structures require not only high yield strength but also stable plasticity under continued loading and sufficient resistance to initial defects and cyclic loads. Simply increasing strength can cause plastic deformation to localize prematurely if work-hardening capacity decays rapidly, while high tensile elongation without resistance to crack growth cannot ensure the safety of a defect-containing component. The potential of HEAs for aerospace load-bearing structures should therefore be evaluated in terms of uniform deformation capacity, crack-damage tolerance, and cyclic life rather than from a single tensile metric [70].

**Figure 13:**
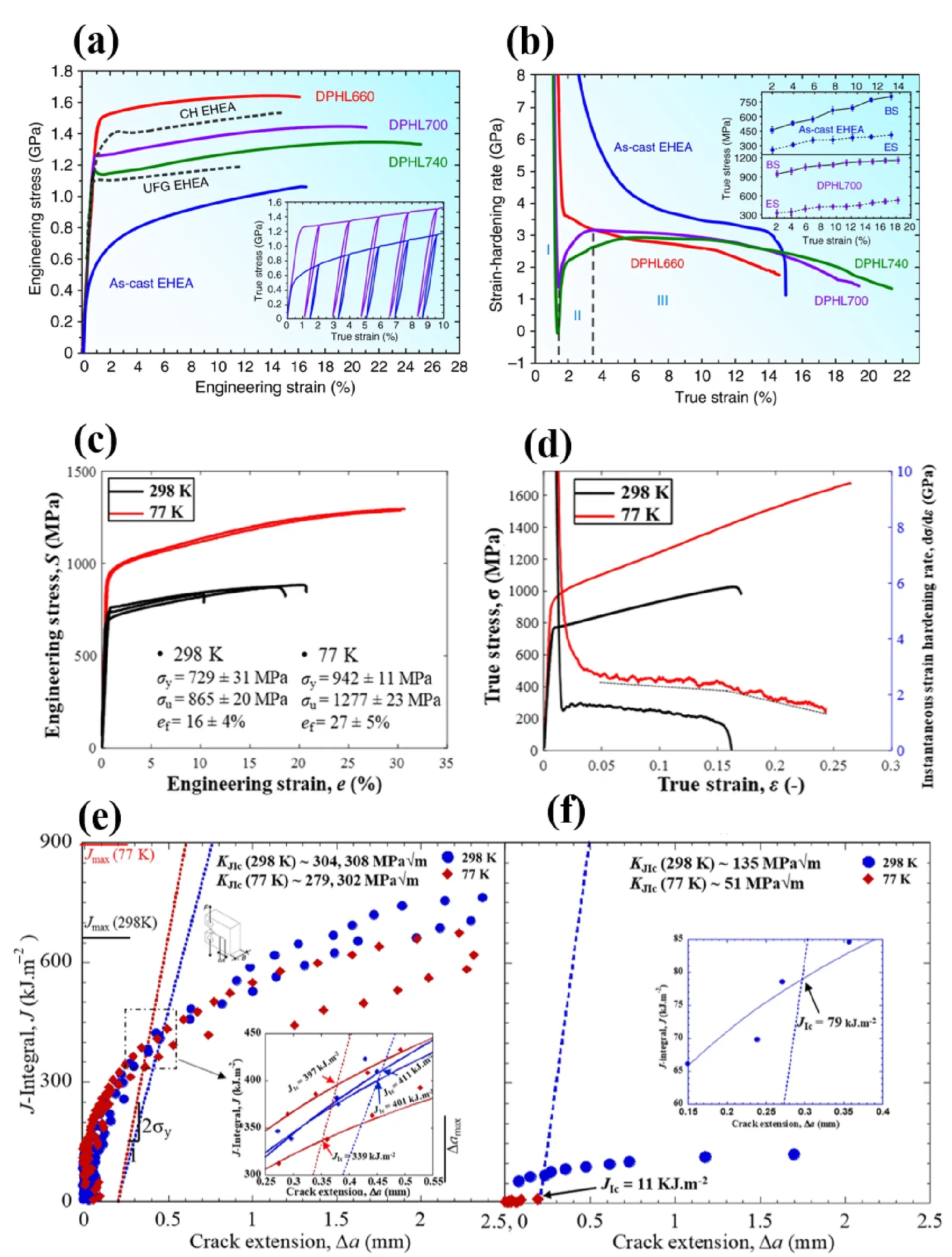
Mechanical behavior and damage tolerance of representative HEAs. (**a**,**b**) Stress–strain and work-hardening behavior of heterogeneous-lamellar AlCoCrFeNi_2.1_ eutectic HEA [38]. (**c**,**d**) Tensile behavior of LPBF Al_0.5_CrCoFeNi at 298 and 77 K. (**e**,**f**) Crack-resistance curves of the as-printed and heat-treated specimens, respectively [160].

Maintaining strength–ductility synergy in HEAs depends less on introducing additional strengthening phases than on preserving strain accommodation throughout deformation. From this perspective, transformation-induced plasticity and heterogeneous dual-phase architectures represent two different routes toward the same objective, namely sustaining work hardening while delaying strain localization. In metastable Fe_50_Mn_30_Co_10_Cr_10_, the progressive transformation of the FCC matrix into HCP during deformation generates new interfaces and continuously renews obstacles to dislocation motion, so strengthening develops together with continued plastic accommodation rather than at its expense [158]. A related effect is achieved in heterogeneous-lamellar AlCoCrFeNi_2.1_, where the mechanical contrast between soft FCC and hard B2 regions promotes strain partitioning, back stress, and geometrically necessary dislocation accumulation [38]. Processing therefore becomes part of the mechanical-design strategy. Rapid solidification during LPBF can generate hierarchical nonequilibrium structures that provide similar multiscale deformation compatibility, as illustrated by Al_19_Co_20_Fe_20_Ni_41_ with a yield strength above 1.3 GPa and a uniform elongation of approximately 20% [159]. The broader implication is that high strength and useful ductility are most likely to coexist when strengthening features remain mechanically compatible with the surrounding matrix during continued loading. Such processing-induced advantages, however, must still be assessed together with porosity, build-direction effects, and post-processing stability before they can be translated from laboratory specimens to engineering components. Across these examples, the mechanical benefit of structural heterogeneity depends on whether the strengthened regions remain compatible with the surrounding deformable regions. The stress–strain curves in Figure 13a reflect the overall tensile response, whereas the work-hardening curves in Figure 13b show the ability of the alloy to sustain strain hardening during continued deformation. In the heterogeneous-lamellar AlCoCrFeNi_2.1_ alloy, coordinated deformation of the FCC and B2 regions generates strain gradients and geometrically necessary dislocations near phase interfaces. The resulting back stress increases the flow stress while sustaining dislocation storage and work hardening, which accounts for the strength–ductility balance shown in Figure 13a,b. A related balance is observed in LPBF Al_0.5_CrCoFeNi, where the increase in strength at 77 K is accompanied by retained tensile ductility rather than premature strain localization, as shown in Figure 13c,d. Damage tolerance, however, is more sensitive to the microstructural state. In Figure 13e,f, Δa represents the stable crack extension, whereas J represents the J-integral describing the crack-driving force and resistance to crack growth. At a given Δa, a higher J value therefore indicates greater resistance to stable crack propagation. At comparable crack extension, the as-printed specimen in Figure 13e exhibits higher J values than the heat-treated specimen in Figure 13f, demonstrating that crack-growth resistance is governed by sustained crack-tip plasticity and shielding rather than by strength alone [160]. Together, these results show that heterogeneous microstructures should be evaluated through deformation compatibility, work-hardening capacity, and crack resistance, not only through their strengthening effect.

Damage tolerance provides a more stringent criterion than tensile strength and ductility because structural failure is ultimately controlled by how the material accommodates deformation in the presence of a crack. In LPBF Al_0.5_CrCoFeNi, decreasing the temperature from 298 to 77 K increased strength while the as-printed material retained good plasticity, sustained work hardening, and high J–Δa crack-growth resistance, as shown in Figure 13c–f [160]. The marked deterioration in crack-growth resistance after heat treatment, particularly at low temperature, shows that strengthening alone does not ensure improved resistance to fracture. What matters is whether the microstructure can continue to redistribute strain and sustain plastic deformation around the crack tip as the applied load increases. This interpretation is also consistent with CrCoNi, where enhanced nanoscale twinning at low temperature preserves crack-tip plasticity and work hardening despite the increase in strength [161]. Under cyclic loading, the same requirement remains relevant because the crack-tip microstructure evolves continuously with loading history. Overload-induced residual stress and deformation twins in CoCrFeMnNi can temporarily retard fatigue-crack growth, while deformable B2 precipitates in Al_0.5_CoCrFeNi can accommodate repeated local strain through dislocation activity and reversible martensitic transformation [162,163]. These results indicate that static toughness, cryogenic damage tolerance, and fatigue resistance are governed by a common requirement: strengthening features must remain mechanically compatible with the surrounding matrix and permit continued strain accommodation in highly stressed regions. The most effective microstructures are therefore not necessarily those with the highest local strength, but those that can delay strain localization and maintain crack-tip plasticity throughout monotonic or cyclic loading.

When this evaluation is extended to additively manufactured components, fatigue behavior is also affected by manufacturing defects and build orientation. Comparison of medium-entropy alloy specimens oriented at 0°, 45°, and 90° relative to the build layers showed that crack initiation was controlled mainly by defect orientation, whereas slip compatibility between adjacent grains and crack deflection became increasingly important during stable crack growth [164]. Stress level also changed the relative contributions of crack initiation and propagation to total life. During the transition from material coupons to practical aerospace components, fatigue reliability therefore cannot be inferred from tensile properties in a single orientation or from average density. Coupling among manufacturing defects, the local microstructure, and the crack-propagation path must also be considered.

Current fatigue evidence remains difficult to compare because load ratio, specimen geometry, environment, and defect populations vary among studies. Overload-induced crack-growth retardation may be temporary because the residual stress and deformation twins responsible for shielding evolve during subsequent cycles [162]. Deformable B2 precipitates can accommodate repeated local strain, but this benefit depends on maintaining phase stability and interfacial compatibility [163]. In additively manufactured alloys, defect orientation may control crack initiation even when bulk density and tensile properties are similar [164]. Evaluation should therefore separate crack initiation from stable propagation and verify whether the proposed mechanism remains effective under representative variable-amplitude loading.

The strength and toughness advantages of HEAs arise from the combined action of several mechanisms, including work-hardening capacity, crack-tip plasticity, and coordinated cyclic deformation. Phase transformations, heterogeneous microstructures, and deformable strengthening phases can maintain plasticity at high strength and increase crack-growth resistance, but whether these advantages persist at the component scale also depends on manufacturing defects and cyclic loading. Existing studies demonstrate the potential of high- and medium-entropy alloys to combine strength, ductility, and low-temperature damage tolerance, but fatigue life, defect sensitivity, and joint-region properties remain insufficiently validated. Aerospace application therefore requires progression from excellent strength and toughness in laboratory specimens to long-term reliability at the component scale. In addition to mechanical loads, the service environment continuously changes the material surface. Particularly in chloride-containing media, segregated regions and manufacturing defects may become preferential sites for localized corrosion, making surface-film stability a new life-controlling factor. Assessment of engineering applicability must therefore also examine passive-film formation, local breakdown, and repassivation behavior in HEAs. Representative strength–ductility and fracture-toughness data are summarized in Table 6.

### 5.2. Corrosion Resistance

Localized corrosion in marine environments does not depend simply on whether an alloy contains corrosion-resistant elements such as Cr, Al, or Mo. More important is how these elements are distributed after solidification, heat treatment, and processing and what type of passive surface film ultimately forms. In MPEAs, elemental partitioning between phases and local precipitation alter the electrochemical stability of different regions, so materials with similar nominal compositions can exhibit markedly different corrosion behavior. In Al_x_CoCrFeNi, increasing Al content raised the fraction of Al/Ni-rich and Cr-depleted BCC regions and consequently reduced local corrosion resistance [165]. Corresponding surface studies further showed that changes in phase structure altered passive-film composition and protective capability [166]. Whether a corrosion-resistant element functions effectively therefore depends on its ability to form a stable protective film in susceptible regions and cannot be judged from its bulk content alone.

**Figure 14:**
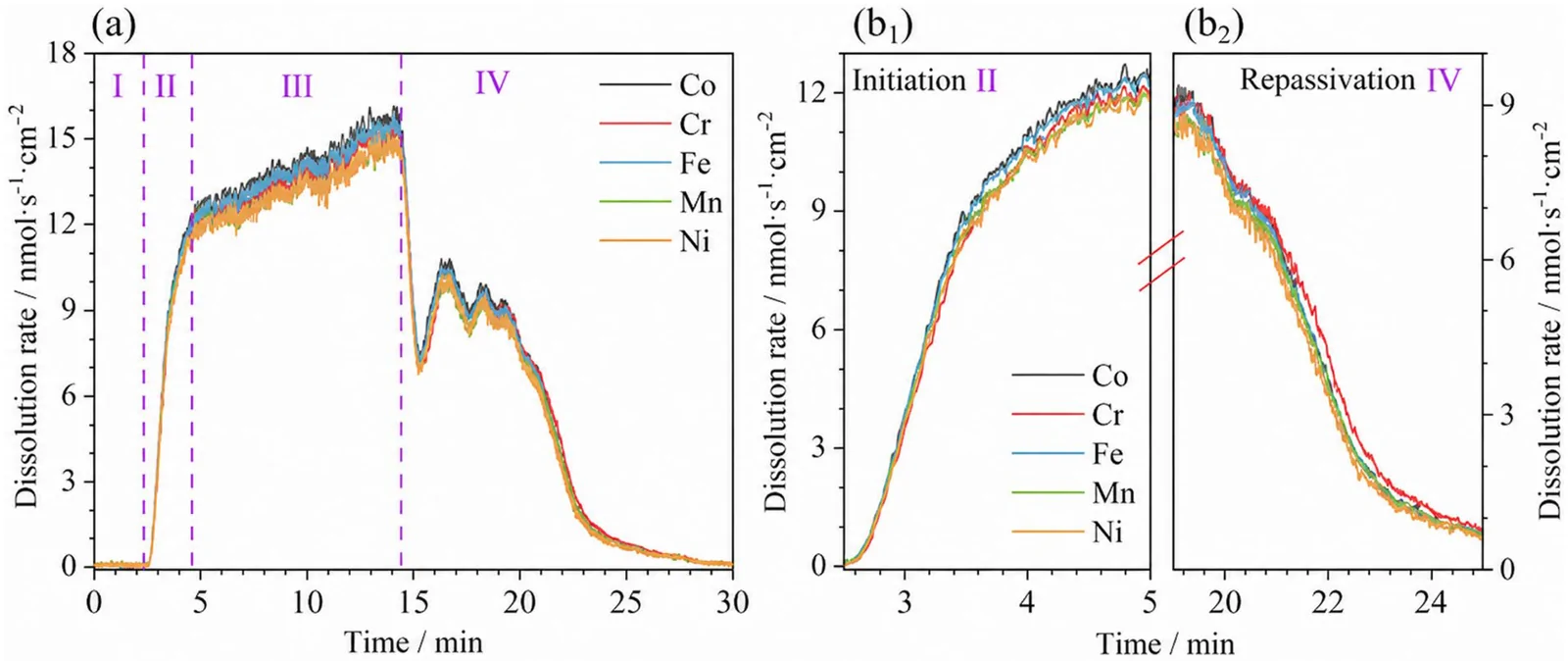
Time-resolved dissolution rates of five alloying elements during pitting corrosion of CoCrFeMnNi: (**a**) complete sequence of incubation, initiation, propagation, and repassivation; (**b_1_**) initiation stage and (**b_2_**) repassivation stage [39]. In (**a**), Roman numerals I–IV and vertical dashed lines delimit incubation, initiation, propagation, and repassivation, respectively; curve colors identify the five elements as shown in the legend.

Processing history influences corrosion resistance primarily by determining the local chemical heterogeneity from which the passive film develops. Variations in thermal history and heat treatment can redistribute Cr and other alloying elements between phases or local regions, thereby changing the electrochemical uniformity of the surface even when the nominal composition remains unchanged [118,167]. This effect is particularly important in multiphase HEAs, where compositional partitioning can create regions with different tendencies for passive-film formation and breakdown. The higher stability of Cr_2_O_3_-rich films in single-phase FCC structures compared with more heterogeneous dual-phase states further indicates that corrosion resistance depends on how effectively protective elements are retained and distributed at the reacting surface rather than simply on their bulk concentration [168]. The relevant question therefore extends beyond whether a passive film can form. Once chloride-induced breakdown occurs, long-term resistance is controlled by the competition between continued local dissolution and restoration of the protective surface state. Time-resolved dissolution measurements of CoCrFeMnNi illustrate this dynamic process. As shown in Figure 14, elemental dissolution evolves from incubation and pit initiation to sustained propagation and then gradually decreases during repassivation after Cl^−^ exposure is removed [39]. The persistence of residual dissolution during this recovery shows that repassivation is not an instantaneous return to the initial surface condition but a progressive reconstruction of the protective film. Corrosion resistance in HEAs should therefore be interpreted as a dynamic ability to maintain and recover surface protection under local chemical perturbation, rather than as a single passive current or breakdown potential measured at one stage of the corrosion process.

These mechanistic measurements clarify passive-film breakdown and recovery, but they do not establish durability under marine service conditions. Localized chloride injection and short-term polarization isolate the kinetics of dissolution and repassivation, whereas sustained flow, crevice occlusion, deposits, and microbial activity can continuously destabilize the surface. Biofilm-induced localized attack in FeCoCrNiMo_0.1_ illustrates how environmental coupling can overcome favorable behavior observed under controlled electrochemical conditions [169]. Long-term evaluation should therefore determine whether repassivation remains effective during repeated breakdown by combining extended immersion or flowing exposure with measurements of pit growth and post-exposure film chemistry. This approach distinguishes temporary electrochemical recovery from durable protection under realistic service conditions. Representative corrosion responses under chloride-containing and marine-related environments are summarized in Table 7.

### 5.3. Oxidation Resistance

**Figure 15:**
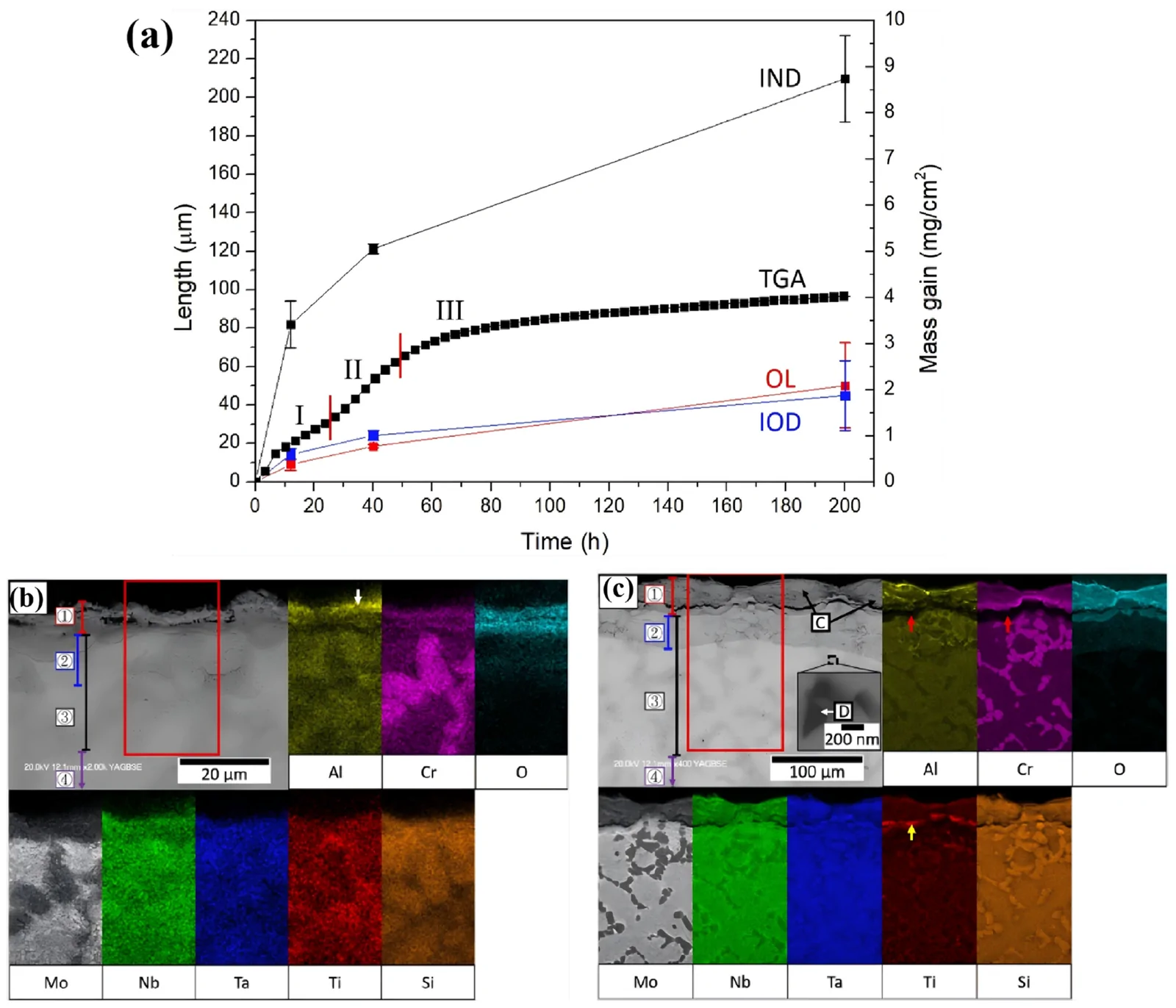
Long-term oxidation behavior of NV1 refractory HEA at 1100 °C. (**a**) Evolution of mass gain and oxide-layer thickness with oxidation time. (**b**) Early-stage cross-sectional morphology and elemental distribution. (**c**) Cross-sectional morphology and elemental distribution after 200 h [171]. Roman numerals I–III in (**a**) denote the three oxidation stages. In (**b**,**c**), ①–④ denote the oxide layer, internal oxidation depth, internal nitridation depth, and substrate, respectively; arrows mark element-enriched or depleted regions. TGA, thermogravimetric analysis; OL, oxide layer; IOD, internal oxidation depth; IND, internal nitridation depth.

At high temperatures, the main challenge in evaluating oxidation resistance is that a low mass gain does not necessarily indicate the formation of a durable protective scale. Mass change reflects the net oxidation kinetics, but it cannot distinguish between a continuous protective layer and a scale that undergoes local cracking, spallation, internal oxidation, or continuous interfacial reconstruction. The oxidation resistance of HEAs should therefore be understood as the ability to establish and maintain a stable reaction layer rather than simply minimize oxide formation. This distinction is evident in Al_0.9_FeCrCoNi, where relatively low mass gains were accompanied by local scale spallation, and in CrMnFeCoNi, where increasing temperature altered both oxidation kinetics and the structure of the reaction layer [18,170]. These observations indicate that mass gain and protective effectiveness are related but not equivalent. A more informative description requires oxidation kinetics to be interpreted together with the continuity, composition, and interfacial stability of the scale. The long-term oxidation behavior of NV1 provides a representative example. Although its mass-gain rate decreased during exposure at 1100 °C, the outer oxide layer, internal oxidation zone, and internal nitridation zone continued to evolve, while local oxidation products gradually developed into a more continuous CrTaO_4_-based layer, as shown in Figure 15 [171]. The reduction in oxidation rate therefore reflects the progressive establishment of a more effective diffusion barrier rather than the cessation of interfacial reactions. From this perspective, protective scale formation is a dynamic process governed by selective oxidation and element transport. Elemental partitioning across oxide scales, changes in migration behavior caused by Al addition, and temperature-dependent oxide-phase evolution further show that the final room-temperature scale represents only the endpoint of a continuously evolving high-temperature reaction [172,173,174]. Consequently, neither high melting point nor low short-term mass gain is sufficient to demonstrate oxidation resistance. The more decisive criterion is whether the alloy can continuously supply scale-forming elements and preserve a dense, adherent, and chemically stable barrier throughout prolonged exposure. This requirement explains why systems capable of sustaining protective phases such as α-Al_2_O_3_ or CrTaO_4_ generally exhibit better long-term oxidation stability, whereas breakdown of scale continuity can lead to rapid oxidation or spallation [175].

This protective capability ultimately depends on the sustained ability of elements such as Al and Cr to form stable oxides. Al_2_O_3_- and Cr_2_O_3_-containing reaction layers in AlCoCrFeNi systems can limit further oxidation [176,177], while changes in Cr content in refractory MPEAs likewise alter scale composition and continuity [178,179]. A high melting point alone therefore does not guarantee excellent oxidation resistance. A continued supply of protective elements and oxide-scale stability are more important. This issue is central to the translation of HEAs into hot-section environments such as turbines. Actual hot-section components do not operate isothermally but undergo repeated heating and cooling during startup, shutdown, and load changes, requiring the protective layer to withstand both oxidation growth and thermal stress. For HEAs used as high-temperature coatings or bond coats, a low isothermal oxidation rate is insufficient. The oxide–substrate interface must remain stable, and continuous cracking and spallation must be avoided during thermal cycling. Depletion of protective elements during long-term service and interdiffusion between the coating and substrate may also progressively alter the original scale-forming capability. High-throughput screening and experimental validation of non-equiatomic Ni-Co-Cr-Al-Fe alloys identified candidate compositions with better oxidation resistance than the compared MCrAlY bond coats and further evaluated their thermal-cycling stability [180]. This result shifts evaluation of HEAs from a simple comparison of oxidation mass gains toward interfacial protection in thermal-barrier systems. For such applications, the relevant requirement is not the lowest mass gain in a single test but the continued formation of a continuous, adherent, and stable protective layer after repeated thermal cycling.

Thermal-cycling durability cannot be inferred from cycle count or net mass change alone. Thermal-expansion mismatch among the scale, coating, and substrate generates interfacial stress. Once cracking or spallation occurs, renewed oxidation consumes additional protective elements. Similar mass changes can therefore represent either a stable thin scale or repeated damage and re-formation. Evaluation of Ni-Co-Cr-Al-Fe coatings demonstrates the need to consider cyclic stability in addition to isothermal oxidation [180]. Meaningful comparison should combine cyclic mass change with scale adhesion, interfacial damage, and post-cycle composition under consistently reported thermal histories.

The oxidation resistance of HEAs depends primarily on whether a protective oxide scale forms reliably and continues to function during long-term thermal cycling. Existing studies have revealed the effects of selective oxidation, elemental diffusion, and phase evolution on scale structure. Under actual hot-section service conditions, however, coating–substrate interdiffusion, depletion of protective elements, and cyclic stability require further validation. Research should therefore progress from isothermal oxidation of small specimens to long-term thermal cycling and coating systems to evaluate hot-section application potential more accurately. Representative high-temperature oxidation data are summarized in Table 8.

### 5.4. Irradiation Resistance

High-energy particles in nuclear environments continuously generate vacancies and interstitials that further develop into dislocation loops and bubbles. The critical requirement for irradiation resistance is therefore not to prevent defect formation completely but to limit long-term accumulation. The complex local chemical environments of MPEAs can alter defect-migration paths. Fewer retained defects were found in NiCoCrFe than in pure Ni after displacement cascades, together with suppressed defect-cluster growth [181]. Further analysis indicated that interstitial clusters in multicomponent alloys preferentially undergo short-range three-dimensional migration, increasing their probability of recombination with vacancies [182]. Multi-principal-element alloying can therefore suppress irradiation-damage accumulation by altering defect-migration paths and promoting vacancy-interstitial recombination rather than simply reducing diffusion rates.

**Figure 16:**
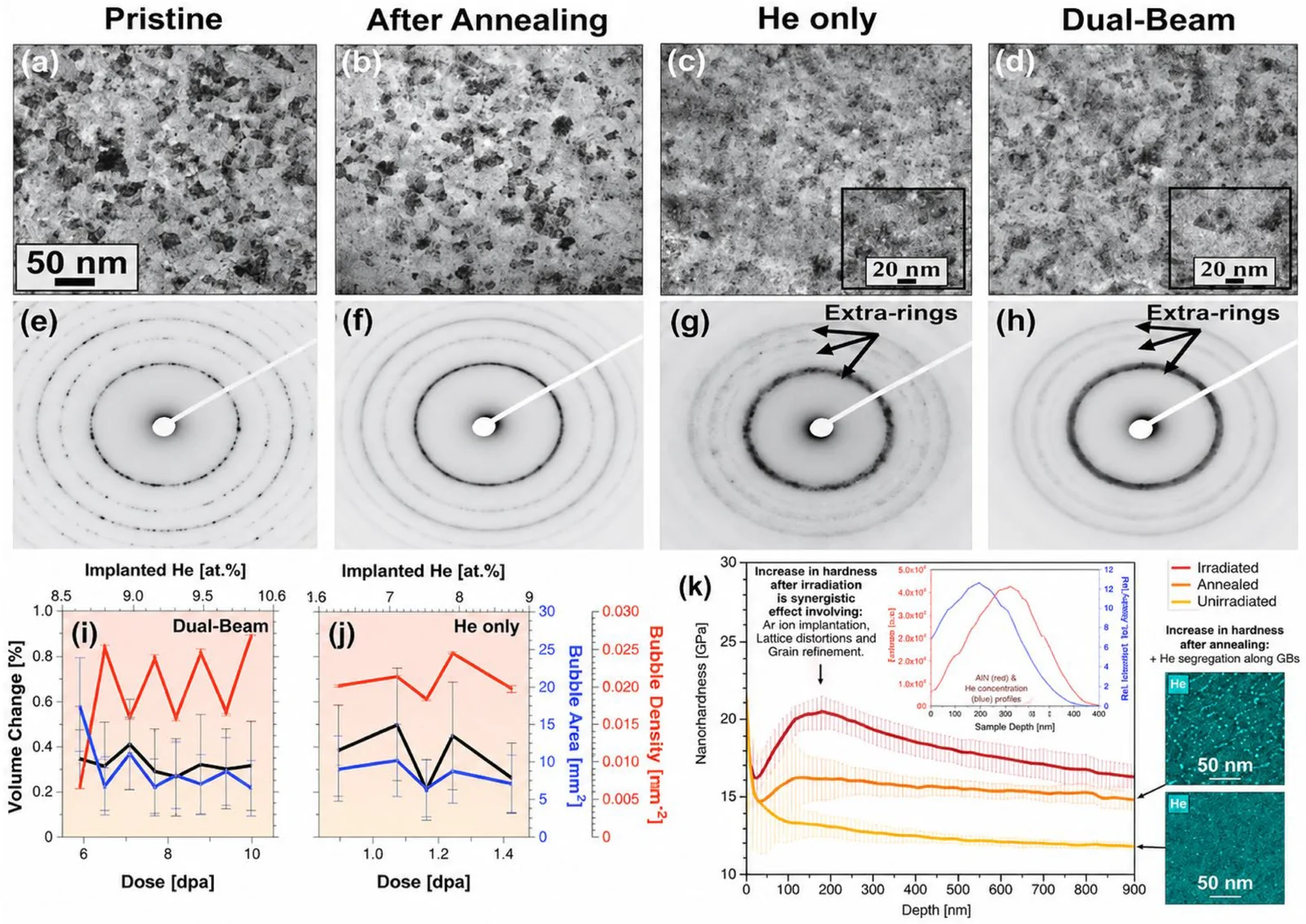
Irradiated microstructure and mechanical response of nanocrystalline WTaCrVHf refractory HEA: (**a**–**d**) bright-field TEM images of the as-deposited, annealed, single-beam He-implanted, and dual-beam-irradiated states, respectively; (**e**–**h**) corresponding selected-area electron diffraction patterns; (**i**,**j**) dose-dependent changes in volume, bubble area, and bubble density under dual-beam irradiation and single-beam He implantation, respectively; (**k**) nanoindentation hardness–depth curves for the unirradiated, annealed, and irradiated states, together with the Hf distribution showing grain-boundary segregation after irradiation [40]. The arrows in (**g**,**h**) mark additional diffraction rings, and the arrows in (**k**) link the hardness curves to the corresponding He-distribution maps.

Defect regulation in nanocrystalline HEAs should be viewed as a dynamic competition between continuous defect production and the capacity of interfaces to absorb and eliminate those defects. A high density of grain boundaries can shorten defect-migration distances and provide efficient sinks for vacancies and interstitials, but this advantage is meaningful only if the interfaces themselves remain structurally and chemically stable during irradiation. The refractory WTaCrVHf alloy illustrates this distinction. Its nanocrystalline structure was largely retained after He implantation and dual-beam irradiation, while Hf enrichment near grain boundaries contributed to interfacial stabilization [40]. As shown in Figure 16, irradiation still generated bubbles, local structural changes, and measurable hardening, indicating that grain boundaries did not prevent damage formation. Instead, they restricted the accumulation and growth of irradiation-induced defects sufficiently to delay large-scale microstructural degradation. This distinction is important because a low defect density at a given dose does not necessarily imply intrinsic resistance to defect production. It may instead reflect a high rate of defect removal at stable interfaces. The effectiveness of this mechanism can also change during irradiation because segregation progressively modifies grain-boundary chemistry and local defect energetics. Observations of irradiation-induced Ni segregation and composition-dependent defect evolution in CrCoNi further demonstrate that the interfacial state is not fixed but evolves together with the damage structure [183,184]. Irradiation resistance in nanocrystalline HEAs should therefore be evaluated in terms of the sustained sink efficiency and stability of the grain-boundary network rather than grain size or initial defect density alone. From an engineering perspective, the more relevant question is whether these interfaces can continue to absorb defects without undergoing segregation-induced embrittlement, phase instability, or loss of mechanical integrity as dose and temperature increase.

In addition to defect migration and interfacial effects, irradiation can directly alter phase stability. Irradiation of Al_0.3_CoCrFeNi with 3 MeV Au ions showed that precipitation of (Ni, Al)-rich nanoclusters and the L1_2_-ordered structure was suppressed from 250 to 500 °C, whereas B2 precipitation accelerated at 650 °C [185]. The authors attributed this temperature-dependent behavior to competition between ballistic dissolution and irradiation-enhanced diffusion, demonstrating that irradiation responses also involve pronounced phase evolution. When the irradiation environment additionally contains transmutation products such as He, suppression of dislocation loops alone is insufficient to ensure long-term stability. Although WTaCrV can reduce the formation of large defect clusters, numerous small bubbles still develop at high He contents [186]. Regulation of bubble evolution using interstitial elements or second-phase interfaces provides another approach. For example, adding a small amount of C to an FeMnCoCr based alloy suppressed both He-bubble growth and irradiation hardening [187], while grain boundaries and oxide interfaces in ODS-NiCoFe limited void formation, although some hardening remained after irradiation [188]. Evaluation of irradiation resistance must therefore consider both displacement damage and gas accumulation rather than use a single defect metric to assess irradiation tolerance, particularly for nuclear-energy applications. W-based refractory MPEAs have high-temperature stability and are therefore more relevant to plasma-facing materials in fusion devices, but He bubbles, irradiation embrittlement, and changes in thermophysical properties may still limit long-term service. NiCoFe-type MPEAs offer considerable scope for microstructural control and may be candidates for advanced reactor structural materials, but irradiation-induced segregation and retention of mechanical properties require further validation [189]. Most current studies use ion irradiation for mechanistic screening, but the resulting damage state differs from that produced in an actual neutron environment. For nuclear-energy applications, evaluation must expand from microscopic defect evolution to post-irradiation mechanical and thermophysical properties, with long-term neutron irradiation used to verify service reliability.

The irradiation-resistance advantage of HEAs does not arise from complete suppression of irradiation defects. Instead, complex local chemical environments and stable interfaces alter defect migration and recombination and thereby delay damage accumulation. Existing studies show that this regulation can reduce dislocation-loop formation and void swelling to some extent and improve microstructural stability after irradiation. However, most current evidence is derived from short-duration ion irradiation and should be treated as mechanistic screening rather than direct prediction of service life under neutron irradiation. Irradiation-induced phase evolution varies strongly with temperature [185], while He bubbles may continue to accumulate even when large defect clusters are suppressed [186]. Interface-assisted defect removal can reduce visible damage, but residual hardening shows that low defect density does not necessarily indicate retained mechanical performance [187,188]. In actual nuclear environments, prolonged high-temperature neutron exposure also generates He and H and may progressively degrade mechanical integrity and thermal transport [189]. Long-term assessment of HEAs for nuclear structural and fusion-related applications must therefore connect defect, gas, and phase evolution with performance retention under representative irradiation conditions. Representative quantitative irradiation responses are summarized in Table 9. Recent ion-irradiation studies add temperature-resolved loop, void, and hardening data for chemically heterogeneous NiCoFeCrMn-C,N, CoCrFeMnNi, and FeNiMnCr alloys [140,190,191].

### 5.5. Catalytic Performance

The multielement nearest-neighbor configurations and diverse local chemical environments of HEAs provide a broad compositional space for regulating adsorption energies and electronic structures in catalytic reactions and have consequently attracted substantial attention in electrocatalysis in recent years [192]. Their catalytic advantages, however, are not directly determined by high bulk configurational entropy but depend primarily on the working surface in actual contact with the reactants. Local sites formed by different elements can alter charge distributions and intermediate adsorption, while a sustained potential may cause surface segregation and reconstruction. Evaluation of HEA catalysts must therefore consider the specific elemental combinations and evolution of the actual active sites during reaction. Screening a combinatorial library of multielement transition-metal nanoparticles for noble-metal-free oxygen reduction reaction (ORR) catalysts revealed pronounced activity differences among compositions, showing that increasing the number of components alone does not ensure better catalytic performance [193]. High-entropy metallic-glass nanoparticles further demonstrate that a multicomponent composition can regulate different electrocatalytic functions on a single material platform [194]. Such compositional advantages can be interpreted meaningfully only when they are linked to specific active sites. In FeCoNi_x_Ru, local charge redistribution made Co more favorable for water dissociation, while Ru provided suitable H* adsorption [195]. On an atomically controlled Pt/CrMnFeCoNi model surface, Pt gradually became enriched near the surface after potential cycling [196]. The initial composition is therefore only the starting point for a catalytic surface. The local structure formed and maintained under operating conditions ultimately controls the reaction.

The catalytic advantage of HEAs should be interpreted in terms of the local coordination environment that is created and maintained under operating conditions rather than nominal multielement composition alone. Deliberate construction of multicomponent atomic arrangements can broaden the distribution of adsorption energies and provide neighboring sites with complementary functions, allowing different elementary steps to be optimized within the same surface. This principle has been demonstrated in PtRuFeCoNi atomic layers, where multielement coordination regulated hydrogen adsorption and improved HER and HOR kinetics [197]. However, the initial active-site configuration is only a starting state because electrochemical operation continuously modifies surface composition and structure. APT observations of a Cantor-alloy catalyst before and after OER showed that both the oxygen-affected depth and metallic distribution changed during reaction, confirming that the working surface can differ substantially from the as-prepared state [198]. The key question is therefore not simply whether a favorable local configuration can be created, but whether it can persist or reconstruct into another catalytically effective state during prolonged operation. This distinction becomes increasingly important when performance is translated from half-cell measurements to practical devices. Multielement coordination in PtRuNiCoFeMo nanowires enhanced alkaline HOR kinetics, while nanoporous HEAs and PtFeCoNiCu catalysts further demonstrated that high activity can be accompanied by substantial cycling stability and measurable PEMFC output [199,200,201]. These results indicate that the multisite concept can extend beyond model reactions, but device-level performance depends on the stability of the working surface under sustained potential, reactant transport, and membrane-electrode conditions. HEA catalysis should therefore be evaluated not only by peak activity, but by whether the active coordination environment remains chemically and structurally functional throughout long-term operation.

**Figure 17:**
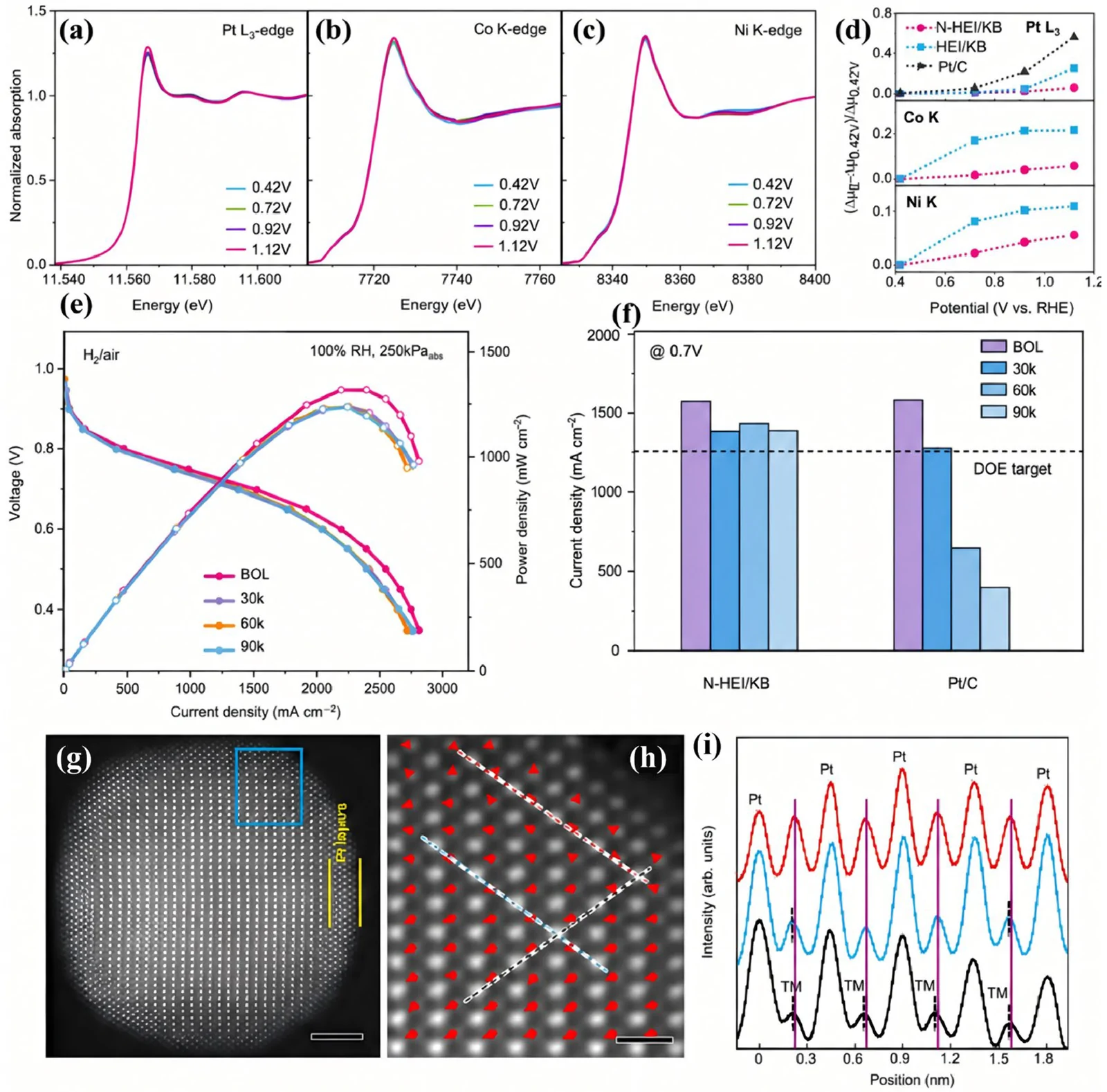
Working-state and post-cycling characterization of N-doped L10-PtCoNiFeCu high-entropy intermetallic catalyst. (**a–c**) Operando XANES spectra at the Pt L3-, Co K-, and Ni K-edges. (**d**) Potential-dependent absorption responses. (**e**) Polarization and power-density curves. (**f**) Current-density retention at 0.7 V. (**g**,**h**) Post-cycling atomic-resolution STEM images showing the ordered atomic structure. (**i**) Atomic-column intensity profiles after cycling [204]. Red arrows in (**h**) mark transition-metal-column displacements; dashed lines in (**h**) indicate the scan directions used for the intensity profiles in (**i**), with matching colors. The horizontal dashed line in (**f**) denotes the DOE target. Colors elsewhere correspond to the potentials, catalysts, cycling stages, or scan directions identified in the individual legends.

Further improvement in device-level performance requires more than the creation of highly active surface sites. The central challenge is to preserve the favorable local coordination and strain state as the catalyst undergoes repeated electrochemical cycling. Surface asymmetry, antisite defects, and local strain can all enhance ORR kinetics by modifying the electronic environment of Pt-based active sites, as demonstrated in PtPdFeCoNi and PtZnFeCoNiCr systems [202,203]. However, these structural advantages are useful only when they remain effective under prolonged operating conditions. This consideration shifts the design criterion from maximizing the initial activity to stabilizing the working surface against elemental migration, structural relaxation, and electrochemical reconstruction. N-doped L1_0_-PtCoNiFeCu provides a representative example because metal–N interactions restrict elemental redistribution while local strain helps preserve the coordination environment around Pt [204]. As shown in Figure 17, the catalyst maintained a current density of 1388 mA·cm^−2^ at 0.7 V after 90,000 cycles under heavy-duty vehicle H_2_/air membrane-electrode conditions. This result suggests that durability should be interpreted as retention of a functional working structure rather than simply as a small loss in an electrochemical performance metric. A second limitation emerges when such structurally optimized catalysts are considered for practical deployment. Complex multielement compositions and nanoscale architectures are valuable only if they can be produced with reproducible particle size, composition, and active-site distribution at larger scales. Continuous-flow spray pyrolysis, which produced gram-scale carbon-supported high-entropy nanoparticles with sub-2 nm average particle sizes and approximately 30 wt.% metal loading, demonstrates one possible route toward this transition [205]. Practical HEA electrocatalysis therefore requires three conditions to be satisfied simultaneously: high intrinsic activity, structural stability of the working surface, and scalable synthesis with controlled compositional uniformity. Representative electrocatalytic activity and durability data are summarized in Table 10, including alkaline HOR, acidic HER, OER, water electrolysis, ORR, and PEMFC systems [206,207,208].

Cycle number alone is insufficient for evaluating electrocatalytic durability because the reported current may include initial activation, gradual deactivation, or abrupt structural failure. APT observations before and after OER show that the affected depth and elemental distribution of the working surface change during operation [198]. Stability should therefore be linked to post-operation evidence of elemental dissolution, particle coarsening, and active-site reconstruction. The 90,000-cycle result for N-doped L1_0_-PtCoNiFeCu provides stronger service-relevant evidence because device-level performance retention was evaluated together with the post-cycling structure [204]. Comparisons among catalysts should also report the operating window, applied load, reaction environment, and device configuration.

The catalytic advantages of HEAs arise mainly from the synergistic regulation of local electronic structures and intermediate adsorption by multielement nearest-neighbor configurations. As research progresses, the focus is shifting from increased activity under half-cell conditions toward membrane electrodes with low noble-metal loading and long-term cycling stability. Existing results show that rational regulation of surface structure and local strain can further improve reaction kinetics, but the actual working surface continuously reconstructs with potential and reaction environment. High initial activity therefore does not mean that the same active state will persist during long-term operation. Future research should pay greater attention to working-surface evolution and clarify how elemental dissolution and particle growth cause device-performance decay. Scalable synthesis must also maintain consistent particle-size and compositional distributions. Only by considering activity, durability, and manufacturability together can the local-site advantages of high-entropy catalysts be converted into stable device performance and application value. Although different properties correspond to different failure processes, translation from material advantages to engineering applications requires the critical control variables to be identified and stability to be validated under conditions closer to actual service.

## 6. Conclusions and Outlook

The central scientific conclusion of this review is that nominal composition does not uniquely define the material state of an HEA or MPEA. HEA retains its conventional composition-based and entropy-based meanings, while MPEA provides a broader description of alloys governed by several principal elements. Ideal configurational entropy describes compositional complexity but assumes random atomic occupation and cannot directly establish chemical order or single-phase stability. The actual phase constitution results from temperature-dependent free-energy competition, element-specific chemical interactions, diffusion kinetics, and the complete processing history. The high-entropy, lattice-distortion, sluggish-diffusion, and cocktail effects should therefore be treated as diagnostic concepts rather than universal mechanisms. Their physical relevance must be established through direct evidence of the phase constitution, local chemical environment, defect structure, diffusion pathway, and corresponding properties of the material state under investigation.

Fabrication provides the critical link between nominal composition and the resulting microstructure. Mechanical alloying promotes solid-state mixing but may introduce contamination and residual porosity, while vacuum melting enables bulk production but remains sensitive to segregation, inclusions, element loss, and homogenization history. Severe plastic deformation modifies a pre-existing state and can generate gradients in grain size, texture, and defect density. Magnetron sputtering and additive manufacturing provide access to nonequilibrium structures, although composition transfer, residual stress, position dependence, and post-processing stability require careful control. Identical nominal compositions may consequently develop different phase fractions, segregation states, defects, and properties through different processing routes. Reproducibility must therefore be evaluated from the measured composition, microstructure, and property distribution across representative positions and independent batches rather than from nominal parameters alone. Computational methods can reduce the composition and processing search space, but CALPHAD, atomistic calculations, phase-field modelling, and machine learning remain sensitive to database coverage, model assumptions, and training-data quality. Their predictions should be linked with multiscale characterization and experimental validation to establish consistent relationships among processing history, average phase constitution, local structure, surface state, and dynamic evolution.

The engineering significance of HEAs depends on whether their functional material states can be retained during service. High strength is useful only when work-hardening capacity, damage tolerance, and cyclic resistance are maintained. Corrosion and oxidation resistance depend on the continued integrity and recovery of protective surface layers rather than on an initial electrochemical parameter or short-term mass gain alone. Irradiation tolerance requires control of defect and gas accumulation together with retention of mechanical and thermophysical properties. Electrocatalytic activity is governed by the working surface, which may reconstruct, dissolve, or coarsen during prolonged operation. These examples establish a common evaluation principle. Peak performance under a single laboratory condition cannot demonstrate engineering reliability. Loading or exposure history must be connected with microstructural evolution, retained performance, and the dominant failure mode. Long-term assessment should therefore include cyclic mechanical loading, thermal cycling, prolonged environmental exposure, irradiation-induced structural evolution, and device-level electrochemical operation under clearly reported and comparable conditions.

Current limitations include inconsistent terminology, incomplete processing metadata, differences in sampling and testing protocols, insufficient statistical reporting, and a lack of long-term service data. Many reported properties are obtained from small specimens or single processing batches, while high-resolution characterization frequently represents only a limited material volume. Scale-up may further change heat and mass transfer, element loss, defect formation, and local thermal history, making laboratory performance difficult to reproduce in larger components. Future research should prioritize standardized reporting of actual composition, complete processing history, sampling position, structural variation, property scatter, and service-test conditions. Scale-up should be supported by process monitoring and position-resolved verification, while computational screening should be combined with targeted synthesis, multiscale characterization, and uncertainty assessment. Long-duration testing under representative mechanical, environmental, irradiation, and electrochemical conditions is required to establish realistic degradation pathways. Sustainability must also be evaluated through critical-element dependence, processing energy, feedstock reuse, component lifetime, repairability, and end-of-life recovery. The future value of HEAs and MPEAs will ultimately depend not on the number of constituent elements or the highest reported property, but on whether a designed material state can be produced reproducibly, scaled economically, maintained during service, and recovered responsibly.

## Figures and Tables

**Table 1:** Selected recent reviews and the scope of the present review.

Review	Focus	Main Limitation	Extension Here
Krishna et al. [25]	General processing and applications	Limited processing-to-service linkage	Unified processing–state–service framework
Han et al. [27]	Additive manufacturing	Single-route focus	Cross-route comparison
Pan et al. [28]	High-temperature behavior	High-temperature focus	Cross-environment service comparison
Li et al. [31]	Corrosion and passive films	Corrosion-specific scope	Links surface state to processing and service
Jiménez-Arévalo et al. [32]	Electrocatalysis and computational design	Catalysis-specific scope	Links working surface to local structure
Cao et al. [33]	Films/coatings and interfaces	Film/coating focus	Extends to bulk routes and reproducibility

**Table 2:** Meanings and limitations of the four core effects.

Core Effect	Meaning	Limitation	References
High-entropy effect	Configurational entropy alters free-energy competition among disordered and ordered phases, intermetallic compounds, and phase-separated states	Candidate-phase free energies must be compared, the effect cannot independently establish single-phase formation or stability over the full temperature range	[44,45,46,65]
Lattice distortion	Local displacements and energy fluctuations affect dislocations, point defects, diffusion, and transport	Requires quantitative local-structure evidence, δ or peak broadening alone is insufficient	[65,66,67]
Sluggish diffusion	Reduced mobility of specific species in a particular phase, along a particular path, and within a particular temperature range	Conclusions depend on the diffusing species, phase state, defect path, and temperature	[53,54,55,56,57,58,59,60,61,62,63,64,65]
Cocktail effect	Elements, phases, interfaces, and defects produce an identifiable nonadditive response	Lacks a unique physical quantity and must be interpreted through the specific structure	[65,68,69,70]

**Table 3:** Comparison of processing routes and the resulting HEA and MPEA material states.

Route	Process History	Grain Size and Texture	Segregation and Defects	Phase Constitution and Local Order	Refs.
Mechanical alloying	Solid-state milling followed by compaction and consolidation	Nanocrystalline or ultrafine grains. Texture develops mainly during consolidation	Avoids solidification segregation, but O/C contamination, wear debris, and residual pores may occur	Milling can retain metastable phases. Consolidation may form B2, carbides, or oxides. Local order requires direct verification	[6,73,78,79,80,81,82,83,84,85,125,126,127]
Vacuum melting	Liquid mixing, solidification, and optional homogenization	Dendritic, columnar, or equiaxed grains controlled by heat extraction	Solidification segregation, shrinkage, inclusions, incomplete dissolution, and volatile-element loss	As-cast states may be metastable or multiphase. Heat treatment can drive redistribution, ordering, precipitation, or coarsening	[7,86,88,89,90,91,92,93,94,95]
Severe plastic deformation	HPT or ECAP imposes large shear on a pre-existing material	Approximately 50–100 nm grains with shear texture and spatial gradients	High dislocation, twin, and grain-boundary densities. Uniformity depends on position and strain	A single phase may be retained, while annealing can cause decomposition. Local order requires direct measurement	[96,97,98,99]
Magnetron sputtering	Nonequilibrium atom-by-atom deposition controlled by target, substrate, and plasma	Nanocrystalline or amorphous structures. Texture depends on adatom mobility	Composition-transfer errors, residual stress, column boundaries, and voids	Metastable or amorphous states are accessible. Local order requires verification after deposition or thermal exposure	[100,101,102,103,104,105,106,107,108]
Additive manufacturing	Layerwise melting, rapid solidification, and repeated thermal cycling	Columnar or equiaxed grain hierarchy with build-direction texture	Melt-pool segregation, pores, cracks, unmelted particles, and residual stress	Metastable and position-dependent phases may form. Local order requires verification after processing or thermal exposure	[9,11,34,109,110,111,112,113,114,115,116,117,118,119,120,121,122,123]

**Table 4:** Representative quantitative comparisons of processing-dependent material states at nominally identical compositions.

Alloy	Processing Contrast	Microstructural Change	Property Change	Ref.
CoCrFeMnNi	HPT versus HPT followed by 450 °C annealing	Approximately 50 nm single-phase FCC changes to nanoscale decomposition products	Strength 1950 MPa and hardness 520 HV after HPT. Hardness increases to 910 HV after 100 h	[97]
AlCrTiVNb_0.5_	As-cast versus 1000 °C for 2 h	Predominantly BCC changes to BCC, HCP, and FCC	Hardness increases from 543 to 725 HV	[93]
Al_0.5_NbTa_0.8_Ti_1.5_V_0.2_Zr	Slow-cooled 1200 °C state versus 1400 °C solution treatment and 600 °C aging for 120 h	Continuous B2 matrix with BCC particles changes to discrete B2 precipitates in BCC	Compressive yield strength decreases from 2032 to 1345 MPa. Fracture strain is 4.7% in the slow-cooled state, while first-crack strain reaches 38% after aging	[95]
AlCoCrFeNi	EB-PBF top versus bottom region	Almost no FCC at the top versus approximately 30% FCC at the bottom	E_corr_ −326 to −202 mV and i_corr_ 0.67 to 0.22 mA·cm^−2^. Passivity appears only at the bottom	[118]
AlCrFe_2_Ni_2_	L-DED scan speed 6.2 versus 3.3 mm s^−1^	BCC/B2-dominated state with cracking changes to a greater FCC fraction	Yield strength decreases from 1120 to 960 MPa, while elongation rises from 2.2% to 10.3%	[120]
WTaMoNbV	As-built LPBF versus hot isostatic pressing	Internal cracks are reduced	Compressive ultimate strength increases from 1094 to 1220 MPa	[121]

Note: The electrochemical data for AlCoCrFeNi were measured in 3.5 wt.% NaCl at room temperature, and potentials are reported versus Ag/AgCl [118].

**Table 5:** Comparison of multiscale characterization techniques for HEAs and MPEAs.

Technique	Typical Length Scale	Measurable Structural Features	Corresponding Property Information	Main Limitation	Refs.
XRD and synchrotron XRD	Bulk average with micrometer- to millimeter-scale sampling	Phase constitution, phase fraction, and lattice strain	Phase and thermal stability	Limited sensitivity to local order and minor phases	[149,150,151]
SEM, EBSD, EDS, and EPMA	Approximately 0.1–100 μm	Grain size, texture, phase distribution, and segregation	Deformation heterogeneity and crack initiation	Atomic-scale features are not resolved	[36,136,137,138,139]
TEM, STEM, SAED, and ECCI	Atomic to nanoscale resolution with micrometer-scale fields of view	Dislocations, stacking faults, interfaces, and local order	Strengthening and deformation mechanisms	Small sampling volume	[37,130,136,152,153,154,155]
APT	Subnanometer resolution within analysis volumes of tens to hundreds of nanometers	Elemental partitioning, nanoscale segregation, and local order	Precipitation strengthening and phase stability	Limited volume and reconstruction uncertainty	[35,134,135,139,140]
XPS and ToF-SIMS	Several nanometers for XPS and nanometers to micrometers for ToF-SIMS	Valence states, film composition, and depth distribution	Corrosion and oxidation stability	Sputtering damage and depth-calibration uncertainty	[141,142,143,144,145,146,147,148]
In situ XRD, TEM, and neutron diffraction	Atomic to nanoscale for TEM and bulk averaged for diffraction	Phase transformation, load partitioning, and defect evolution	Deformation pathways and thermal stability	Restricted test conditions and limited representativeness	[5,16,37,149,150,151,152,153,154,155,156,157]

**Table 6:** Representative strength–ductility and fracture-toughness properties of HEAs and MPEAs. RT denotes room temperature.

Alloy	State	Temperature	YS (MPa)	UTS (MPa)	Ductility (%)	*K_JIc_* (MPa·m^1/2^)	Ref.
Fe_50_Mn_30_Co_10_Cr_10_	TRIP-DP	RT	—	~850	~70	—	[158]
AlCoCrFeNi_2.1_	DPHL660	RT	1490	—	~16	—	[38]
Al_19_Co_20_Fe_20_Ni_41_	LPBF	RT	1311	1630	—	—	[159]
Al_0.5_CrCoFeNi	LPBF	298 K	729 ± 31	865 ± 20	16 ± 4	~306	[160]
Al_0.5_CrCoFeNi	LPBF	77 K	942 ± 11	1277 ± 23	27 ± 5	~290	[160]
CrCoNi	Wrought	293 K	440 ± 13	884 ± 11	73 ± 1	208 ± 25	[161]
CrCoNi	Wrought	77 K	657 ± 22	1311 ± 27	90 ± 2	273 ± 32	[161]

An em dash (—) indicates that the value was not reported or was unavailable.

**Table 7:** Representative corrosion responses of HEAs under chloride-containing and marine-related environments.

Alloy State	Test Condition	E_corr_ (mV)	i_corr_ (mA·cm^−2^)	E_pit_ (mV)	Other Quantitative Result	Ref.
AlCoCrFeNi, EBM top	3.5 wt.% NaCl, RT	−326 ± 16	0.67 ± 0.09	—	No apparent passivity	[118]
AlCoCrFeNi, EBM bottom	3.5 wt.% NaCl, RT	−202 ± 17	0.22 ± 0.05	209 ± 10	Lowest i_corr_ of the three states	[118]
AlCoCrFeNi, cast	3.5 wt.% NaCl, RT	−160 ± 38	0.36 ± 0.19	214 ± 6	—	[118]
FeCoCrNiMo_0.1_	P. aeruginosa medium, 14 d, nutrients 100%→10%	—	—	—	Dissolved Fe: 188.4→712.9 μg·L^−1^	[169]
TiNbTaMo, as-cast	0.9 wt.% NaCl, 25 °C	−536 vs. SCE	—	—	Stable passive response under the stated polarization protocol	[167]
CrFeNi_2_	3.5 wt.% NaCl, RT	—	—	954 vs. Ag/AgCl	Passive range ~1035 mV, strong repassivation	[168]

An em dash (—) indicates that the value was not reported or was unavailable.

**Table 8:** Representative high-temperature oxidation metrics of HEAs and MPEAs.

Alloy	Atmosphere	Time (h)	Temperature (°C)	Mass Gain (mg·cm^−2^)	Dominant Scale or Oxide	Ref.
AlTiVCr	Air	24	700	3.00	Ti/V-rich oxides	[18]
AlTiVCr	Air	24	900	17.4	Ti/V-rich oxides	[18]
Al_0.9_FeCrCoNi	Air	24	700	0.12	Al_2_O_3_	[18]
Al_0.9_FeCrCoNi	Air	24	900	0.55	Al_2_O_3_	[18]
CrMnFeCoNi	21% O_2_/79% N_2_	24	900	1.76	Cr/Mn-rich oxides	[170]
CrMnFeCoNi	21% O_2_/79% N_2_	24	1000	4.45	Cr/Mn-rich oxides	[170]
CrMnFeCoNi	21% O_2_/79% N_2_	24	1100	9.08	Cr/Mn-rich oxides	[170]
NV1 refractory HEA	Air	200	1100	~4.03	CrTaO_4_	[171]
AlCoCrNiNb_0.2_	Air	100	1000	0.65	Cr-rich protective oxide	[179]
AlCoCrNiNb_0.2_	Air	100	1100	1.31	Cr-rich protective oxide	[179]

**Table 9:** Representative quantitative irradiation responses of HEAs and MPEAs.

Alloy	Irradiation Condition	Temperature	Quantitative Metric	Value	Ref.
W_29.4_Ta_42_Cr_5.0_V_16.1_Hf_7.5_ thin film	1 MeV Kr^2+^ and 16 keV He^+^, 8.5 dpa	1173 K	Volume change	~0.3%	[40]
W_31_Ta_34_Cr_5_V_27_Hf_3_ thick film	400 keV Ar^2+^, 10 dpa	1073 K	Hardness increase	3.75 GPa	[40]
Fe_50_Mn_30_Co_10_Cr_10_	400 keV He^+^, 1 × 10^17^ cm^−2^	450 °C	Swelling	1.50%	[187]
FeMnCoCrNi	400 keV He^+^, 1 × 10^17^ cm^−2^	450 °C	Swelling	0.19%	[187]
Fe_49.5_Mn_30_Co_10_Cr_10_C_0.5_	400 keV He^+^, 1 × 10^17^ cm^−2^	450 °C	Swelling	0.15%	[187]
ODS-NiCoFeCr	3 MeV Ni^2+^, ~101 dpa	580 °C	Post-irradiation hardness	5.63 ± 0.87 GPa	[188]
NiCoFeCrMn-C,N	3 MeV Ni^2+^, ~30 ± 5 dpa	420 °C	Hardening	1.43 GPa	[140]
NiCoFeCrMn-C,N	3 MeV Ni^2+^, ~30 ± 5 dpa	480 °C	Hardening	1.25 GPa	[140]
NiCoFeCrMn-C,N	3 MeV Ni^2+^, ~30 ± 5 dpa	540 °C	Hardening	0.98 GPa	[140]
CoCrFeMnNi	19.6 MeV Fe, peak 62.3 dpa	RT	Mean loop size	24.3 nm	[190]
CoCrFeMnNi	19.6 MeV Fe, peak 62.3 dpa	500 °C	Mean loop size	83.2 nm	[190]
Fe_27_Ni_28_Mn_27_Cr_18_	3 or 5.8 MeV Ni, 0.03 dpa	RT	Hardness increase	~35%	[191]
Fe_27_Ni_28_Mn_27_Cr_18_	3 or 5.8 MeV Ni, 0.3 dpa	RT	Hardness increase	~80%	[191]
Fe_27_Ni_28_Mn_27_Cr_18_	3 or 5.8 MeV Ni, 3 dpa	500 °C	Hardness increase	<20%	[191]

**Table 10:** Representative activity and durability metrics of HEA-based electrocatalysts. HOR, ORR, HER, OER, PEMFC, PEMWE, and HDV denote hydrogen oxidation, oxygen reduction, hydrogen evolution, oxygen evolution, proton-exchange membrane fuel cell, proton-exchange membrane water electrolysis, and heavy-duty vehicle conditions, respectively.

Catalyst	Reaction	Test Condition	Activity Metric	Activity Value	Durability Metric	Durability Result	Ref.
PtRuNiCoFeMo HEA SNWs on C	HOR	Alkaline electrolyte	Mass activity	6.75 A·mg(Pt + Ru)^−1^	Mass-activity loss after 2000 cycles	6.2%	[199]
Nanoporous AlNiCuPtPdAu HEA	ORR	Half-cell	Relative activity	10 × Pt/C	Activity retention after 100,000 cycles	92.5%	[200]
PtFeCoNiCu HEA	ORR	Half-cell	Mass activity at 0.90 V	1.738 A·mgPt^−1^	—	—	[201]
PtPdFeCoNi HEA nanorings	ORR	0.1 M KOH	Mass activity at 0.95 V	0.99 A·mgPGM^−1^	E_1/2_ loss after 50,000 cycles	10 mV	[202]
PtZnFeCoNiCr antisite-defect HEI	ORR	Half-cell	Mass activity	4.12 A·mgPt^−1^	Potential loss after 30,000 cycles	14 mV	[203]
N-L1_0_-PtCoNiFeCu/KB	ORR	H_2_-air HDV MEA	Current density at 0.70 V	1388 mA·cm^−2^	Voltage loss after 90,000 cycles	9 mV	[204]
FeCoNiCuPt HEA-NPs/graphene	ORR	Half-cell	Mass activity at 0.90 V	1.94 A·mgPt^−1^	Mass-activity loss after 30,000 cycles	5.5%	[205]
FeCoNiCuPt HEA-NPs/graphene	HER	Half-cell	Overpotential at 10 mA·cm^−2^	15 mV	—	—	[205]
FeCoNiCuPt HEA-NPs/graphene	PEMWE	Device	Cell voltage at 1 A·cm^−2^	1.66 V	Stability duration at 1 A·cm^−2^	1000 h	[205]
NiCoFePtRh us-HEA/C	HER	0.5 M H_2_SO_4_	Overpotential at 10 mA·cm^−2^	27 mV	Current retention after 85 h	100%	[206]
FeCoNiMoCr/NF	OER	1 M KOH	Overpotential at 10 mA·cm^−2^	271 mV	Stability duration at 100 mA·cm^−2^	100 h	[207]
FeCoNiCrMn reconstructed film	OER	—	Overpotential at 10 mA·cm^−2^	263 mV	—	—	[208]

An em dash (—) indicates that the value was not reported or was unavailable.

## Data Availability

No new data were created or analyzed in this study. Data sharing is not applicable to this article.

## References

[1] Yeh J.-W., Chen S.-K., Lin S.-J., Gan J.-Y., Chin T.-S., Shun T.-T., Tsau C.-H., Chang S.-Y. (2004). Nanostructured High-Entropy Alloys with Multiple Principal Elements: Novel Alloy Design Concepts and Outcomes. Adv. Eng. Mater..

[2] Cantor B., Chang I.T.H., Knight P., Vincent A.J.B. (2004). Microstructural Development in Equiatomic Multicomponent Alloys. Mater. Sci. Eng. A.

[3] Miracle D.B. (2019). High entropy alloys as a bold step forward in alloy development. Nat. Commun..

[4] Chen W., Hilhorst A., Bokas G., Gorsse S., Jacques P., Hautier G. (2023). A map of single-phase high-entropy alloys. Nat. Commun..

[5] Tracy C.L., Park S., Rittman D.R., Zinkle S.J., Bei H., Lang M., Ewing R.C., Mao W.L. (2017). High pressure synthesis of a hexagonal close-packed phase of the high-entropy alloy CrMnFeCoNi. Nat. Commun..

[6] Rajendrachari S. (2022). An Overview of High-Entropy Alloys Prepared by Mechanical Alloying Followed by the Characterization of Their Microstructure and Various Properties. Alloys.

[7] Karati A., Nagini M., Ghosh S., Shabadi R., Pradeep K.G., Mallik R.C., Murty B.S., Varadaraju U.V. (2019). Ti2NiCoSnSb—A new half-Heusler type high-entropy alloy showing simultaneous increase in Seebeck coefficient and electrical conductivity for thermoelectric applications. Sci. Rep..

[8] Padamata S.K., Yasinskiy A., Yanov V., Saevarsdottir G. (2022). Magnetron Sputtering High-Entropy Alloy Coatings: A Mini-Review. Metals.

[9] Sonal S., Lee J. (2021). Recent Advances in Additive Manufacturing of High Entropy Alloys and Their Nuclear and Wear-Resistant Applications. Metals.

[10] Sveda M., Kristaly F., Sikora E., Sycheva A., Karacs G., Ferenczi T., Janovszky D. (2024). Microstructure evolution and catalytic activity of AlCo1-xFeNiTiMox high entropy alloys fabricated by powder metallurgy route. Sci. Rep..

[11] Cao Z., Zhang P., An B., Li D., Yu Y., Pan J., Zhang C., Liu L. (2024). In situ phase engineering during additive manufacturing enables high-performance soft-magnetic medium-entropy alloys. Nat. Commun..

[12] Ding Q., Zhang Y., Chen X., Fu X., Chen D., Chen S., Gu L., Wei F., Bei H., Gao Y. (2019). Tuning Element Distribution, Structure and Properties by Composition in High-Entropy Alloys. Nature.

[13] Santodonato L.J., Zhang Y., Feygenson M., Parish C.M., Gao M.C., Weber R.J., Neuefeind J.C., Tang Z., Liaw P.K. (2015). Deviation from High-Entropy Configurations in the Atomic Distributions of a Multi-Principal-Element Alloy. Nat. Commun..

[14] Zhang R., Zhao S., Ding J., Chong Y., Jia T., Ophus C., Asta M., Ritchie R.O., Minor A.M. (2020). Short-Range Order and Its Impact on the CrCoNi Medium-Entropy Alloy. Nature.

[15] Chen X., Wang Q., Cheng Z., Zhu M., Zhou H., Jiang P., Zhou L., Xue Q., Yuan F., Zhu J. (2021). Direct Observation of Chemical Short-Range Order in a Medium-Entropy Alloy. Nature.

[16] Woo W., Jeong J.S., Kim D.-K., Lee C.M., Choi S.-H., Suh J.-Y., Lee S.Y., Harjo S., Kawasaki T. (2020). Stacking Fault Energy Analyses of Additively Manufactured Stainless Steel 316L and CrCoNi Medium Entropy Alloy Using In Situ Neutron Diffraction. Sci. Rep..

[17] Zhou R., Chen W., Li W., Chou T.-H., Chen Y.-H., Liang X., Luan J., Zhu Y., Huang J.C., Liu Y. (2023). 3D printed N-doped CoCrFeNi high entropy alloy with more than doubled corrosion resistance in dilute sulphuric acid. npj Mater. Degrad..

[18] Esmaily M., Qiu Y., Bigdeli S., Venkataraman M.B., Allanore A., Birbilis N. (2020). High-temperature oxidation behaviour of AlxFeCrCoNi and AlTiVCr compositionally complex alloys. npj Mater. Degrad..

[19] Gludovatz B., Hohenwarter A., Catoor D., Chang E.H., George E.P., Ritchie R.O. (2014). A Fracture-Resistant High-Entropy Alloy for Cryogenic Applications. Science.

[20] George E.P., Curtin W.A., Tasan C.C. (2020). High Entropy Alloys: A Focused Review of Mechanical Properties and Deformation Mechanisms. Acta Mater..

[21] Brif Y., Thomas M., Todd I. (2015). The Use of High-Entropy Alloys in Additive Manufacturing. Scr. Mater..

[22] Gorr B., Müller F., Azim M., Christ H.-J., Müller T., Chen H., Kauffmann A., Heilmaier M. (2017). High-Temperature Oxidation Behavior of Refractory High-Entropy Alloys: Effect of Alloy Composition. Oxid. Met..

[23] Granberg F., Nordlund K., Ullah M.W., Jin K., Lu C., Bei H., Wang L.M., Djurabekova F., Weber W.J., Zhang Y. (2016). Mechanism of Radiation Damage Reduction in Equiatomic Multicomponent Single Phase Alloys. Phys. Rev. Lett..

[24] Batchelor T.A.A., Pedersen J.K., Winther S.H., Castelli I.E., Jacobsen K.W., Rossmeisl J. (2019). High-Entropy Alloys as a Discovery Platform for Electrocatalysis. Joule.

[25] Krishna S.A., Noble N., Radhika N., Saleh B. (2024). A Comprehensive Review on Advances in High-Entropy Alloys: Fabrication and Surface Modification Methods, Properties, Applications, and Future Prospects. J. Manuf. Process..

[26] Oketola A.M., Adegbola T.A., Jamiru T., Ogunbiyi O., Salifu S. (2025). Advances in High-Entropy Alloy Research: Unraveling Fabrication Techniques, Microstructural Transformations, and Mechanical Properties. J. Bio-Tribo-Corros..

[27] Han F., Li C., Huang J., Wang J., Xue L., Wang C., Zhang Y. (2025). Research Advances in Additively Manufactured High-Entropy Alloys: Microstructure, Mechanical Properties, and Corrosion Resistance. Metals.

[28] Pan W., Huang D., Wang W., Dou G., Lyu P. (2025). Recent Advances in High-Temperature Properties of High-Entropy Alloys. High-Temp. Mater..

[29] Sasi A., Vikram R.J., Dash K. (2025). Corrosion and Oxidation Behavior of High Entropy Alloys in Extreme and Harsh Environments: A Perspective on Steam Corrosion. J. Appl. Phys..

[30] Jeje S.O., Mpofu P., Malatji N., Kanyane L.R., Shongwe M.B. (2025). Multi-Principal Element Alloy Coatings: A Review of Deposition Techniques, Applications, and Future Prospects. Met. Mater. Int..

[31] Li S., Liu X., Hou X., Liu Z., Wang X., Narayanan J.A., Wu T., Bai Y., Dong Y., Jiang H. (2025). Evolution of Corrosion Mechanism of 3d Transition Metal High Entropy Alloys: A Review. J. Mater. Res. Technol..

[32] Jiménez-Arévalo V.M., Martin P., Sepúlveda M.F., Azocar M.I., Zhou X., Galleguillos Madrid F.M., Ramirez C.G., Zagal J.H., Páez M. (2025). Recent Advances in High-Entropy Alloys for Electrocatalysis: From Rational Design to Functional Performance. Mater. Des..

[33] Cao J., Sun J., Li H., Wu Z., An X. (2026). Mastering Complexity toward High-Performance Multi-Principal Element Alloy-Based Films and Coatings: A Review on Microstructural Regulation and Property Optimization. Adv. Sci..

[34] Mooraj S., Kim G., Fan X., Samuha S., Xie Y., Li T., Tiley J.S., Chen Y., Yu D., An K. (2024). Additive Manufacturing of Defect-Free TiZrNbTa Refractory High-Entropy Alloy with Enhanced Elastic Isotropy via In-Situ Alloying of Elemental Powders. Commun. Mater..

[35] Liang Y.-J., Wang L., Wen Y., Cheng B., Wu Q., Cao T., Xiao Q., Xue Y., Sha G., Wang Y. (2018). High-content ductile coherent nanoprecipitates achieve ultrastrong high-entropy alloys. Nat. Commun..

[36] Li Z., Tasan C.C., Springer H., Gault B., Raabe D. (2017). Interstitial atoms enable joint twinning and transformation induced plasticity in strong and ductile high-entropy alloys. Sci. Rep..

[37] Zhang Z., Mao M.M., Wang J., Gludovatz B., Zhang Z., Mao S.X., George E.P., Yu Q., Ritchie R.O. (2015). Nanoscale origins of the damage tolerance of the high-entropy alloy CrMnFeCoNi. Nat. Commun..

[38] Shi P., Ren W., Zheng T., Ren Z., Hou X., Peng J., Hu P., Gao Y., Zhong Y., Liaw P.K. (2019). Enhanced strength–ductility synergy in ultrafine-grained eutectic high-entropy alloys by inheriting microstructural lamellae. Nat. Commun..

[39] Hou Y., Gharbi O., Xie C., Ramírez Rico D.S., Sun F., Turmine M., Ogle K., Vivier V. (2026). Tracking Element-Specific Dissolution during Pitting Corrosion: An Operando ICP-AES–Electrochemical Study of the CoCrFeMnNi Cantor Alloy. npj Mater. Degrad..

[40] El Atwani O., Vo H.T., Tunes M.A., Lee C., Alvarado A., Krienke N., Poplawsky J.D., Kohnert A.A., Gigax J., Chen W.-Y. (2023). A quinary WTaCrVHf nanocrystalline refractory high-entropy alloy withholding extreme irradiation environments. Nat. Commun..

[41] Yang Y.-F., Hu F., Xia T., Li R.-H., Bai J.-Y., Zhu J.-Q., Xu J.-Y., Zhang G.-F. (2025). High Entropy Alloys: A Review of Preparation Techniques, Properties and Industry Applications. J. Alloys Compd..

[42] Han Y., Chen H., Sun Y., Liu J., Wei S., Xie B., Zhang Z., Zhu Y., Li M., Yang J. (2024). Ubiquitous short-range order in multi-principal element alloys. Nat. Commun..

[43] Gorsse S., Miracle D.B., Senkov O.N. (2018). From High-Entropy Alloys to Complex Concentrated Alloys. Comptes Rendus Phys..

[44] Pickering E.J., Jones N.G. (2016). High-entropy alloys: A critical assessment of their founding principles and future prospects. Int. Mater. Rev..

[45] Otto F., Yang Y., Bei H., George E.P. (2013). Relative Effects of Enthalpy and Entropy on the Phase Stability of Equiatomic High-Entropy Alloys. Acta Mater..

[46] Schön C.G., Duong T., Wang Y., Arróyave R. (2018). Probing the Entropy Hypothesis in Highly Concentrated Alloys. Acta Mater..

[47] Feng R., Liaw P.K., Gao M.C., Widom M. (2017). First-Principles Prediction of High-Entropy-Alloy Stability. npj Comput. Mater..

[48] Lederer Y., Toher C., Vecchio K.S., Curtarolo S. (2018). The Search for High Entropy Alloys: A High-Throughput Ab-Initio Approach. Acta Mater..

[49] Miracle D.B., Senkov O.N. (2017). A Critical Review of High Entropy Alloys and Related Concepts. Acta Mater..

[50] Santodonato L.J., Liaw P.K., Unocic R.R., Bei H., Morris J.R. (2018). Predictive multiphase evolution in Al-containing high-entropy alloys. Nat. Commun..

[51] Yang X., Zhang Y. (2012). Prediction of High-Entropy Stabilized Solid-Solution in Multi-Component Alloys. Mater. Chem. Phys..

[52] Senkov O., Miller J., Miracle D., Woodward C. (2015). Accelerated exploration of multi-principal element alloys with solid solution phases. Nat. Commun..

[53] Wang Y.Z., Wang Y.J. (2022). Disentangling diffusion heterogeneity in high-entropy alloys. Acta Mater..

[54] Andreoli A.F., Shuleshova O., Witusiewicz V.T., Wu Y., Yang Y., Ivashko O., Dippel A.C., Zimmermann M.V., Nielsch K., Kaban I. (2021). In situ study of non-equilibrium solidification of CoCrFeNi high-entropy alloy and CrFeNi and CoCrNi ternary suballoys. Acta Mater..

[55] Tsai K.-Y., Tsai M.-H., Yeh J.-W. (2013). Sluggish Diffusion in Co-Cr-Fe-Mn-Ni High-Entropy Alloys. Acta Mater..

[56] Li Q., Chen W., Zhong J., Zhang L., Chen Q., Liu Z.K. (2017). On Sluggish Diffusion in Fcc Al–Co–Cr–Fe–Ni High-Entropy Alloys: An Experimental and Numerical Study. Metals.

[57] Mehta A., Sohn Y. (2021). Investigation of sluggish diffusion in FCC Al0.25CoCrFeNi high-entropy alloy. Mater. Res. Lett..

[58] Vaidya M., Pradeep K.G., Murty B.S., Wilde G., Divinski S.V. (2018). Bulk Tracer Diffusion in CoCrFeNi and CoCrFeMnNi High Entropy Alloys. Acta Mater..

[59] Gaertner D., Abrahams K., Kottke J., Esin V.A., Steinbach I., Wilde G., Divinski S.V. (2019). Concentration-Dependent Atomic Mobilities in FCC CoCrFeMnNi High-Entropy Alloys. Acta Mater..

[60] Kottke J., Laurent-Brocq M., Fareed A., Gaertner D., Perrière L., Rogal Ł., Divinski S.V., Wilde G. (2019). Tracer Diffusion in the Ni–CoCrFeMn System: Transition from a Dilute Solid Solution to a High Entropy Alloy. Scr. Mater..

[61] Vaidya M., Pradeep K.G., Murty B.S., Wilde G., Divinski S.V. (2017). Radioactive isotopes reveal a non sluggish kinetics of grain boundary diffusion in high entropy alloys. Sci. Rep..

[62] Glienke M., Vaidya M., Gururaj K., Daum L., Tas B., Rogal Ł., Pradeep K.G., Divinski S.V., Wilde G. (2020). Grain Boundary Diffusion in CoCrFeMnNi High Entropy Alloy: Kinetic Hints towards a Phase Decomposition. Acta Mater..

[63] Dąbrowa J., Danielewski M. (2020). State-of-the-Art Diffusion Studies in the High Entropy Alloys. Metals.

[64] Dąbrowa J., Zajusz M., Kucza W., Cieślak G., Berent K., Czeppe T., Kulik T., Danielewski M. (2019). Demystifying the Sluggish Diffusion Effect in High Entropy Alloys. J. Alloys Compd..

[65] Biswas K., Yeh J.-W., Bhattacharjee P.P., De Hosson J.T.M. (2020). High Entropy Alloys: Key Issues under Passionate Debate. Scr. Mater..

[66] Owen L.R., Pickering E.J., Playford H.Y., Stone H.J., Tucker M.G., Jones N.G. (2017). An Assessment of the Lattice Strain in the CrMnFeCoNi High-Entropy Alloy. Acta Mater..

[67] Ye Y.F., Zhang Y.H., He Q.F., Zhuang Y., Wang S., Shi S., Hu A., Fan J., Yang Y. (2018). Atomic-Scale Distorted Lattice in Chemically Disordered Equimolar Complex Alloys. Acta Mater..

[68] Cao B.X., Wang C., Yang T., Liu C.T. (2020). Cocktail Effects in Understanding the Stability and Properties of Face-Centered-Cubic High-Entropy Alloys at Ambient and Cryogenic Temperatures. Scr. Mater..

[69] Long Y., Li G., Liang X., Peng H. (2020). Fine-Grained FeCoNi(CuAl)x High Entropy Alloys: Phase Transformation, Microstructure Evolution and Mechanical Properties. Front. Mater..

[70] Shahmir H., Mehranpour M.S., Arsalan Shams S.A., Langdon T.G. (2023). Twenty years of the CoCrFeNiMn high-entropy alloy: Achieving exceptional mechanical properties through microstructure engineering. J. Mater. Res. Technol..

[71] Zeng Y., Man M., Bai K., Zhang Y.-W. (2021). Revealing high-fidelity phase selection rules for high entropy alloys: A combined CALPHAD and machine learning study. Mater. Des..

[72] Kim G., Diao H., Lee C., Samaei A.T., Phan T., de Jong M., An K., Ma D., Liaw P.K., Chen W. (2019). First-Principles and Machine Learning Predictions of Elasticity in Severely Lattice-Distorted High-Entropy Alloys with Experimental Validation. Acta Mater..

[73] Fourmont A., Le Gallet S., Politano O., Desgranges C., Baras F. (2020). Effects of Planetary Ball Milling on AlCoCrFeNi High Entropy Alloys Prepared by Spark Plasma Sintering: Experiments and Molecular Dynamics Study. J. Alloys Compd..

[74] Li J.L., Li Z., Wang Q., Dong C., Liaw P.K. (2020). Phase-Field Simulation of Coherent BCC/B2 Microstructures in High Entropy Alloys. Acta Mater..

[75] Pei Z., Yin J., Hawk J.A., Alman D.E., Gao M.C. (2020). Machine-Learning Informed Prediction of High-Entropy Solid Solution Formation: Beyond the Hume-Rothery Rules. npj Comput. Mater..

[76] Sun Y., Lu Z., Liu X., Du Q., Xie H., Lv J., Song R., Wu Y., Wang H., Jiang S. (2021). Prediction of Ti-Zr-Nb-Ta High-Entropy Alloys with Desirable Hardness by Combining Machine Learning and Experimental Data. Appl. Phys. Lett..

[77] Rao Z., Tung P.-Y., Xie R., Wei Y., Zhang H., Ferrari A., Klaver T., Körmann F., Sukumar P.T., da Silva A.K. (2022). Machine Learning-Enabled High-Entropy Alloy Discovery. Science.

[78] Singh N., Shadangi Y., Shivam V., Mukhopadhyay N.K. (2021). MgAlSiCrFeNi Low-Density High Entropy Alloy Processed by Mechanical Alloying and Spark Plasma Sintering: Effect on Phase Evolution and Thermal Stability. J. Alloys Compd..

[79] Abbas M.A., Sadek W.M., Ibrahim S.A. (2024). Effect of Milling Time on the Structure Stability of FeMnNiCrAl Non-equiatomic High-Entropy Alloy. Trans. Indian Inst. Met..

[80] Moravcik I., Kubicek A., Moravcikova-Gouvea L., Adam O., Kana V., Pouchly V., Zadera A., Dlouhy I. (2020). The Origins of High-Entropy Alloy Contamination Induced by Mechanical Alloying and Sintering. Metals.

[81] Jain H., Shadangi Y., Chakravarty D., Dubey A.K., Mukhopadhyay N.K. (2022). High Entropy Steel Processed through Mechanical Alloying and Spark Plasma Sintering: Alloying Behaviour, Thermal Stability and Mechanical Properties. Mater. Sci. Eng. A.

[82] Gwalani B., Pohan R.M., Lee J., Banerjee R., Ryu H.J., Hong S.H. (2018). High-Entropy Alloy Strengthened by In Situ Formation of Entropy-Stabilized Nano-Dispersoids. Sci. Rep..

[83] Praveen S., Basu J., Kashyap S., Kottada R.S. (2016). Exceptional Resistance to Grain Growth in Nanocrystalline CoCrFeNi High Entropy Alloy at High Homologous Temperatures. J. Alloys Compd..

[84] Yurkova A.I., Cherniavsky V.V., Bolbut V., Krüger M., Bogomol I. (2019). Structure Formation and Mechanical Properties of the High-Entropy AlCuNiFeCr Alloy Prepared by Mechanical Alloying and Spark Plasma Sintering. J. Alloys Compd..

[85] Kumar A., Singh A., Suhane A. (2022). Mechanically alloyed high entropy alloys: Existing challenges and opportunities. J. Mater. Res. Technol..

[86] Hinte C., Fantin A., Barienti K., Herbst S., Frenzel J., Eggeler G., Maier H.J. (2024). A Comparative Study on Arc- and Vacuum Induction-Melting for Ti16.6Zr16.6Hf16.6Co10Ni20Cu20 High Entropy Shape Memory Alloy Production. Discov. Mater..

[87] Lin C., Shiue R.-K., Wu S.-K., Huang H.-L. (2019). Infrared Brazing of CoCrFeMnNi Equiatomic High Entropy Alloy Using Nickel-Based Braze Alloys. Entropy.

[88] Wang Y., Li Y., Wang W., Kong H., Wang Q., Park J.H., Mu W. (2023). Effect of Manufacturing Conditions and Al Addition on Inclusion Characteristics in Co-Based Dual-Phase High Entropy Alloy. Metall. Mater. Trans. A.

[89] Nagase T., Mizuuchi K., Nakano T. (2019). Solidification Microstructures of the Ingots Obtained by Arc Melting and Cold Crucible Levitation Melting in TiNbTaZr Medium-Entropy Alloy and TiNbTaZrX (X = V, Mo, W) High-Entropy Alloys. Entropy.

[90] Wang Y.P., Li B.S., Ren M.X., Yang C., Fu H.J. (2008). Microstructure and Compressive Properties of AlCrFeCoNi High Entropy Alloy. Mater. Sci. Eng. A.

[91] Poulia A., Georgatis E., Mathiou C., Karantzalis A.E. (2018). Phase Segregation Discussion in a Hf25Zr30Ti20Nb15V10 High Entropy Alloy: The Effect of the High Melting Point Element. Mater. Chem. Phys..

[92] Hoi K.C., Lei W.H., Liu Y., Shek C., Ferreira J.T., Cortez N.F., Kwok C., Sun Y., Cristino V.A., Lo K. (2023). Cavitation Erosion of the CoCrFeNi High Entropy Alloy Having Elemental Segregation. Wear.

[93] Li B., Zhang Z., Luo X., Chen K., Zhang J., Gong P., Peng Z. (2024). Microstructure and Friction Properties of AlCrTiVNbx High-Entropy Alloys via Annealing Manufactured by Vacuum Arc Melting. Materials.

[94] Senkov O., Isheim D., Seidman D., Pilchak A. (2016). Development of a Refractory High Entropy Superalloy. Entropy.

[95] Soni V., Senkov O.N., Gwalani B., Miracle D.B., Banerjee R. (2018). Microstructural Design for Improving Ductility of An Initially Brittle Refractory High Entropy Alloy. Sci. Rep..

[96] Heczel A., Kawasaki M., Lábár J.L., Jang J.-I., Langdon T.G., Gubicza J. (2017). Defect Structure and Hardness in Nanocrystalline CoCrFeMnNi High-Entropy Alloy Processed by High-Pressure Torsion. J. Alloys Compd..

[97] Schuh B., Mendez-Martin F., Völker B., George E.P., Clemens H., Pippan R., Hohenwarter A. (2015). Mechanical Properties, Microstructure and Thermal Stability of a Nanocrystalline CoCrFeMnNi High-Entropy Alloy after Severe Plastic Deformation. Acta Mater..

[98] Shahmir H., Mousavi T., He J., Lu Z., Kawasaki M., Langdon T.G. (2017). Microstructure and Properties of a CoCrFeNiMn High-Entropy Alloy Processed by Equal-Channel Angular Pressing. Mater. Sci. Eng. A.

[99] Skrotzki W., Pukenas A., Odor E., Joni B., Ungár T., Völker B., Hohenwarter A., Pippan R., George E.P. (2020). Microstructure, Texture, and Strength Development during High-Pressure Torsion of CrMnFeCoNi High-Entropy Alloy. Crystals.

[100] Dolique V., Thomann A.-L., Brault P., Tessier Y., Gillon P. (2009). Complex Structure/Composition Relationship in Thin Films of AlCoCrCuFeNi High Entropy Alloy. Mater. Chem. Phys..

[101] Braeckman B.R., Boydens F., Hidalgo H., Dutheil P., Jullien M., Thomann A.-L., Depla D. (2015). High Entropy Alloy Thin Films Deposited by Magnetron Sputtering of Powder Targets. Thin Solid Films.

[102] An Z., Jia H., Wu Y., Rack P.D., Patchen A.D., Liu Y., Liu Y., Ren Y., Li N., Liaw P.K. (2015). Solid-Solution CrCoCuFeNi High-Entropy Alloy Thin Films Synthesized by Sputter Deposition. Mater. Res. Lett..

[103] Zendejas Medina L., Riekehr L., Jansson U. (2020). Phase Formation in Magnetron Sputtered CrMnFeCoNi High Entropy Alloy. Surf. Coat. Technol..

[104] Liang Y., Wang P., Wang Y., Dai Y., Hu Z., Tranca D.E., Hristu R., Stanciu S.G., Toma A., Stanciu G.A. (2019). Growth Mechanisms and the Effects of Deposition Parameters on the Structure and Properties of High Entropy Film by Magnetron Sputtering. Materials.

[105] Zhong J.-Y., Wang J.-J., Ouyang F.-Y. (2022). Optimization of Sputtering Process for Medium Entropy Alloy Nanotwinned CoCrFeNi Thin Films by Taguchi Method. Materials.

[106] Liu X., Cai W., Zhang Y., Wang L., Wang J. (2023). Tuning Microstructure and Mechanical and Wear Resistance of ZrNbTiMo Refractory High-Entropy Alloy Films via Sputtering Power. Front. Mater..

[107] Nagy P., Rohbeck N., Roussely G., Sortais P., Lábár J., Gubicza J., Michler J., Pethö L. (2020). Processing and Characterization of a Multibeam Sputtered Nanocrystalline CoCrFeNi High-Entropy Alloy Film. Surf. Coat. Technol..

[108] Dolique V., Thomann A.L., Brault P., Tessier Y., Gillon P. (2010). Thermal stability of AlCoCrCuFeNi high entropy alloy thin films studied by in-situ XRD analysis. Surf. Coat. Technol..

[109] Liu Y., Ren J., Guan S., Li C., Zhang Y., Muskeri S., Liu Z., Yu D., Chen Y., An K. (2023). Microstructure and Mechanical Behavior of Additively Manufactured CoCrFeMnNi High-Entropy Alloys: Laser Directed Energy Deposition versus Powder Bed Fusion. Acta Mater..

[110] Taghian M., Pilehvar Meibody A., Saboori A., Iuliano L. (2025). Challenges and opportunities in additive manufacturing of high entropy alloys. J. Alloys Compd..

[111] Gorsse S., Hutchinson C., Gouné M., Banerjee R. (2017). Additive manufacturing of metals: A brief review of the characteristic microstructures and properties of steels, Ti-6Al-4V and high-entropy alloys. Sci. Technol. Adv. Mater..

[112] Ewald S., Kies F., Hermsen S., Voshage M., Haase C., Schleifenbaum J.H. (2019). Rapid Alloy Development of Extremely High-Alloyed Metals Using Powder Blends in Laser Powder Bed Fusion. Materials.

[113] Zhang H., Zhao Y., Huang S., Zhu S., Wang F., Li D. (2019). Manufacturing and Analysis of High-Performance Refractory High-Entropy Alloy via Selective Laser Melting (SLM). Materials.

[114] Piglione A., Dovgyy B., Liu C., Gourlay C.M., Hooper P.A., Pham M.S. (2018). Printability and microstructure of the CoCrFeMnNi high-entropy alloy fabricated by laser powder bed fusion. Mater. Lett..

[115] Chew Y., Bi G.J., Zhu Z.G., Ng F.L., Weng F., Liu S.B., Nai S.M.L., Lee B.Y. (2019). Microstructure and Enhanced Strength of Laser Aided Additive Manufactured CoCrFeNiMn High Entropy Alloy. Mater. Sci. Eng. A.

[116] Guan S., Wan D., Solberg K., Berto F., Welo T., Yue T.M., Chan K.C. (2019). Additive Manufacturing of Fine-Grained and Dislocation-Populated CrMnFeCoNi High Entropy Alloy by Laser Engineered Net Shaping. Mater. Sci. Eng. A.

[117] Chesetti A., Banerjee S., Dasari S., Varahabhatla S., Sharma A., Mantri S.A., Nartu M.S.K.K.Y., Dahotre N., Banerjee R. (2023). Laser Powder Bed Fusion Processing of a Precipitation Strengthenable FCC Based High Entropy Alloy. Addit. Manuf. Lett..

[118] Yamanaka K., Shiratori H., Mori M., Omura K., Fujieda T., Kuwabara K., Chiba A. (2020). Corrosion mechanism of an equimolar AlCoCrFeNi high-entropy alloy additively manufactured by electron beam melting. npj Mater. Degrad..

[119] Zhang C., Feng K., Kokawa H., Han B., Li Z. (2020). Cracking Mechanism and Mechanical Properties of Selective Laser Melted CoCrFeMnNi High Entropy Alloy Using Different Scanning Strategies. Mater. Sci. Eng. A.

[120] Mooraj S., Dong X., Zhang S., Zhang Y., Ren J., Guan S., Li C., Naorem R., Argibay N., Chen W. (2024). Crack Mitigation in Additively Manufactured AlCrFe2Ni2 High-Entropy Alloys through Engineering Phase Transformation Pathway. Commun. Mater..

[121] Ron T., Leon A., Bassis M., Chen Z., Shirizly A., Aghion E. (2025). Effect of Hot Isostatic Pressing Treatment on Refractory High-Entropy Alloy WTaMoNbV Produced by Laser Powder Bed Fusion Process. Metals.

[122] Li H.G., Lee T.L., Zheng W., Lu Y.Z., Yin H.B.C., Yang J., Huang Y.J., Sun J.F. (2020). Characterization of Residual Stress in Laser Melting Deposited CoCrFeMnNi High Entropy Alloy by Neutron Diffraction. Mater. Lett..

[123] Sun Z., Tan X., Wang C., Descoins M., Mangelinck D., Tor S.B., Jägle E.A., Zaefferer S., Raabe D. (2021). Reducing Hot Tearing by Grain Boundary Segregation Engineering in Additive Manufacturing: Example of an AlxCoCrFeNi High-Entropy Alloy. Acta Mater..

[124] Ahsan M.R.U., Seo G.-J., Fan X., Liaw P.K., Motaman S., Haase C., Kim D.B. (2021). Effects of Process Parameters on Bead Shape, Microstructure, and Mechanical Properties in Wire + Arc Additive Manufacturing of Al0.1CoCrFeNi High-Entropy Alloy. J. Manuf. Process..

[125] Tran A.-T., Khoa N.A., Nguyen M.T., Van Khai T., Quang V.A., Tam L.H., Phuc N.H.H. (2025). Influence of the Ball-to-Powder Ratio on Phase Formation and Functional Properties of AlCoFeNiTi High-Entropy Alloys Synthesized by High-Energy Ball Milling. ACS Omega.

[126] Yazdani N., Toroghinejad M.R., Shabani A., Cavaliere P. (2021). Effects of Process Control Agent Amount, Milling Time, and Annealing Heat Treatment on the Microstructure of AlCrCuFeNi High-Entropy Alloy Synthesized through Mechanical Alloying. Metals.

[127] Wang J., Li T., Xu X., Yang Q., Wang C., Zhan L., Zhou T., Wu T., Zhang M., Wu J. (2026). Microstructural evolution and mechanical properties of Al0.25CrFeNiTi0.25 high-entropy alloy prepared by mechanical alloying and spark plasma sintering. Powder Technol..

[128] Walsh F., Zhang M., Ritchie R.O., Asta M., Minor A.M. (2024). Multiple Origins of Extra Electron Diffractions in FCC Metals. Sci. Adv..

[129] Wang H., Yang P.Y., Zhao W.J., Ma S.H., Hou J.H., He Q.F., Wu C.L., Chen H.A., Wang Q., Cheng Q. (2024). Lattice Distortion Enabling Enhanced Strength and Plasticity in High Entropy Intermetallic Alloy. Nat. Commun..

[130] Hsiao H.-W., Feng R., Ni H., An K., Poplawsky J.D., Liaw P.K., Zuo J.-M. (2022). Data-Driven Electron-Diffraction Approach Reveals Local Short-Range Ordering in CrCoNi with Ordering Effects. Nat. Commun..

[131] He Q.F., Tang P.H., Chen H.A., Lan S., Wang J.G., Luan J.H., Du M., Liu Y., Liu C.T., Pao C.W. (2021). Understanding Chemical Short-Range Ordering/Demixing Coupled with Lattice Distortion in Solid Solution High Entropy Alloys. Acta Mater..

[132] Bacurau V.P., Moreira P.A.F.P., Bertoli G., Andreoli A.F., Mazzer E., de Assis F.F., Gargarella P., Koga G., Stumpf G.C., Figueroa S.J.A. (2024). Comprehensive Analysis of Ordering in CoCrNi and CrNi2 Alloys. Nat. Commun..

[133] Sharma A., Jiang C., Gwalani B., Lin W.C., Lo K.C., Gorsse S., Yeh A.C., Srinivasan S.G., Banerjee R., Dasari S. (2023). Exceptional Enhancement of Mechanical Properties in High-Entropy Alloys via Thermodynamically Guided Local Chemical Ordering. Proc. Natl. Acad. Sci. USA.

[134] Moniri S., Yang Y., Ding J., Yuan Y., Zhou J., Yang L., Zhu F., Liao Y., Yao Y., Hu L. (2023). Three-Dimensional Atomic Structure and Local Chemical Order of Medium- and High-Entropy Nanoalloys. Nature.

[135] Li Y., Wei Y., Wang Z., Liu X., Colnaghi T., Han L., Rao Z., Zhou X., Huber L., Dsouza R. (2023). Quantitative Three-Dimensional Imaging of Chemical Short-Range Order via Machine Learning Enhanced Atom Probe Tomography. Nat. Commun..

[136] Wang Z., Gu J., An D., Liu Y., Song M. (2020). Characterization of the Microstructure and Deformation Substructure Evolution in a Hierarchal High-Entropy Alloy by Correlative EBSD and ECCI. Intermetallics.

[137] Wang F., An X., Wang Z., Wu W., Xia W., Ni S., Gu J., Yi J., Yang Y., Song M. (2025). Enhanced Strength and Ductility of High-Entropy Alloy via Dislocation-Mediated Heterogeneous Martensitic Transformation. Microstructures.

[138] Chen Z., Zhu W., Wang H., He Q., Fang Q., Liu X., Li J., Yang Y. (2024). Overcoming Strength–Toughness Trade-Off in a Eutectic High Entropy Alloy by Optimizing Chemical and Microstructural Heterogeneities. Commun. Mater..

[139] Ye Y.F., Wang Q., Zhao Y.L., He Q.F., Lu J., Yang Y. (2016). Elemental Segregation in Solid-Solution High-Entropy Alloys: Experiments and Modeling. J. Alloys Compd..

[140] Su Z., Ding J., Song M., Jiang L., Shi T., Li Z., Wang S., Gao F., Yun D., Ma E. (2023). Enhancing the Radiation Tolerance of High-Entropy Alloys via Solute-Promoted Chemical Heterogeneities. Acta Mater..

[141] Qiu Y., Liu R., Gengenbach T., Gharbi O., Choudhary S., Thomas S., Fraser H.L., Birbilis N. (2020). Real-Time Dissolution of a Compositionally Complex Alloy Using Inline ICP and Correlation with XPS. npj Mater. Degrad..

[142] Wang X., Mercier D., Zanna S., Seyeux A., Perriere L., Laurent-Brocq M., Guillot I., Maurice V., Marcus P. (2023). Origin of Enhanced Passivity of Cr-Fe-Co-Ni-Mo Multi-Principal Element Alloy Surfaces. npj Mater. Degrad..

[143] Wang Z., Liu Z.-X., Jin J., Tang D.-Z., Zhang L. (2023). Selective Corrosion Mechanism of CoCrFeMoNi High-Entropy Alloy in the Transpassive Region Based on the Passive Film Characterization by ToF-SIMS. Corros. Sci..

[144] Yu X., Chen Q., Cui X., Ouyang D. (2025). Nb-Induced Lattice Changes to Enhance Corrosion Resistance of Al0.5Ti3Zr0.5NbxMo0.2 High-Entropy Alloys. Nat. Commun..

[145] Wang L., Mercier D., Zanna S., Seyeux A., Laurent-Brocq M., Perrière L., Guillot I., Marcus P. (2020). Study of the Surface Oxides and Corrosion Behaviour of an Equiatomic CoCrFeMnNi High Entropy Alloy by XPS and ToF-SIMS. Corros. Sci..

[146] Wetzel A., Hans A.-K., von der Au M., Brand I., Wittstock G., Ozcan O., Witt J. (2025). Long-Term Corrosion Studies of CrCoNi and CrMnFeCoNi in Sulfuric Acid. npj Mater. Degrad..

[147] Wang L., Zanna S., Mercier D., Maurice V., Marcus P. (2023). Early-Stage Surface Oxidation of the Equiatomic CoCrFeMnNi High Entropy Alloy Studied In Situ by XPS. Corros. Sci..

[148] Wang L., Seyeux A., Perrière L., Mercier D., Maurice V., Marcus P. (2021). Insight on Passivity of High Entropy Alloys: Thermal Stability and Ion Transport Mechanisms in the Passive Oxide Film on CoCrFeMnNi Surfaces. Corros. Sci..

[149] Zhang F., Wu Y., Lou H., Zeng Z., Prakapenka V.B., Greenberg E., Ren Y., Yan J., Okasinski J.S., Liu X. (2017). Polymorphism in a high-entropy alloy. Nat. Commun..

[150] Ma L., Wang L., Nie Z., Wang F., Xue Y., Zhou J., Cao T., Wang Y., Ren Y. (2017). Reversible Deformation-Induced Martensitic Transformation in Al0.6CoCrFeNi High-Entropy Alloy Investigated by In Situ Synchrotron-Based High-Energy X-Ray Diffraction. Acta Mater..

[151] Shen J., Lopes J.G., Zeng Z., Choi Y.T., Maawad E., Schell N., Kim H.S., Mishra R.S., Oliveira J. (2023). Deformation Behavior and Strengthening Effects of an Eutectic AlCoCrFeNi2.1 High Entropy Alloy Probed by In-Situ Synchrotron X-Ray Diffraction and Post-Mortem EBSD. Mater. Sci. Eng. A.

[152] Chen S., Oh H.S., Gludovatz B., Kim S.J., Park E.S., Zhang Z., Ritchie R.O., Yu Q. (2020). Real-time observations of TRIP-induced ultrahigh strain hardening in a dual-phase CrMnFeCoNi high-entropy alloy. Nat. Commun..

[153] Ma Y., Qiu K., Xu N., Yang C., Zhang J., Zhang Z., Deng Q., Yue Y., Wang L., Han X. (2023). In-Situ Atomic-Scale Observation of Dislocation and Deformation Twin Interaction in FeMnCoCr High-Entropy Alloy. Mater. Res. Lett..

[154] Arfaoui M., Radnóczi G., Kovács Kis V. (2020). Transformations in CrFeCoNiCu High Entropy Alloy Thin Films during In-Situ Annealing in TEM. Coatings.

[155] Rao J.C., Diao H.Y., Ocelík V., Vainchtein D., Zhang C., Kuo C., Tang Z., Guo W., Poplawsky J., Zhou Y. (2017). Secondary phases in AlxCoCrFeNi high-entropy alloys: An in-situ TEM heating study and thermodynamic appraisal. Acta Mater..

[156] Zhang W., Shen J., Oliveira J.P., Wang H., Feng S., Schell N., Kooi B.J., Pei Y. (2023). Deformation Processes of Additively Manufactured Interstitial-Strengthened High Entropy Alloy: In-Situ High-Energy Synchrotron X-Ray Diffraction and Microstructural Appraisal. Addit. Manuf..

[157] Bu Y., Wu Y., Lei Z., Yuan X., Liu L., Wang P., Liu X., Wu H., Liu J., Wang H. (2024). Elastic Strain-Induced Amorphization in High-Entropy Alloys. Nat. Commun..

[158] Li Z., Pradeep K.G., Deng Y., Raabe D., Tasan C.C. (2016). Metastable High-Entropy Dual-Phase Alloys Overcome the Strength–Ductility Trade-Off. Nature.

[159] Gao S., Ji W., Zhu Q., Jarlöv A., Bai X., Shen X., Liu Y., Nai M.L.S., Gao H., Zhou K. (2025). Unveiling the Mechanisms of Strength–Ductility Synergy in an Additively Manufactured Nanolamellar High-Entropy Alloy. Nat. Commun..

[160] Kumar P., Huang S., Cook D.H., Chen K., Ramamurty U., Tan X., Ritchie R.O. (2024). A strong fracture-resistant high-entropy alloy with nano-bridged honeycomb microstructure intrinsically toughened by 3D-printing. Nat. Commun..

[161] Gludovatz B., Hohenwarter A., Thurston K.V.S., Bei H., Wu Z., George E.P., Ritchie R.O. (2016). Exceptional Damage-Tolerance of a Medium-Entropy Alloy CrCoNi at Cryogenic Temperatures. Nat. Commun..

[162] Lam T.-N., Lee S.Y., Tsou N.-T., Chou H.-S., Lai B.-H., Chang Y.-J., Feng R., Kawasaki T., Harjo S., Liaw P.K. (2020). Enhancement of Fatigue Resistance by Overload-Induced Deformation Twinning in a CoCrFeMnNi High-Entropy Alloy. Acta Mater..

[163] Feng R., Rao Y., Liu C., Xie X., Yu D., Chen Y., Ghazisaeidi M., Ungar T., Wang H., An K. (2021). Enhancing fatigue life by ductile-transformable multicomponent B2 precipitates in a high-entropy alloy. Nat. Commun..

[164] Qiu J., Lu T., Yao N., Chen X., Li K., Sun B., Chen Y., Zhang X.-C., Tu S.-T. (2024). New Insights into Fatigue Anisotropy of an Additively Manufactured Medium-Entropy Alloy: From the Perspectives of Crack Initiation and Crack Propagation. Virtual Phys. Prototyp..

[165] Shi Y., Yang B., Xie X., Brechtl J., Dahmen K.A., Liaw P.K. (2017). Corrosion of AlxCoCrFeNi High-Entropy Alloys: Al-Content and Potential Scan-Rate Dependent Pitting Behavior. Corros. Sci..

[166] Nascimento C.B., Donatus U., Ríos C.T., Antunes R.A. (2021). Passive Film Composition and Stability of CoCrFeNi and CoCrFeNiAl High Entropy Alloys in Chloride Solution. Mater. Chem. Phys..

[167] Zhao X., Huang H., Su Y., Qiao L., Yan Y. (2024). Exploring High Corrosion-Resistant Refractory High-Entropy Alloy via a Combined Experimental and Simulation Study. npj Mater. Degrad..

[168] Zhang M., Shi X., Li Z., Xu H., Li G. (2022). Corrosion Behaviors and Mechanism of CrFeNi2 Based High-Entropy Alloys. Corros. Sci..

[169] Lou Y., Dai C., Chang W., Qian H., Huang L., Du C., Zhang D. (2020). Microbiologically influenced corrosion of FeCoCrNiMo0.1 high-entropy alloys by marine Pseudomonas aeruginosa. Corros. Sci..

[170] Kim Y.-K., Joo Y.A., Kim H.S., Lee K.-A. (2018). High Temperature Oxidation Behavior of Cr-Mn-Fe-Co-Ni High Entropy Alloy. Intermetallics.

[171] Lo K.-C., Chang Y.-J., Murakami H., Yeh J.-W., Yeh A.-C. (2019). An oxidation resistant refractory high entropy alloy protected by CrTaO4-based oxide. Sci. Rep..

[172] Gwalani B., Martin A., Kautz E., Guo B., Lambeets S.V., Olszta M., Battu A.K., Malakar A., Yang F., Guo J. (2024). Mechanistic understanding of speciated oxide growth in high entropy alloys. Nat. Commun..

[173] Arshad M., Bano S., Amer M., Janik V., Hayat Q., Huang Y., Guan D., Bai M. (2023). Thermodynamic Insights into the Oxidation Mechanisms of CrMnFeCoNi High-Entropy Alloy Using In Situ X-ray Diffraction. Materials.

[174] Arshad M., Bano S., Amer M., Janik V., Hayat Q., Bai M. (2024). High-Temperature Oxidation and Phase Stability of AlCrCoFeNi High Entropy Alloy: Insights from In Situ HT-XRD and Thermodynamic Calculations. Materials.

[175] Gorr B., Schellert S., Müller F., Christ H.-J., Kauffmann A., Heilmaier M. (2021). Current Status of Research on the Oxidation Behavior of Refractory High Entropy Alloys. Adv. Eng. Mater..

[176] Lu J., Li L., Zhang H., Chen Y., Luo L., Zhao X., Guo F., Xiao P. (2021). Oxidation Behavior of Gas-Atomized AlCoCrFeNi High-Entropy Alloy Powder at 900–1100 °C. Corros. Sci..

[177] Gaweł R., Rogal Ł., Dąbek J., Wójcik-Bania M., Przybylski K. (2021). High Temperature Oxidation Behaviour of Non-Equimolar AlCoCrFeNi High Entropy Alloys. Vacuum.

[178] Lin W.-C., Lien Y.-W., Moreau L.E., Murakami H., Lo K.-C., Gorsse S., Yeh A.-C. (2024). High-Temperature Oxidation of NbTi-Bearing Refractory Medium- and High-Entropy Alloys. Materials.

[179] Samoilova O., Pratskova S., Shaburova N., Ostovari Moghaddam A., Trofimov E. (2025). High-Temperature Oxidation Resistance of Fe-Free AlCoCrNiNb0.2 and AlCoCr0.5NiNb0.2 High-Entropy Alloys. Materials.

[180] Tan X., Trehern W., Sundar A., Wang Y., San S., Lu T., Zhou F., Sun T., Zhang Y., Wen Y. (2025). Machine Learning and High-Throughput Computational Guided Development of High Temperature Oxidation-Resisting Ni-Co-Cr-Al-Fe Based High-Entropy Alloys. npj Comput. Mater..

[181] Lin Y., Yang T., Lang L., Shan C., Deng H., Hu W., Gao F. (2020). Enhanced Radiation Tolerance of the Ni-Co-Cr-Fe High-Entropy Alloy as Revealed from Primary Damage. Acta Mater..

[182] Lu C., Niu L., Chen N., Jin K., Yang T., Xiu P., Zhang Y., Gao F., Bei H., Shi S. (2016). Enhancing Radiation Tolerance by Controlling Defect Mobility and Migration Pathways in Multicomponent Single-Phase Alloys. Nat. Commun..

[183] Tuomisto F., Makkonen I., Heikinheimo J., Granberg F., Djurabekova F., Nordlund K., Velisa G., Bei H., Xue H., Weber W. (2020). Segregation of Ni at Early Stages of Radiation Damage in NiCoFeCr Solid Solution Alloys. Acta Mater..

[184] Zhang Z., Su Z., Zhang B., Yu Q., Ding J., Shi T., Lu C., Ritchie R.O., Ma E. (2023). Effect of local chemical order on the irradiation-induced defect evolution in CrCoNi medium-entropy alloy. Proc. Natl. Acad. Sci. USA.

[185] Yang T., Guo W., Poplawsky J.D., Li D., Wang L., Li Y., Hu W., Crespillo M.L., Yan Z., Zhang Y. (2020). Structural damage and phase stability of Al0.3CoCrFeNi high entropy alloy under high temperature ion irradiation. Acta Mater..

[186] Esfandiarpour A., Kalita D., Kozioł Z., Alava M. (2025). Comparative Study on Radiation Resistance of WTaCrV High-Entropy Alloy and Tungsten in Helium-Containing Conditions. Sci. Rep..

[187] Su Z., Shi T., Yang J., Shen H., Li Z., Wang S., Ran G., Lu C. (2022). The Effect of Interstitial Carbon Atoms on Defect Evolution in High Entropy Alloys under Helium Irradiation. Acta Mater..

[188] Nori S.T., Ferreiros P.A., Kalita D., Bjørge R., Vullum P.E., Mulewska K., Chrominski W., Li M., Chang Y., Zhang Y. (2025). Nanostructured NiCoFeCr alloy with superior high-temperature irradiation resistance. npj Mater. Degrad..

[189] Pickering E.J., Carruthers A.W., Barron P.J., Middleburgh S.C., Armstrong D.E., Gandy A.S. (2021). High-Entropy Alloys for Advanced Nuclear Applications. Entropy.

[190] Zhang L., Zhang P., Li N., Zhang X., Mei X. (2024). Dislocation evolution and hardening of CoCrFeMnNi high entropy alloy under Fe ion irradiation at room temperature and 500 °C. J. Appl. Phys..

[191] Kumar N.A.P.K., Li C., Leonard K.J., Bei H., Zinkle S.J. (2016). Microstructural stability and mechanical behavior of FeNiMnCr high entropy alloy under ion irradiation. Acta Mater..

[192] Li M., Lin F., Zhang S., Zhao R., Tao L., Li L., Li J., Zeng L., Luo M., Guo S. (2024). High-entropy alloy electrocatalysts go to (sub-)nanoscale. Sci. Adv..

[193] Löffler T., Savan A., Meyer H., Savan A., Wilde P., Manjón A.G., Chen Y., Ventosa E., Scheu C., Ludwig A. (2018). Discovery of a Multinary Noble Metal-Free Oxygen Reduction Catalyst. Adv. Energy Mater..

[194] Glasscott M.W., Pendergast A.D., Goines S., Bishop A.R., Hoang A.T., Renault C., Dick J.E. (2019). Electrosynthesis of High-Entropy Metallic Glass Nanoparticles for Designer, Multi-Functional Electrocatalysis. Nat. Commun..

[195] Hao J., Zhuang Z., Cao K., Gao G., Wang C., Lai F., Lu S., Ma P., Dong W., Liu T. (2022). Unraveling the electronegativity-dominated intermediate adsorption on high-entropy alloy electrocatalysts. Nat. Commun..

[196] Chida Y., Tomimori T., Ebata T., Taguchi N., Ioroi T., Hayashi K., Todoroki N., Wadayama T. (2023). Experimental study platform for electrocatalysis of atomic-level controlled high-entropy alloy surfaces. Nat. Commun..

[197] Wu C.-Y., Hsiao Y.-C., Chen Y., Lin K.-H., Lee T.-J., Chi C.-C., Lin J.-T., Hsu L.-C., Tsai H.-J., Gao J.-Q. (2024). A catalyst family of high-entropy alloy atomic layers with square atomic arrangements comprising iron- and platinum-group metals. Sci. Adv..

[198] Strotkötter V., Li Y., Kostka A., Lourens F., Löffler T., Schuhmann W., Ludwig A. (2024). Self-Formation of Compositionally Complex Surface Oxides on High Entropy Alloys Observed by Accelerated Atom Probe Tomography: A Route to Sustainable Catalysts. Mater. Horiz..

[199] Zhan C., Xu Y., Bu L., Zhu H., Feng Y., Yang T., Zhang Y., Yang Z., Huang B., Shao Q. (2021). Subnanometer high-entropy alloy nanowires enable remarkable hydrogen oxidation catalysis. Nat. Commun..

[200] Qiu H.-J., Fang G., Wen Y., Liu P., Xie G., Liu X., Sun S. (2019). Nanoporous High-Entropy Alloys for Highly Stable and Efficient Catalysts. J. Mater. Chem. A.

[201] Chen T., Ning F., Qi J., Feng G., Wang Y., Song J., Yang T., Liu X. (2023). PtFeCoNiCu High-Entropy Solid Solution Alloy as Highly Efficient Electrocatalyst for the Oxygen Reduction Reaction. iScience.

[202] He L., Li M., Qiu L., Ren X., Tian F., Sheng J., Liu Y., Luo M., Zhou X., Yu Y. (2025). Breaking the Symmetry of High-Entropy Alloy Surfaces for Compressively Strain-Tuned Oxygen Reduction Reaction. Nat. Commun..

[203] Chen T., Zhang X., Wang H., Yuan C., Zuo Y., Gao C., Xiao W., Yu Y., Cai J., Luo T. (2025). Antisite Defect Unleashes Catalytic Potential in High-Entropy Intermetallics for Oxygen Reduction Reaction. Nat. Commun..

[204] Zhao X., Cheng H., Wu L., Zhang Q., Chen X., Marinkovic N., Li C., Tan S., Hu E., Ma L. (2025). Sub-Angstrom Strain in High-Entropy Intermetallic Boosts the Oxygen Reduction Reaction in Fuel Cell Cathodes. Nat. Commun..

[205] He G., Zhang X., Liu J., Chong J., Ye G., Fei H. (2025). Hydrocarbothermal Flow Synthesis of Carbon-Supported Small and Dense High-Entropy Alloy Nanoparticles as Electrocatalysts. Nat. Commun..

[206] Feng G., Ning F., Song J., Shang H., Zhang K., Ding Z., Gao P., Chu W., Xia D. (2021). Sub-2 nm Ultrasmall High-Entropy Alloy Nanoparticles for Extremely Superior Electrocatalytic Hydrogen Evolution. J. Am. Chem. Soc..

[207] Wu C., Zhang X., Zhang Y., Ma W., Zhao D., Ren B., Zhang Z., Wang Y. (2025). Mechanical alloyed FeCoNiMoM (M = Cr, Cu) high-entropy alloy powders as electrocatalysts for oxygen evolution reaction. J. Mater..

[208] Yang P., Jiang Z., Shi Y., Ren X., Liang L., Shao Q., Zhu K. (2023). Enhancement of oxygen evolution reaction performance of FeCoNiCrMn high entropy alloy thin film electrodes through in-situ reconstruction. J. Alloys Compd..

